# Trends in the prevalence and pharmacological management of migraine during pregnancy in the UK, 2000–2018

**DOI:** 10.1136/jnnp-2024-333530

**Published:** 2024-04-03

**Authors:** Katherine Phillips, Krishnarajah Nirantharakumar, Benjamin R Wakerley, Francesca L Crowe

**Affiliations:** 1 Institute of Applied Health Research, University of Birmingham, Birmingham, UK; 2 Midlands Health Data Research UK, University of Birmingham, Birmingham, UK; 3 Institute of Metabolism and Systems Research, University of Birmingham, Birmingham, UK; 4 Department of Neurology, University Hospitals Birmingham NHS Foundation Trust, Birmingham, UK

**Keywords:** MIGRAINE

## Abstract

**Background:**

Migraine is common in women of reproductive age. This study aimed to (1) describe the prevalence of migraine in pregnant women in the UK, (2) identify drugs commonly prescribed for migraine during pregnancy and (3) identify characteristics associated with being prescribed medication for migraine during pregnancy.

**Methods:**

The Clinical Practice Research Datalink pregnancy register, a database of pregnancy episodes identified in anonymised primary care health records, was used.

Crude and age-standardised prevalence of migraine during pregnancy and the proportion of women with migraine prescribed drugs used for migraine management were calculated for each year between 2000 and 2018.

Logistic regression was used to describe the relationship between patient characteristics and being prescribed migraine medication during pregnancy.

**Results:**

1 377 053 pregnancies were included, of which 187 328 were in women with a history of migraine. The age-adjusted prevalence increased from 11.4% in 2000 to 17.2% in 2018. There was an increase in the rates of prescription for numerous medications for the management of migraine.

Older women (adjusted OR (aOR) 1.41 (1.20 to 1.66)), women of black (aOR 1.40 (1.32 to 1.48)) and South Asian ethnicity (aOR 1.48 (1.38 to 1.59)), those living in the most deprived areas (aOR 1.60 (1.54 to 1.66)), women who were obese (aOR 1.39 (1.35 to 1.43)), smokers (aOR 1.15 (1.12 to 1.18)) and those with comorbid conditions were more likely to receive a prescription during pregnancy.

**Conclusions:**

Rates of recorded migraine have increased over the past two decades as well as rates of prescribing in women with migraine. Higher prescribing rates are seen in certain groups, which has the potential to exacerbate health inequalities.

**WHAT IS ALREADY KNOWN ON THIS TOPIC**

- Migraine is a common condition in women of reproductive age.
- It is associated with an increased risk of pregnancy complications such as pre-eclampsia.
- Medication use is common during pregnancy in women with migraine.

**WHAT THIS STUDY ADDS**

- Rates of recorded pre-pregnancy migraine in the UK have risen over the past two decades.
- Prescriptions of medications for the management of migraine have also increased.
- Higher prescription rates were seen in groups already at higher risk of pregnancy complications, including women who were older, from minority ethnic backgrounds, living in deprived areas, were overweight or obese, smoked or had comorbid conditions.

**HOW THIS STUDY MIGHT AFFECT RESEARCH, PRACTICE OR POLICY**

- Further work is needed to understand how migraine and its subtypes impact on pregnancy outcomes.
- This study highlights the need for further research into the safety of drugs for the treatment of migraine in pregnancy.

## Background

Migraine is a common condition in women, particularly during the reproductive years with a prevalence of 18.6% among women aged 20–64 years reported in a 2020 meta-analysis.^1^ It is the leading cause of disability among people under 50 years and contributes 45.1 million years lived with disability to the global disease burden.^2^ People who experience migraines report a negative impact on their education, employment and family relationships.^2^

Global rates of migraine are thought to have remained stable over time,^3^ particularly in high-income countries,^4^ with prevalence peaking in those aged 35–39 years.^2^ As the average maternal age is increasing in high-income countries,^5^ it is likely that the burden of migraine during pregnancy is also increasing. There is evidence that migraine increases the risk of pregnancy complications such as pre-eclampsia^6^ and preterm birth^7^ so it is important to understand the burden of migraine during pregnancy.

Migraine often requires pharmacological therapy to both manage and prevent episodes,^8^ and medication use has been found to be common during pregnancy in women with migraine.^9^ There is a paucity of evidence around the safety of some migraine drugs in pregnancy. A systematic review and meta-analysis of studies of the prevalence of adverse pregnancy outcomes in migraine-treated women found no increased risk associated with triptans, but insufficient evidence to evaluate the safety of other drugs used in the management of migraines.^10^ Understanding which medications are most commonly prescribed for migraine during pregnancy will help direct priorities for future drug safety studies.

The aims of this study were (1) to estimate the annual prevalence of migraine in pregnant women between 2000 and 2018 in the UK; (2) to identify commonly prescribed drugs among pregnant women with migraine during the first trimester and throughout the whole pregnancy; and (3) to describe the characteristics associated with being prescribed medication in the treatment of migraine during pregnancy.

## Methods

### Study design

A pregnancy cohort study of women with migraine was conducted to estimate annual point prevalence estimates of migraine and to identify common prescription drugs for migraine during pregnancy.

### Data source

The Clinical Practice Research Datalink (CPRD) GOLD is a UK primary care database containing the anonymised medical records of over 20 million patients. It covers approximately 7% of the UK population and is comparable in terms of age and sex with the general population. An algorithm has been developed and validated within CPRD GOLD to create a pregnancy register using pregnancy-related Read codes, a hierarchical clinical coding system, used to document symptoms, diagnoses and referrals. Prescriptions issued in primary care are recorded using drug codes.

### Definition of study population

All women aged between 15 and 50 years in the CPRD GOLD pregnancy register with pregnancies that occurred between 2000 and 2018 formed the source population. Eligibility to enter the study began when participants fulfilled the following criteria: (1) acceptable patient flag, (2) minimum 1 year of registration with a practice or 1 year after up-to-standard date of the registered practice whichever was the latest date. All eligible women in the CPRD pregnancy register were included in the denominator for the estimation of prevalence trends for migraine.

For the study of prescribing trends and outcomes recorded in primary care, the study population was all eligible women from the pregnancy cohort who also had a coded diagnosis of migraine or a prescription for medications used exclusively in the treatment of migraine prior to their pregnancy start date.

### Definition of variables

The exposure was the presence of a coded diagnosis of migraine or a prescription for medications used exclusively in the treatment of migraine (triptans, calcitonin gene-related peptide inhibitors and migraine combination drugs (acetylsalicylic acid or paracetamol, combined with codeine, caffeine and/or an antiemetic)) prior to their pregnancy start date.

To describe trends in prescribing patterns, the presence of a code for the prescription of the following drugs used in the treatment of migraine was used:

- Amitriptyline
- Acetylsalicylic acid
- Antiemetics (prochlorperazine, metoclopramide, domperidone, cyclizine)
- Beta-blockers
- Calcitonin gene-related peptide inhibitors
- Candesartan
- Duloxetine
- Flunarizine
- Migraine combination drugs
- Mirtazapine
- Non-steroidal anti-inflammatory drugs (NSAIDs) (diclofenac, ibuprofen, naproxen, tolfenamic acid, mefenamic acid)
- Paracetamol
- Pizotifen
- Sodium valproate
- Topiramate
- Triptans
- Venlafaxine

Codelists used to define diagnoses and prescriptions are included in online supplemental table 1.

10.1136/jnnp-2024-333530.supp4 Supplementary data

### Statistical analysis

#### Estimation of prevalence and incidence trends

Annual prevalence of migraine before pregnancy was calculated for each year between 2000 and 2018 by dividing the number of women meeting the migraine exposure definition whose pregnancy started within the given year by the total number of pregnancies that started within the year.

Age-standardised prevalence was calculated using the direct method. Prevalence rates for each year were applied to the European Standard Population 2013.^11^

### Analysis of prescription trends

For each annual cohort, the number of women with a recorded prescription for a drug used in the treatment of migraine during pregnancy was divided by the total number of pregnancies of women with migraine that started within that year. This was stratified by acute drugs (pain relief, antiemetics, combinations, triptans) and prophylactic drugs (beta-blockers, topiramate, amitriptyline, candesartan, sodium valproate, flunarizine, pizotifen, calcitonin gene-related peptide inhibitors). This analysis was performed for prescriptions given over the whole pregnancy and restricting to those given in the first trimester.

### Analysis of characteristics associated with receiving a prescription

Logistic regression was used to estimate ORs, adjusted ORs (aORs) and 95% CIs to describe the relationship between patient characteristics (age, ethnicity, deprivation, body mass index (BMI), smoking status and comorbidities (asthma, chronic kidney disease depression, endometriosis, hypertension, hyperthyroidism, hypothyroidism, inflammatory bowel disease (IBD), polycystic ovarian syndrome (PCOS), systemic lupus erythematosus (SLE), type 1 diabetes, type 2 diabetes and epilepsy)) and being prescribed any medication used in the management of migraine during pregnancy.

All analyses were performed in Stata IC V.17 (StataCorp).

## Results

### Prevalence of migraine before pregnancy

There were 1 377 053 pregnancies including 769 024 women in the CPRD pregnancy register who met the data quality criteria. 187 328 pregnancies and 98 932 women had either a coded diagnosis of migraine or had been issued a prescription for drugs used exclusively in the management of migraine prior to pregnancy.

The median age at the start of pregnancy for women both with and without migraine was 28.9 years. A slightly higher proportion of pregnancies in women with migraine were in women who were white (48.1% vs 45.0%) and a slightly higher proportion of pregnancies in women without migraine were in women who had no ethnicity recorded (47.0% vs 44.3%). A higher proportion of pregnancies in women with migraine were in women who were overweight or obese (21.9% vs 19.5% and 18.0% vs 13.4%, respectively) (table 1).

**Table 1:** Baseline characteristics

	Pregnancies of women with migraine	Pregnancies of women without migraine
Number of pregnancies, n	187 328	1 189 725
Number of women, n	98 932	670 092
Age at start of pregnancy, median (IQR)	28.9 (24.2–33.2)	28.9 (23.8–33.2)
Age categories, number of pregnancies (percentage of pregnancies)		
15–19 years	15 005 (8.0)	138 245 (11.6)
20–24 years	38 306 (20.5)	231 699 (19.5)
25–29 years	53 059 (28.3)	304 102 (25.6)
30–34 years	48 681 (26.0)	308 110 (25.9)
35–39 years	25 362 (13.5)	165 741 (13.9)
40–44 years	6211 (3.3)	38 284 (3.2)
45–49 years	704 (0.4)	3544 (0.3)
Ethnicity, number of pregnancies (percentage of pregnancies)		
White	90 109 (48.1)	532 664 (45.0)
South Asian	4328 (2.3)	34 511 (2.9)
Black	5786 (3.1)	33 288 (2.8)
Mixed ethnicity	856 (0.5)	7504 (0.6)
Others	2885 (1.5)	19 547 (1.6)
Missing	83 364 (44.3)	559 211 (47.0)
IMD, number of pregnancies (percentage of pregnancies)		
1 (most deprived)	41 284 (22.0)	239 881 (20.2)
2	32 538 (17.4)	202 749 (17.0)
3	30 887 (16.5)	198 538 (16.7)
4	27 012 (14.4)	173 129 (14.6)
5 (least deprived)	28 895 (15.4)	192 330 (16.2)
Missing	26 712 (14.3)	183 098 (15.4)
BMI (kg/m^2^), number of pregnancies (percentage of pregnancies)		
Underweight (<18.5)	6489 (3.5)	42 858 (3.6)
Normal weight (18.5–<25)	78 858 (42.1)	518 522 (43.6)
Overweight (25–<30)	41 109 (21.9)	232 171 (19.5)
Obese (≥30)	33 714 (18.0)	159 342 (13.4)
Missing	27 158 (14.5)	236 802 (19.9)
Smoking status, number of pregnancies (percentage of pregnancies)		
Non-smoker	97 305 (51.9)	626 159 (52.6)
Ex-smoker	29 013 (15.5)	161 622 (13.6)
Smoker	54 374 (29.0)	323 356 (27.2)
Missing	6636 (3.5)	78 587 (6.6)

BMI, body mass index; IMD, Index of Multiple Deprivation.

**Figure 1:**
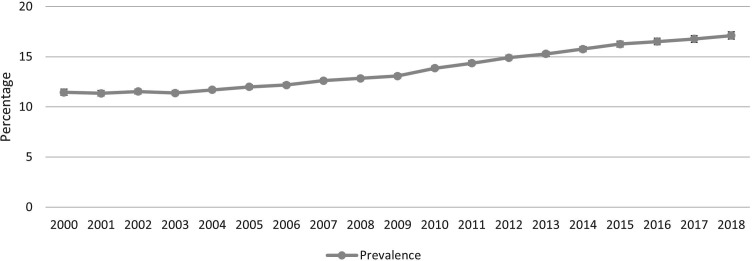
Prevalence of migraine in the CPRD Gold pregnancy register 2000–2018. CPRD, Clinical Practice Research Datalink.

**Figure 2:**
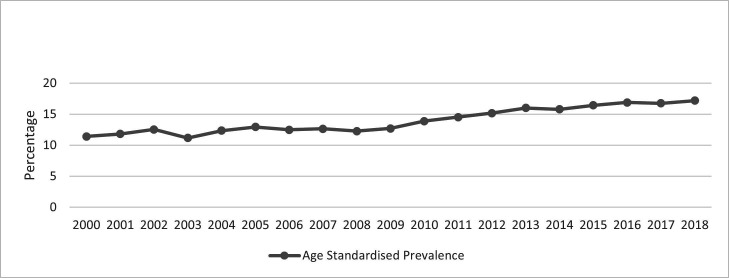
Age-standardised prevalence of migraine in the CPRD Gold pregnancy register 2000–2018. CPRD, Clinical Practice Research Datalink.

The overall crude prevalence of migraine in the cohort was 13.7% (95% CI 13.6% to 13.8%). The prevalence increased from 11.5% (95% CI 11.2% to 11.8%) in 2000 to 17.1% (95% CI 16.7% to 17.5%) in 2018 (figure 1 and online supplemental table 2). The age-adjusted prevalence increased from 11.4% (95% CI 10.3% to 12.4%) in 2000 to 17.2% (95% CI 16.2% to 18.2%) in 2018 (figure 2 and online supplemental table 3).

10.1136/jnnp-2024-333530.supp5 Supplementary data

10.1136/jnnp-2024-333530.supp6 Supplementary data

### Acute medications

**Figure 3:**
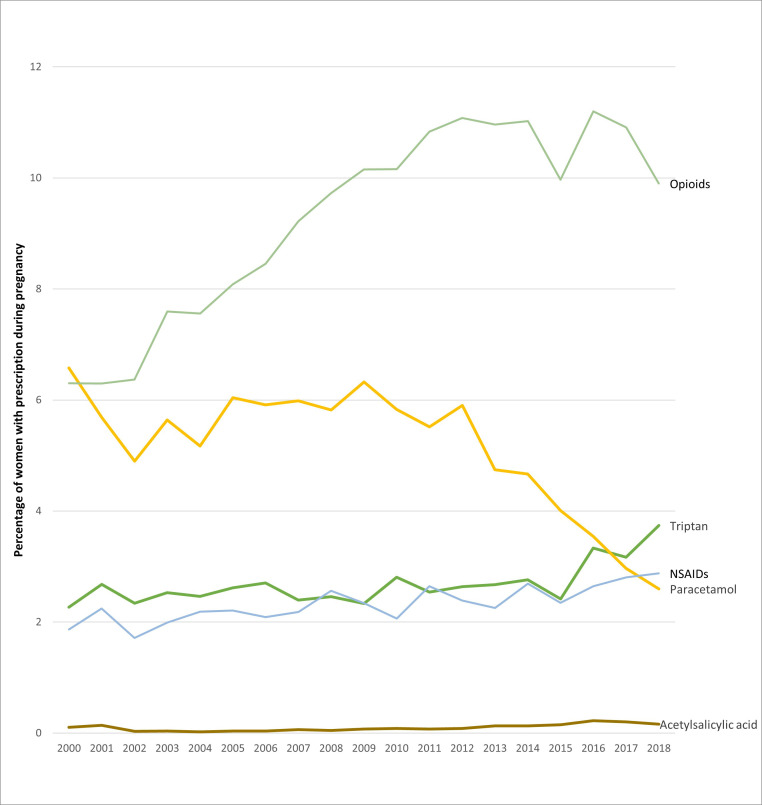
Prevalence of prescriptions for the acute management of migraine, 2000–2018. NSAIDs, non-steroidal anti-inflammatory drugs.

Between 2000 and 2018, the proportion of pregnant women prescribed triptans increased from 2.27% (n=108) to 3.74% (n=256) throughout pregnancy and from 2.04% (n=97) to 3.39% (n=232) in the first trimester. Over the same time, the proportion prescribed paracetamol decreased from 6.58% (n=313) to 2.60% (n=178) throughout pregnancy and from 3.19% (n=152) and 1.58% (n=108) in the first trimester. Prescriptions of opiates (co-codamol, codeine and tramadol) increased before plateauing around 2014 (figure 3 and online supplemental figure 1).

10.1136/jnnp-2024-333530.supp1 Supplementary data

### Antiemetics

**Figure 4:**
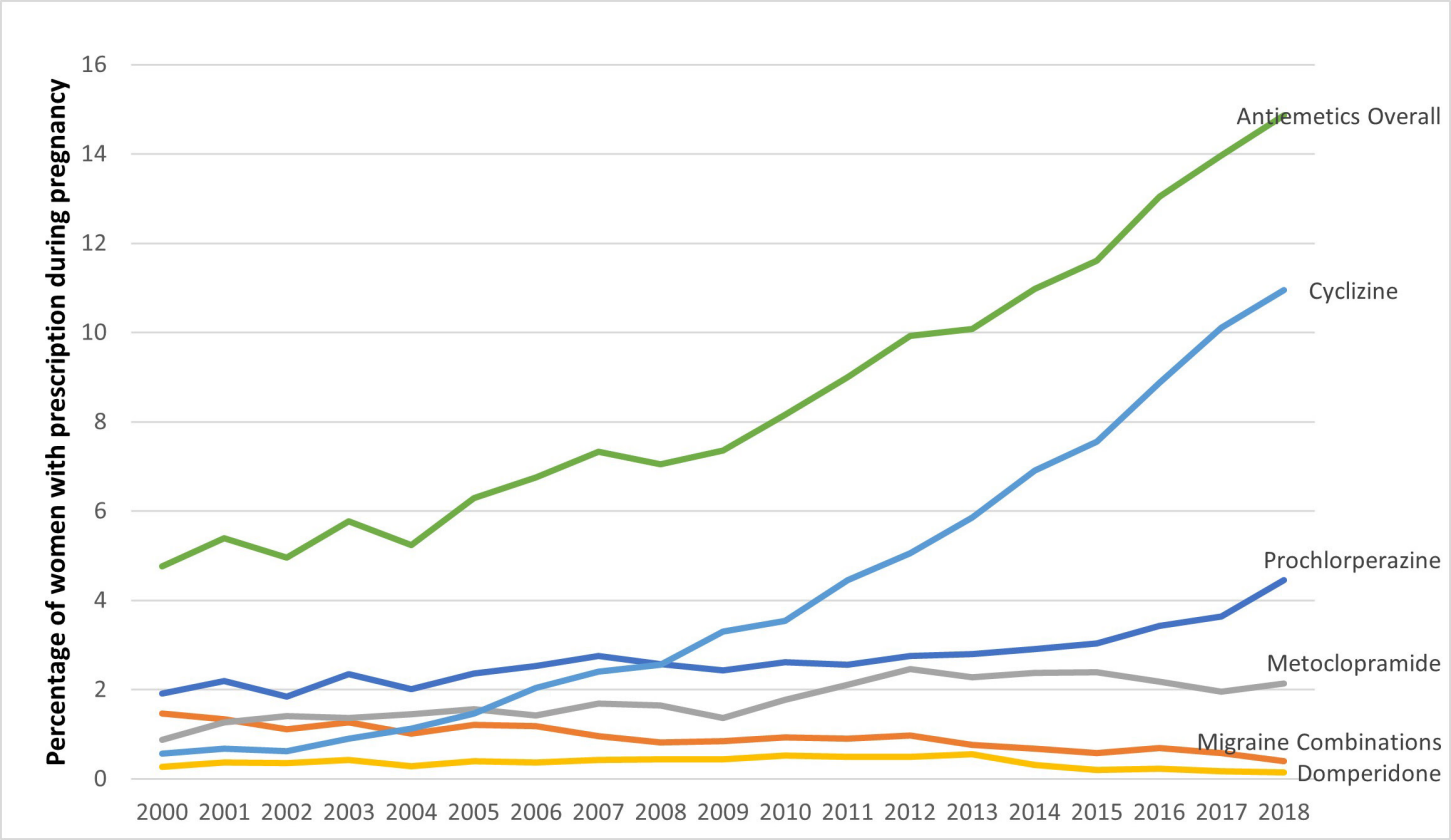
Prevalence of prescriptions for antiemetics in patients with migraine, 2000–2018.

Between 2000 and 2019, the proportion of pregnant women prescribed cyclizine increased from 0.57% (n=27) to 10.95% (n=750) throughout pregnancy and from 0.48% (n=23) to 8.99% (n=616) in the first trimester. The proportion prescribed prochlorperazine increased from 1.91% (n=91) to 4.45% (n=305) throughout pregnancy and from 1.39% (n=66) to 3.43% (n=235) in the first trimester. Prescription of metoclopramide, migraine combination drugs and domperidone remained stable over the time period (figure 4 and online supplemental figure 2).

10.1136/jnnp-2024-333530.supp2 Supplementary data

### Prophylactic medications

**Figure 5:**
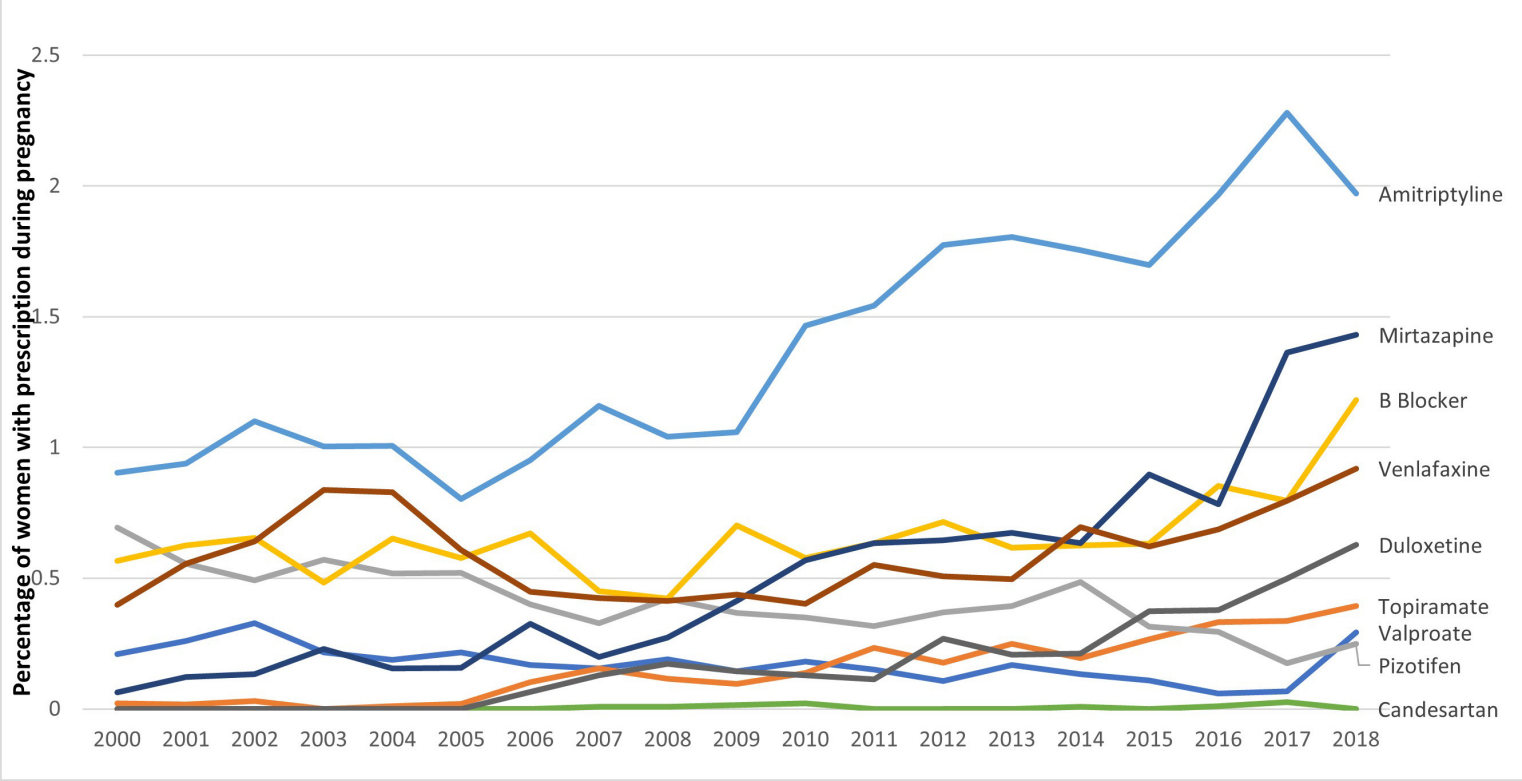
Prevalence of prescriptions for the prophylactic management of migraine, 2000–2018.

Throughout pregnancy, rates of prescriptions for amitriptyline, mirtazapine, venlafaxine and duloxetine increased from 0.90% (n=43) to 1.97% (n=135), 0.06% (n=3) to 1.43% (n=98), 0.40% (n=19) to 0.92% (n=63) and 0% to 0.63% (n=43), respectively. In the first trimester, rates of prescriptions for amitriptyline, mirtazapine, venlafaxine and duloxetine increased from 0.74% (n=35) to 1.81% (n=124), 0.06% (n=3) to 1.33% (n=91), 0.40% (n=19) to 0.88% (n=60) and 0% (n=0) to 0.55% (n=38), respectively. The prescription rates of beta-blockers increased from 0.57% (n=27) to 1.18% (n=81) throughout pregnancy and 0.53% (n=25) to 1.14% (n=78) (figure 5 and online supplemental figure 3).

10.1136/jnnp-2024-333530.supp3 Supplementary data

### Characteristics associated with receiving a prescription

Women aged 45–49 years were at significantly higher odds of being prescribed drugs used in migraine (aOR 1.41 (95% CI 1.20 to 1.66)) compared with those aged 25–29 years, whereas those aged 15–19 years were at significantly lower odds (aOR 0.86 (95% CI 0.82 to 0.90)).

Compared with women of white ethnicity, women of Black ethnicity and South Asian ethnicity were at significantly higher odds of being prescribed drugs used in migraine (aOR 1.40 (95% CI 1.32 to 1.48) and 1.48 (95% CI 1.38 to 1.59), respectively).

Compared with women living in the least deprived areas, women in the most deprived areas were at significantly higher odds of receiving a prescription (aOR 1.60 (95% CI 1.54 to 1.66)). Compared with women with a record of normal pre-gravid BMI, women who were overweight or obese were at significantly higher odds of receiving a prescription (aOR 1.12 (95% CI 1.09 to 1.16) and 1.39 (95% CI 1.35 to 1.43)). Compared with non-smokers, women who smoked were at higher odds of being prescribed medications used in migraine (aOR 1.15 (95% CI 1.12 to 1.18)).

**Figure 6:**
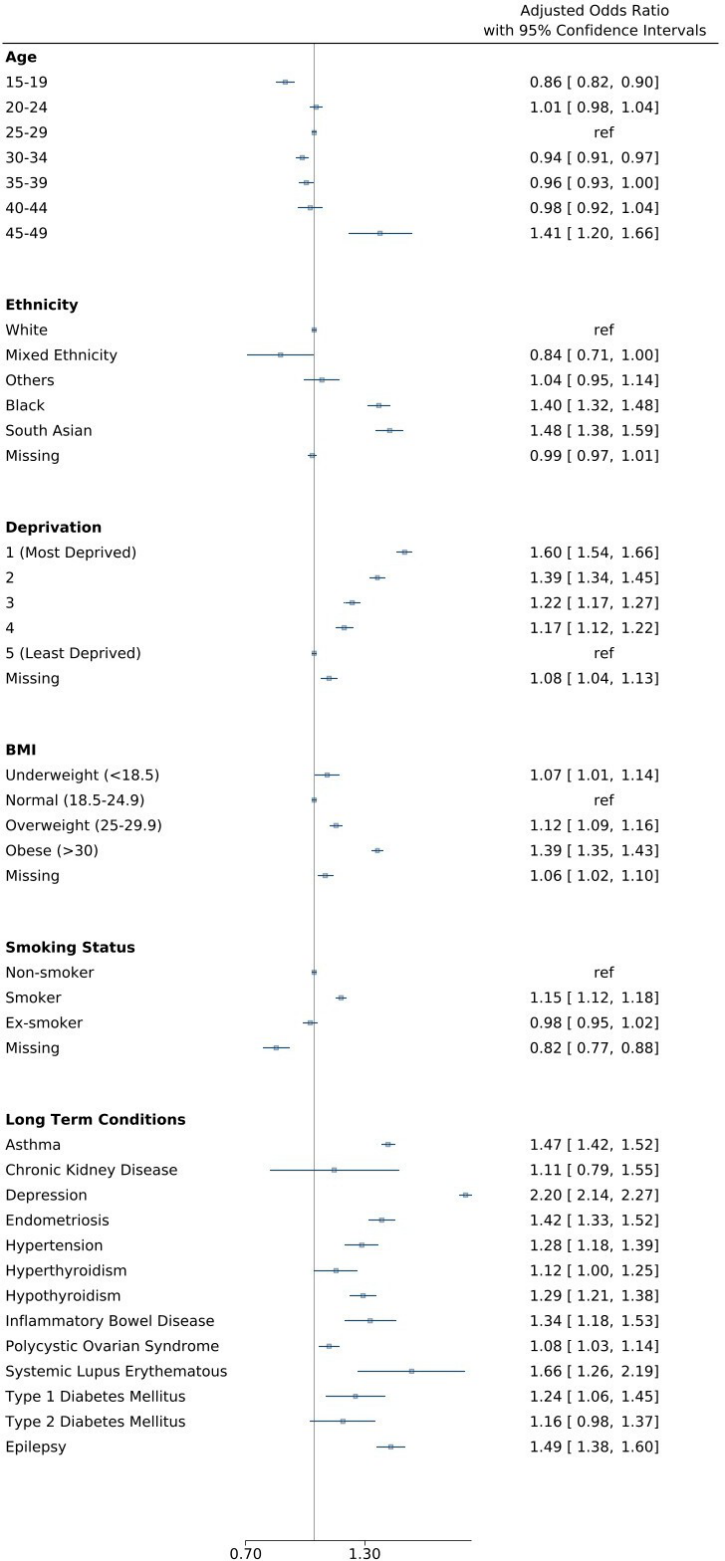
Factors associated with receiving a prescription for medications used in migraine during the whole pregnancy.

Women with asthma, depression, endometriosis, hypertension, hyperthyroidism, hypothyroidism, IBD, PCOS, SLE, type 1 diabetes and epilepsy were also at significantly increased odds of receiving a prescription (figure 6).

## Discussion

### Key results

The recorded prevalence of migraine increased between 2000 and 2018. There was an increase in the rates of prescription for numerous medications for the management of migraine, notably, triptans, antidepressants (including amitriptyline) and beta-blockers. Older women, women of black and South Asian ethnicity, those living in the most deprived areas, women who were overweight or obese, smokers and those with comorbid conditions were more likely to receive a prescription during pregnancy.

### Strengths and limitations

There are several strengths to this study, including that the cohort came from a large dataset that is generalisable to the UK population. To our knowledge, this is the first such study to describe trends in the prevalence of migraine and migraine medications during pregnancy. The high prevalence of migraine during pregnancy highlights the importance of understanding how migraine and its associated treatments impact pregnancy outcomes.

The limitations of this study include the likelihood that migraine is unrecorded in primary care as not all cases of migraine will require medical input. As discussed below, the prevalence of migraine found was lower than would be expected in women of reproductive age.^1^ We attempted to improve the accuracy of our prevalence estimate by including medications used exclusively to manage migraine in our phenome definition. Despite this, the prevalence is likely affected by under-reporting. As trends in migraine are thought to have remained stable over time, it is possible that this particularly affected the earlier years and that recording of migraine has improved over time.

A limitation of using prescription data is that it is unknown whether medications were taken. Medications that can be purchased over the counter would also not have been captured, which would have led to the undercounting of medications such as paracetamol. We cannot be certain of the indication for the medications studied. Many of the medications considered are also used to manage conditions other than migraine. For instance, duloxetine, mirtazapine and venlafaxine may be used for depression; topiramate and valproate may be used for epilepsy; and beta-blockers and candesartan may be used for hypertension. This may account for the significant increase in the odds of being prescribed medications associated with these conditions.

### Findings in the context of other literature

The prevalence of migraine in 2018 is similar to the prevalence found in a Global Burden of Disease (GBD) Study in 2019 (18.6% inwomen aged 20–64 years),^1^ although the overall prevalence for the time period included in this study is lower. The prevalence estimates from studies included in the GBD meta-analysis came from questionnaires or interviews. As many patients with migraine will self-manage and not need to consult with their general practitioner (GP), this may explain the apparent undercount in this study.

The overall prescription rate for triptans during the whole of pregnancy was 2.7%. This was lower than the prevalence of 25% found in a Norwegian study, although this study cohort composed of those already taking triptans prior to pregnancy.^12^ When considering women who had been prescribed triptan in the year before pregnancy, however, 16% of women in our cohort received a prescription for triptans during pregnancy.

The prevalence for prescriptions of triptans was 2.5% for the first trimester. This was lower than the rate found in a US cohort (15.1%).^9^ However, this study employed much stricter criteria for defining migraine, meaning that the prevalence in the study was only about 1% prevalence of migraine. This is likely to represent the most severe cases.

The increase in the prevalence of antidepressant prescription is in line previous reports.^13^ To our knowledge, this has not been described in the migraine population specifically.

A Canadian study found an increase in the prescription of labetalol (likely to reflect prescribing for pregnancy-induced hypertension) but a decline in the prescription of other beta-blockers.^14^

This study is in keeping with the findings of a study of rates of polypharmacy during pregnancy in the same population. This study found being older, overweight and obese, of black and South Asian ethnicity and smoking were all risk factors for polypharmacy.^15^ Our study found that having comorbidities was associated with increased rates of prescribing. This is in agreement with the findings of a US study of a health insurance database looking at medication use throughout pregnancy in around 8000 women with migraine, which found complex comorbidity was also associated with a higher prescription rate.^9^

### Interpretation

The increase in the prevalence of migraine seen in this study might reflect a true increase in prevalence or an increase in the number of women accessing general practice for management of migraine. The increase in prescription rates may therefore partly reflect an increase in the need for migraine treatment and guidance becoming less precautionary around prescribing certain medications during pregnancy. National Institute for Health and Care Excellence guidance has advised that triptans can be considered in the management of migraine during pregnancy since 2012.^16^ Amitriptyline and beta-blockers are thought to be safe at low doses and have been recommended in pregnancy. On the other hand, guidance for the use of NSAIDs during pregnancy has become more precautionary over this time period. In 2002, the British Society of Rheumatology and British Health Professionals in Rheumatology advised that NSAIDs were considered safe in the first trimester, but in 2016 advised that they be used with caution. Due to concerns that NSAIDs were associated with premature closure of the ductus arteriosus, it has recently been advised that they should not be taken after 20 weeks of pregnancy (having previously been thought to be safe until 32 weeks).^17^ ^18^ ^19^ Despite this, an increase in the prevalence of prescribing was seen over the study time period.

If, over time, women have been encouraged to book early with their GP or maternity services to ensure antenatal care is commenced in a timely way,^20^ ^21^ this would increase the number of women seeking care earlier in pregnancy when pregnancy symptoms, such as nausea and vomiting, or migraine episodes are more likely.^22^ This may have contributed to the increasing trends in prescriptions for antiemetics and pain relief.

Prescriptions were found to be more likely in women who are already at higher risk of adverse outcomes. Women aged 45–49 years were at a 40% increased risk of being prescribed medications. Advanced maternal age is associated with an increased risk of fetal growth restriction, pre-eclampsia, placental abruption, preterm birth and stillbirth.^23^ In England, being of black or South Asian ethnicity or living in a socioeconomically deprived area has been found to be associated with increased risk of stillbirth, preterm births and births with fetal growth restriction.^24^ Women with obesity and comorbidities in addition to migraine had higher rates of prescription. Maternal obesity is associated with gestational diabetes, pre-eclampsia, preterm birth and large for gestational age babies (weight >90th centile for gestational age),^25^ and pre-pregnancy multimorbidity is also associated with increased rates of maternal morbidity and mortality.^26^ Any harms associated with these medications will therefore contribute to the risk of adverse outcomes in these groups.

### Implications for research and practice

Migraine is commonly classified as being with and without aura.^27^ As those with aura have been found to have a higher risk of stroke,^28^ it may be important to understand the burden of migraine with aura during pregnancy. This was explored in this dataset, but only a small proportion of the population (~3.5%) had aura status recorded.

Apart from triptans, there is a paucity of evidence around the safety of migraine drugs during pregnancy.^10^ ^29^ Due to the exclusion of pregnant women from most clinical trials, much of the existing evidence for the effect of medication use during pregnancy comes from observational studies The inherent limitations and biases of these studies can mean it is difficult for clinicians and women to make decisions around medication use in pregnancy. This study highlights the increasing burden of pharmacological therapies in women with migraine during pregnancy and therefore the urgent need for further research into drug safety in pregnancy. Pregnant women have been historically excluded from clinical trials. More recently, there have been calls for their inclusion.^30^ This study provides further justification of the need for this.

The increasing trend in medication use during pregnancy in women with migraine also raises concerns about medication overuse headache. The prevalence of this condition during pregnancy and impact on pregnancy outcomes are not well understood and warrant further study.^31^

## Conclusion

The recorded prevalence of migraine has increased during pregnancy over the last two decades, as has the rate of prescribing in pregnant women with migraine. Certain groups are at higher risk of being prescribed medication, meaning any adverse effects could exacerbate health inequalities.

## Data Availability

Data may be obtained from a third party and are not publicly available. The data that support the findings of this study are available from CPRD but restrictions apply to the availability of these data, which were used under licence for the current study, and so are not publicly available.
