# Supplementary material for: Trends in the prevalence and pharmacological management of migraine during pregnancy in the UK, 2000–2018
Source: J Neurol Neurosurg Psychiatry. 2024 Apr 3;95(10):938–46. doi: 10.1136/jnnp-2024-333530 (PMC11420713; doi:10.1136/jnnp-2024-333530)
Supplement: Supplementary data [file jnnp-2024-333530supp004.pdf]

Codelists for migraine and comorbidities

DESCRIPTION	READ_CODE
Migraine	F26..00
Periodic migrainous neuralgia	F262500
Common migraine	F261.00
Abdominal migraine	F262200
Classical migraine	F260.00
Hemiplegic migraine	F26y000
H/O: migraine	1474
Migraine variants	F262.00
Abdominal migraine - symptom	1967
Basilar migraine	F262300
Atypical migraine	F261000
Ophthalmic migraine	F262400
Migraine - menstrual	K584.11
Ophthalmoplegic migraine	F26y100
Migraine NOS	F26z.00
[D]Abdominal migraine	R090D00
Status migrainosus	F26y200
Migraine variant NOS	F262z00
Complicated migraine	F26y300
Other forms of migraine	F26y.00
Other forms of migraine NOS	F26yz00
Common migraine NOS	F261z00
[X]Other migraine	Fyu5300
Moebius' ophthalmoplegic migraine	F26y111
H/O migraine with aura	1474000
Migraine with aura	F260.11
Migraine without aura	F261.11
Migraine induced by oestrogen contraceptive	F262800
DESCRIPTION	READ_CODE
Asthma	H33..00
Asthma monitoring	663..11
Acute exacerbation of asthma	H333.00
Asthma attack	H33z100
Severe asthma attack	H33z011
H/O: asthma	14B4.00
Childhood asthma	H330.12
Bronchial asthma	H33..11
Allergic asthma	H330.11
Mild asthma	663V100
Severe asthma	663V300
Occasional asthma	663V000
Late onset asthma	H331.11
Asthma unspecified	H33z.00

Exercise induced asthma	H33zz11
Status asthmaticus NOS	H33z000
Patient in asthma study	9Q21.00
Intrinsic asthma	H331.00
Seen in asthma clinic	9N1d.00
Hay fever with asthma	H330011
Chronic asthmatic bronchitis	H312000
Exercise induced asthma	173A.00
Extrinsic asthma with asthma attack	H330111
Emergency admission	asthma
Extrinsic (atopic) asthma	H330.00
Asthma limiting activities	663P.00
Asthma prophylactic medication used	663W.00
Asthma management plan given	663U.00
Asthma disturbing sleep	663N.00
Pollen asthma	H330.14
Asthma attack NOS	H33z111
Asthma monitored	9OJA.11
Number of asthma exacerbations in past year	663y.00
Change in asthma management plan	66Y5.00
Step up change in asthma management plan	66Y9.00
Asthma annual review	66YJ.00
Asthma medication review	8B3j.00
Suspected asthma	1J70.00
Asthma - currently active	663j.00
Asthma resolved	2126200
Asthma trigger	178..00
Asthma confirmed	1O2..00
Refuses asthma monitoring	9OJ2.00
Excepted from asthma quality indicators: Informed dissent	9hA2.00
Asthma resolved	212G.00
Late-onset asthma	H33z200
Asthma severity	663V.00
Moderate asthma	663V200
Asthma not disturbing sleep	663O.00
Asthma not limiting activities	663Q.00
Asthma disturbs sleep frequently	663N200
Asthma follow-up	66YK.00
Extrinsic asthma without status asthmaticus	H330000
Hay fever with asthma	H330.13
Asthma NOS	H33zz00
Asthma monitoring admin.	9OJ..00
Asthma control step 2	8795

Asthma control step 1	8794
Asthma monitoring due	66YE.00
Step down change in asthma management plan	66YA.00
Asthma control step 3	8796
Intrinsic asthma with asthma attack	H331111
Exception reporting: asthma quality indicators	9hA..00
Asthma monitoring by nurse	66YQ.00
Asthma treatment compliance unsatisfactory	663p.00
Asthma treatment compliance satisfactory	663n.00
Asthma monitoring check done	9OJA.00
Asthma control step 5	8798
Asthma control step 4	8797
Allergic asthma NEC	H33zz12
Occupational asthma	173c.00
Emergency asthma admission since last appointment	663d.00
Asthma causes daytime symptoms 1 to 2 times per week	663u.00
Asthma restricts exercise	663e.00
Asthma monitor 3rd letter	9OJ6.00
Asthma monitor 2nd letter	9OJ5.00
Asthma monitor 1st letter	9OJ4.00
Asthma clinical management plan	8CR0.00
Mixed asthma	H332.00
Health education - asthma	679J.00
Asthma never causes daytime symptoms	663s.00
Asthma causes daytime symptoms most days	663v.00
Asthma never restricts exercise	663f.00
Asthma severely restricts exercise	########
Asthma sometimes restricts exercise	6.63E+02
Extrinsic asthma with status asthmaticus	H330100
Intrinsic asthma without status asthmaticus	H331000
Asthma control step 0	8793
Asthma monitoring admin.NOS	9OJZ.00
Asthma monitoring by doctor	66YR.00
Asthma causing night waking	663N000
Asthma monitor phone invite	9OJ8.00
Asthma night-time symptoms	66YP.00
Asthma causes daytime symptoms 1 to 2 times per month	663t.00
Asthma monitor verbal invite	9OJ7.00

Asthma never disturbs sleep	663O000
Asthma limits walking up hills or stairs	663w.00
Asthma limits walking on the flat	663x.00
Asthma disturbs sleep weekly	663N100
Wood asthma	H35y700
Asthma causes night symptoms 1 to 2 times per month	663r.00
Brittle asthma	H334.00
Aspirin induced asthma	1780
Absent from work or school due to asthma	66YC.00
Asthma monitor offer default	9OJ3.00
Asthma daytime symptoms	663q.00
Intrinsic asthma NOS	H331z00
Extrinsic asthma NOS	H330z00
Attends asthma monitoring	9OJ1.00
Asthma accident and emergency attendance since last visit	663m.00
Detergent asthma	H47y000
Does not have asthma management plan	66YZ.00
Intrinsic asthma with status asthmaticus	H331100
Work aggravated asthma	173d.00
Sequoiosis (red-cedar asthma)	H35y600
Asthma control test	38DL.00
Patient has a written asthma personal action plan	8CMA000
Health education - asthma self management	679J000
Asthma control questionnaire	38DT.00
Under care of asthma specialist nurse	9NNX.00
Health education - structured asthma discussion	679J100
Asthma review using Roy Colleg of Physicians three questions	66Yp.00
Mini asthma quality of life questionnaire	38DV.00
Asthma trigger - seasonal	1787
Asthma trigger - pollen	1781
Asthma causes symptoms most nights	66Yr.00
Asthma causes night time symptoms 1 to 2 times per week	66Yq.00
Asthma trigger - respiratory infection	1789
Asthma limits activities 1 to 2 times per month	663P000
Asthma trigger - exercise	178B.00
Asthma limits activities 1 to 2 times per week	663P100

Asthma trigger - warm air	1783
Health education - structured patient focused asthma discuss	679J200
Asthma trigger - animals	1786
Asthma never causes night symptoms	66Ys.00
Royal College Physician asthma assessment 3 question score	388t000
Asthma trigger - cold air	1788
Asthma trigger - airborne dust	178A.00
Asthma trigger - damp	1785
Asthma trigger - emotion	1784
Asthma trigger - tobacco smoke	1782
Asthma limits activities most days	663P200
[X] Adverse reaction to antiasthmatics	U60F611
Asthma self-management plan review	661N100
Asthma self-management plan agreed	661M100
Chronic asthma with fixed airflow obstruction	H335.00
Number days absent from school due to asthma in past 6 month	66Yu.00
Asthma management plan declined	66Yz000
Childhood Asthma Control Test	38QM.00
Asthma monitorng invit SMS (short message servce) txt messge	9OJB.00
Asthma monitoring invitation email	9OJC.00
Telehealth asthma monitoring	66Yz500
Asthma monitoring SMS text message 1st invitation	9OJB000
Severe asthma exacerbation risk assessment	38B8.00
At risk of severe asthma exacerbation	14Ok000
Asthma monitoring SMS text message 2nd invitation	9OJB100
Asthma monitoring SMS text message 3rd invitation	9OJB200
DESCRIPTION	READ_CODE
Chronic renal failure	K05..00
End stage renal failure	K050.00
Chronic kidney disease stage 4	1Z13.00
Chronic kidney disease stage 3	1Z12.00
Chronic kidney disease stage 5	1Z14.00
Chronic kidney disease stage 2	1Z11.00
Chronic renal impairment	1Z1..00
Except chronic kidney disease qual indic: Patient unsuitable	9hE0.00
Anaemia secondary to renal failure	D215.00
Chronic kidney disease monitoring	66i..00
Anaemia secondary to chronic renal	D215000

failure	
Chronic kidney disease stage 1	1Z10.00
Chronic kidney disease annual review	6AA..00
Chronic kidney disease monitoring first letter	9Ot0.00
Exc chronic kidney disease quality indicators: Inform dissen	9hE1.00
Exception reporting: chronic kidney disease quality indicato	9hE..00
End stage renal failure	K05..12
[X]Other chronic renal failure	Kyu2100
Chronic kidney disease monitoring telephone invite	9Ot4.00
Chronic kidney disease monitoring administration	9Ot..00
Chronic kidney disease monitoring second letter	9Ot1.00
Chronic kidney disease monitoring third letter	9Ot2.00
Chronic kidney disease monitoring verbal invite	9Ot3.00
Chronic kidney disease stage 1 with proteinuria	1Z17.00
Chronic kidney disease stage 3 with proteinuria	1Z1B.00
Chronic kidney disease stage 3A	1Z15.00
Chronic kidney disease stage 2 without proteinuria	1Z1A.00
Chronic kidney disease stage 4 with proteinuria	1Z1H.00
Chronic kidney disease stage 3 without proteinuria	1Z1C.00
CKD stage 3 with proteinuria	1Z1B.11
Chronic kidney disease stage 2 with proteinuria	1Z19.00
Chronic kidney disease stage 3A without proteinuria	1Z1E.00
CKD stage 3A without proteinuria	1Z1E.11
Chronic kidney disease stage 3B without proteinuria	1Z1G.00
Chronic kidney disease stage 3B with proteinuria	1Z1F.00
Chronic kidney disease stage 3B	1Z16.00
CKD stage 3B with proteinuria	1Z1F.11
CKD stage 3 without proteinuria	1Z1C.11
Chronic kidney disease stage 5 without proteinuria	1Z1L.00
Chronic kidney disease stage 4 without proteinuria	1Z1J.00
Chronic kidney disease stage 3A with proteinuria	1Z1D.00
Did not attend chronic kidney disease	9Ni9.00

monitoring clinic	
Chronic kidney disease stage 5 with proteinuria	1Z1K.00
CKD stage 3A with proteinuria	1Z1D.11
Chronic kidney disease stage 1 without proteinuria	1Z18.00
CKD stage 4 without proteinuria	1Z1J.11
CKD stage 5 without proteinuria	1Z1L.11
CKD stage 2 without proteinuria	1Z1A.11
CKD stage 2 with proteinuria	1Z19.11
CKD stage 1 with proteinuria	1Z17.11
CKD stage 5 with proteinuria	1Z1K.11
CKD stage 4 with proteinuria	1Z1H.11
Acute-on-chronic renal failure	K0E..00
CKD stage 3B without proteinuria	1Z1G.11
Chronic kidney disease stage 3	K053.00
Chronic kidney disease stage 4	K054.00
Chronic kidney disease	K05..13
Chronic kidney disease stage 5	K055.00
Chronic kidney disease stage 2	K052.00
Chronic kidney disease stage 1	K051.00
Chronic kidney disease self-management plan agreed	661M200
CKD with GFR category G3b & albuminuria category A2	1Z1Y.00
CKD with GFR category G3a & albuminuria category A1	1Z1T.00
CKD with GFR category G3a & albuminuria category A2	1Z1V.00
CKD with GFR category G4 & albuminuria category A2	1Z1b.00
CKD with GFR category G3a & albuminuria category A3	1Z1W.00
CKD with GFR category G3b & albuminuria category A1	1Z1X.00
CKD with GFR category G4 & albuminuria category A1	1Z1a.00
CKD with GFR category G5 & albuminuria category A2	1Z1e.00
CKD with GFR category G3b & albuminuria category A3	1Z1Z.00
CKD with GFR category G1 & albuminuria category A2	1Z1N.00
CKD with GFR category G1 & albuminuria category A1	1Z1M.00
CKD with GFR category G2 & albuminuria category A2	1Z1R.00
CKD with GFR category G5 & albuminuria category A1	1Z1d.00
CKD with GFR category G2 & albuminuria category A3	1Z1S.00

CKD with GFR category G2 & albuminuria category A1	1Z1Q.00
CKD with GFR category G5 & albuminuria category A3	1Z1f.00
CKD with GFR category G1 & albuminuria category A3	1Z1P.00
CKD with GFR category G4 & albuminuria category A3	1Z1c.00
CKD stage 1 without proteinuria	1Z18.11
Chronic kidney disease self-management plan review	661N200
DESCRIPTION	READ_CODE
Depressive disorder NEC	E2B..00
[X]Depression NOS	Eu32z11
Endogenous depression	E112.14
Anxiety with depression	E200300
Agitated depression	E135.00
Neurotic depression reactive type	E204.00
Brief depressive reaction	E290.00
Depressive psychoses	E11..12
Postnatal depression	E204.11
H/O: depression	1465
Puerperal depression	62T1.00
[X]Depressive episode	unspecified
Postviral depression	E2B0.00
[X]Depressive disorder NOS	Eu32z12
[X]Recurrent depressive disorder	Eu33.00
Chronic depression	E2B1.00
[X]Depressive episode	Eu32.00
[X]Postpartum depression NOS	Eu53012
Agitated depression	E112.11
[X] Reactive depression NOS	Eu32z14
Recurrent depression	E113700
Endogenous depression first episode	E112.12
[X]Other depressive episodes	Eu32y00
Endogenous depression - recurrent	E113.11
Endogenous depression first episode	E112.13
Single major depressive episode NOS	E112z00
[X]Single episode of reactive depression	Eu32.13
[X]Neurotic depression	Eu34113
[X]Mild anxiety depression	Eu41211
[X]Dysthymia	Eu34100
Reactive depressive psychosis	E130.00
[X]Depressive neurosis	Eu34111
[X]SAD - Seasonal affective disorder	Eu33.15
[X]Recurrent episodes of depressive reaction	Eu33.11

[X]Recurrent episodes of reactive depression	Eu33.13
[X]Single episode of depressive reaction	Eu32.11
Masked depression	E11z200
[X]Moderate depressive episode	Eu32100
[X]Severe depressive episode without psychotic symptoms	Eu32200
Single major depressive episode	E112.00
[X]Mild depression	Eu32400
[X]Atypical depression	Eu32y11
Seasonal affective disorder	E118.00
[X]Schizoaffective disorder	depressive type
[X]Major depression	recurrent without psychotic symptoms
[X]Endogenous depression without psychotic symptoms	Eu33211
[X]Mild depressive episode	Eu32000
[X]Mixed anxiety and depressive disorder	Eu41200
[X]Severe depressive episode with psychotic symptoms	Eu32300
Depression medication review	9H91.00
Depression annual review	9H90.00
[X]Postnatal depression NOS	Eu53011
Recurrent major depressive episodes	moderate
Recurrent major depressive episode	E113.00
Single major depressive episode	moderate
Single major depressive episode	severe
[X]Persistant anxiety depression	Eu34114
Single major depressive episode	mild
Prolonged depressive reaction	E291.00
[X]Recurrent severe episodes of psychotic depression	Eu33315
Psychotic reactive depression	E130.11
[X]Single episode of psychogenic depression	Eu32.12
Postnatal depression counselling	6G00.00
[X]Recurrent brief depressive episodes	Eu3y111
Depression resolved	212S.00
[X]Recurrent episodes of psychogenic depression	Eu33.12
[X]Post-schizophrenic depression	Eu20400
Senile dementia with depression	E002100
[X]Single episode major depression w'out psychotic symptoms	Eu32212

[X]Endogenous depression with psychotic symptoms	Eu33311
[X]Single episode of psychotic depression	Eu32313
[X]Single episode of major depression and psychotic symptoms	Eu32311
Recurrent major depressive episodes	severe
Recurrent major depressive episode NOS	E113z00
Recurrent major depressive episodes	severe
Atypical depressive disorder	E11y200
Presenile dementia with depression	E001300
[X]Prolonged single episode of reactive depression	Eu32z13
[X]Seasonal depressive disorder	Eu33.14
[X]Single episode of reactive depressive psychosis	Eu32314
Recurrent major depressive episodes	mild
[X]Recurrent depressive disorder	current episode moderate
[D]Postoperative depression	R007z13
[X]Recurrent depressive disorder	current episode mild
Depression interim review	9H92.00
Patient given advice about management of depression	8CAa.00
Depression - enhanced services administration	9k4..00
[X]Recurr severe episodes/psychogenic depressive psychosis	Eu33314
Single major depressive episode	severe
Referral for guided self-help for depression	8HHq.00
[X]Depressive conduct disorder	Eu92000
[X]Recurr severe episodes/major depression+psychotic symptom	Eu33313
[X]Recurr depress disorder cur epi severe without psyc sympt	Eu33200
Single major depressive episode	unspecified
[X]Schizoaffective psychosis	depressive type
Recurrent major depressive episodes	unspecified
Brief depressive reaction NOS	E290z00
[X]Monopolar depression NOS	Eu33z11
[X]Recurrent severe episodes/reactive depressive psychosis	Eu33316
[X]Schizophreniform psychosis	depressive type

Senile dementia with depressive or paranoid features NOS	E002z00
[X]Single episode agitated depressn w'out psychotic symptoms	Eu32211
On depression register	9HA0.00
Arteriosclerotic dementia with depression	E004300
[X]Recurrent depressive disorder	unspecified
Senile dementia with depressive or paranoid features	E002.00
Depression management programme	8BK0.00
Drug-induced depressive state	E02y300
[X]Recurrent depress disorder cur epi severe with psyc symp	Eu33300
[X]Other recurrent depressive disorders	Eu33y00
Depression monitoring administration	9Ov..00
[X]Single episode of psychogenic depressive psychosis	Eu32312
[X]Single episode of masked depression NOS	Eu32y12
[X]Single episode vital depression w'out psychotic symptoms	Eu32213
Depression - enhanced service completed	9k40.00
Depression monitoring first letter	9Ov0.00
Depression monitoring second letter	9Ov1.00
[X]Vital depression	recurrent without psychotic symptoms
Depression monitoring telephone invite	9Ov4.00
Depression monitoring verbal invite	9Ov3.00
Depression monitoring third letter	9Ov2.00
On full dose long term treatment depression - enh serv admin	9kQ..00
[X]Major depression	moderately severe
[X]Major depression	mild
[X]Major depression	severe without psychotic symptoms
[X]Major depression	severe with psychotic symptoms
[X]Single major depr ep	severe with psych
[X]Recurr major depr ep	severe with psych
[X]Antenatal depression	Eu32B00

Referral for guided self-help for depression declined	8IH5200
DESCRIPTION	READ_CODE
Endometriosis	K50..00
Adenomyosis	K50..11
Endometriosis NOS	K50z.00
Laparoscopic laser destruction of endometriosis	7E0D800
Chocolate cyst of ovary	K501.11
Internal endometriosis	K500000
Endometriosis of ovary	K501.00
Endometriosis of the intestine	K505.00
Adenomyosis of endometrium	K500111
Endometriosis of the pelvic peritoneum	K503.00
Endometriosis of the fallopian tube	K502.00
Endometriosis of cervix	K500200
Endometriosis of the bladder	K50y000
Endometriosis of the vagina	K504100
Endometriosis of uterus	K500.00
Endometriosis of uterus NOS	K500z00
Endometriosis of the pelvic peritoneum NOS	K503z00
Endometriosis of the umbilicus	K50y200
Other endometriosis	K50y.00
Endometriosis of the pouch of Douglas	K503100
Endometriosis of the rectovaginal septum	K504000
Endometriosis of the round ligament	K503300
[M]Stromal endometriosis	BBL1.11
Endometriosis in scar of skin	K506.00
Endometriosis of the rectum	K505200
Endometriosis of myometrium	K500100
Endometriosis of the colon	K505100
Endometriosis of the parametrium	K503200
[X]Other endometriosis	Kyu9000
Other endometriosis NOS	K50yz00
Endometriosis of the broad ligament	K503000
Endometriosis of the vulva	K50y300
Endometriosis of the lung	K50y100
Endometriosis of the rectovaginal septum and vagina	K504.00
Endometriosis of the appendix	K505000
Endometriosis of the rectovaginal septum and vagina NOS	K504z00
Endometriosis of the intestine NOS	K505z00
DESCRIPTION	READ_CODE

Hypertensive disease	G2...00
Essential hypertension	G20..00
Benign essential hypertension	G201.00
H/O: hypertension	14A2.00
Hypertension resolved	2126100
On treatment for hypertension	662O.00
Hypertension NOS	G20z.11
Hypertensive encephalopathy	G672.00
Seen in hypertension clinic	9N03.00
Systolic hypertension	G202.00
Hypertensive renal disease	G22..00
Hypertensive retinopathy	F421300
Hypertensive disease NOS	G2z..00
Secondary hypertension	G24..00
Borderline hyperten:yearly obs	6624
BP - hypertensive disease	G2...11
Cardiomegaly - hypertensive	G21z011
Essential hypertension NOS	G20z.00
Patient on maximal tolerated antihypertensive therapy	8BL0.00
Hypertension treatm.stopped	662H.00
Hypertensive treatm.changed	662G.00
Hypertensive renal disease NOS	G22z.00
Malignant essential hypertension	G200.00
Secondary hypertension NOS	G24z.00
Hypertensive heart disease NOS	G21zz00
Hypertensive heart disease	G21..00
Good hypertension control	6627
Antihypertensive therapy	8B26.00
Hypertension six month review	662c.00
Moderate hypertension control	662b.00
Other specified hypertensive disease	G2y..00
Hypertension annual review	662d.00
Hypertension resolved	212K.00
Adverse reaction to antihypertensives NOS	TJC7z00
Adverse reaction to other antihypertensives	TJC7.00
Hypertension treatm. started	662F.00
Hypertensive heart&renal dis wth (congestive) heart failure	G232.00
Hypertension treatment refused	8I3N.00
Secondary benign renovascular hypertension	G241000
Fetus or neonate affected by maternal hypertensive disease	Q000.00
Poor hypertension control	6628
Seen in hypertension clinic	9N1y200

Hypertensive heart and renal disease with renal failure	G233.00
Renal hypertension	G22z.11
[X] Adverse reaction to other antihypertensives	U60C511
Hypertension:follow-up default	6629
Hypertension secondary to drug	G24z100
Secondary renovascular hypertension NOS	G24z000
Hypertensive heart disease NOS	G21z.00
Secondary malignant hypertension	G240.00
Hypertensive crisis	G672.11
Hypertensive renal disease with renal failure	G222.00
Hypertension induced by oral contraceptive pill	6146200
Hypertension secondary to endocrine disorders	G244.00
Blind hypertensive eye	F404200
Malignant hypertensive renal disease	G220.00
Secondary hypertension NOS	G24zz00
Benign hypertensive renal disease	G221.00
[X] Adverse reaction to antihypertensives NOS	U60C51A
Pre-exist hypertension compl preg childbirth and puerperium	L128.00
Malignant hypertensive heart disease	G210.00
Secondary benign hypertension NOS	G241z00
Benign hypertensive heart disease with CCF	G211100
Benign hypertensive heart disease	G211.00
Secondary benign hypertension	G241.00
Hyperten heart&renal dis+both(congestv)heart and renal fail	G234.00
Secondary malignant renovascular hypertension	G240000
Pre-exist hyperten heart dis compl preg childbth+puerperium	L128000
Hypertensive heart disease NOS without CCF	G21z000
Benign hypertensive heart disease without CCF	G211000
Other pre-existing hypertension in preg/childb/puerp NOS	L122z00
Hypertensive heart disease NOS with CCF	G21z100
Benign hypertensive heart and renal disease	G231.00
[X]Oth antihyperten drug caus advers eff in therap use	NEC
Hypertensive heart and renal disease	G23..00

Other pre-existing hypertension in preg/childbirth/puerp	L122.00
Hypertens.monitor deleted	9OI9.00
Malignant hypertensive heart and renal disease	G230.00
Hypertensive heart and renal disease NOS	G23z.00
[X]Hypertensive diseases	Gyu2.00
Other pre-existing hypertension in preg/childb/puerp - deliv	L122100
Other hypertensive agent poisoning	SLC6.00
Malignant hypertensive heart disease with CCF	G210100
Secondary malignant hypertension NOS	G240z00
Other pre-existing hypertension in preg/childb/puerp unspec	L122000
Diastolic hypertension	G203.00
High cost hypertension drugs	7Q01.00
Pre-eclampsia or eclampsia + pre-existing hypertension NOS	L127z00
Malignant hypertensive heart disease without CCF	G210000
Trial withdrawal of antihypertensive therapy	662r.00
Other pre-exist hypertension in preg/childb/puerp-not deliv	L122300
[X]Hypertension secondary to other renal disorders	Gyu2100
Trial reduction of antihypertensive therapy	662q.00
Other specified high cost hypertension drugs	7Q01y00
Hypertension 9 month review	662P000
[X]Other secondary hypertension	Gyu2000
Malignant hypertensive heart disease NOS	G210z00
Stage 2 hypertension (NICE - Nat Ins for Hth Clin Excl 2011)	G28..00
Stage 1 hypertension	G25..11
Stage 1 hypertension (NICE - Nat Ins for Hth Clin Excl 2011)	G25..00
Hypertension resistant to drug therapy	G27..00
Severe hypertension	G26..11
Benign hypertensive heart disease NOS	G211z00
Severe hypertension (Nat Inst for Health Clinical Ex 2011)	G26..00
Trial withdrawal of antihypertensive therapy declined	8IA5.00
Primary hypertension	G20..12

Stage 1 hyperten (NICE 2011) without evidnce end organ damge	G250.00
Stage 1 hyperten (NICE 2011) with evidnce end organ damge	G251.00
Other pre-exist hypertension in preg/childb/puerp + p/n comp	L122400
DESCRIPTION	READ_CODE
Thyrotoxicosis	C02..00
Hyperthyroidism	C02..11
Thyrotoxic exophthalmos	F4G2000
Thyrotoxicosis without mention of goitre	cause with crisis
Graves' disease	C020.12
H/O: hyperthyroidism	1431
Toxic goitre	C02..12
Toxic multinodular goitre	C022.00
Thyrotoxicosis without mention of goitre or other cause	C02z.00
Toxic nodular goitre unspecified	C023.00
Thyrotoxic heart disease	G557500
Toxic diffuse goitre	C020.00
Hyperthyroidism resolved	212P.00
Thyrotoxicosis NOS	C02zz00
Thyrotoxicosis without mention of goitre or cause no crisis	C02z000
Toxic diffuse goitre with no crisis	C020000
Toxic uninodular goitre with no crisis	C021000
Thyrotoxicosis of other specified origin NOS	C02yz00
Thyrotoxicosis of other specified origin	C02y.00
Toxic multinodular goitre with no crisis	C022000
Toxic diffuse goitre NOS	C020z00
Toxic nodular goitre NOS	C023z00
Thyrotoxicosis from ectopic thyroid nodule	C024.00
Thyrotoxicosis of other specified origin with no crisis	C02y000
Toxic uninodular goitre	C021.00
Toxic multinodular goitre NOS	C022z00
Thyrotoxicosis from ectopic thyroid nodule NOS	C024z00
Toxic diffuse goitre with crisis	C020100
Toxic uninodular goitre NOS	C021z00
Thyrotoxicosis from ectopic thyroid nodule with no crisis	C024000
Toxic nodular goitre unspecified with no crisis	C023000
Toxic nodular goitre unspecified with crisis	C023100

Thyrotoxicosis of other specified origin with crisis	C02y100
Toxic multinodular goitre with crisis	C022100
DESCRIPTION	READ_CODE
Crohn's disease	J40..11
Crohn's colitis	J401z11
Crohn's disease of the small bowel NOS	J400z00
Regional enteritis - Crohn's disease	J40..00
Juvenile arthritis in Crohn's disease	N045300
Arthropathy in Crohn's disease	N031100
Crohn's disease of the large bowel NOS	J401z00
Crohn's disease of the terminal ileum	J400200
Orofacial Crohn's disease	J08z900
Exacerbation of Crohn's disease of small intestine	J400500
Exacerbation of Crohn's disease of large intestine	J401200
Crohn's disease of the ileum NOS	J400400
Crohn's disease NOS	J40z.11
Crohn's disease of the ileum unspecified	J400300
[X]Other Crohn's disease	Jyu4000
DESCRIPTION	READ_CODE
Ulcerative colitis	J410100
Ulcerative colitis and/or proctitis	J41..12
H/O: ulcerative colitis	14C4.11
Ulcerative proctocolitis	J410.00
Ulcerative proctitis	J410300
Arthropathy in ulcerative colitis	N031000
Exacerbation of ulcerative colitis	J410400
Ulcerative rectosigmoiditis	J410200
Ulcerative (chronic) enterocolitis	J411.00
Ulcerative proctocolitis NOS	J410z00
[X]Other ulcerative colitis	Jyu4100
Juvenile arthritis in ulcerative colitis	N045400
Ulcerative pancolitis	J413.00
DESCRIPTION	READ_CODE
Discoid lupus erythematosus	M154100
Lupus erythematosus	M154.00
Lupus erythematosus NOS	M154z00
Systemic lupus erythematosus	N000.00
Systemic lupus erythematosus with pericarditis	N000400
Disseminated lupus erythematosus	N000000
Subacute cutaneous lupus erythematosus	M154700

Systemic lupus erythematosus with organ or sys involv	N000300
Lung disease with systemic lupus erythematosus	H57y400
Lupus erythematosus chronicus	M154000
Drug-induced systemic lupus erythematosus	N000200
Lupus erythematosus migrans	M154200
Systemic lupus erythematosus NOS	N000z00
Polyneuropathy in disseminated lupus erythematosus	F371000
Lupus erythematosus tumidus	M154500
Systemic lupus erythematosus disease activity index	ZRq9.00
Lupus erythematosus profundus	M154400
Nephrotic syndrome in systemic lupus erythematosus	K01x400
Systemic lupus activity measure	ZRq8.00
[X]Other forms of systemic lupus erythematosus	Nyu4300
Lupus erythematosus unguium mutilans	M154600
Lupus erythematosus nodularis	M154300
Eyelid discoid lupus erythematosus	F4D3300
[X]Other local lupus erythematosus	Myu7800
SLAM - Systemic lupus activity measure	ZRq8.11
Myopathy due to disseminated lupus erythematosus	F396100
DESCRIPTION	READ_CODE
Insulin dependent diabetes mellitus	C100011
Type 1 diabetes mellitus	C10E.00
Insulin dependent diabetes mellitus	C108.00
Insulin dependent diabetes mellitus with retinopathy	C108700
Insulin dependent diabetes mellitus - poor control	C108800
Type 1 diabetes mellitus with nephropathy	C10ED00
Type 1 diabetes mellitus with ketoacidosis	C10EM00
Type I diabetes mellitus	C10E.11
Type I diabetes mellitus with diabetic cataract	C108F11
Type 1 diabetes mellitus	C108.12
Type 1 diabetes mellitus with neuropathic arthropathy	C108J12
Type 1 diabetes mellitus with retinopathy	C10E700
IDDM-Insulin dependent diabetes mellitus	C108.11

Type 1 diabetes mellitus with arthropathy	C10EH00
Type 1 diabetes mellitus with ulcer	C10E500
Type 1 diabetes mellitus with renal complications	C108012
Type 1 diabetes mellitus with exudative maculopathy	C10EP00
Type I diabetes mellitus	C108.13
Insulin dependent diabetes mellitus with mononeuropathy	C108B00
Unstable insulin dependent diabetes mellitus	C108400
Type 1 diabetes mellitus with persistent microalbuminuria	C10EL00
Type 1 diabetes mellitus with persistent proteinuria	C10EK00
Insulin dependent diabetes maturity onset	C108900
Type 1 diabetes mellitus - poor control	C10E800
Type I diabetes mellitus with retinopathy	C108711
Type 1 diabetes mellitus with hypoglycaemic coma	C10EE00
Insulin dependent diab mell with neuropathic arthropathy	C108J00
Type 1 diabetes mellitus maturity onset	C10E900
Type 1 diabetes mellitus with ketoacidotic coma	C10EN00
Type 1 diabetes mellitus with retinopathy	C108712
Insulin dependent diabetes mellitus with polyneuropathy	C108C00
Type I diabetes mellitus with hypoglycaemic coma	C108E11
Type 1 diabetes mellitus with neurological complications	C10E200
Unstable type 1 diabetes mellitus	C10E400
Insulin dependent diabetes mellitus with diabetic cataract	C108F00
Insulin dependent diabetes mellitus with hypoglycaemic coma	C108E00
Insulin dependent diabetes mellitus with ulcer	C108500
Insulin dependent diabetes mellitus with multiple complicat	C10E312
Type 1 diabetes mellitus - poor control	C108812
Type 1 diabetes mellitus with polyneuropathy	C10EC00
Type I diabetes mellitus - poor control	C108811
Type 1 diabetes mellitus with renal	C10E000

complications	
Type 1 diabetes mellitus with ophthalmic complications	C10E100
Type 1 diabetes mellitus with multiple complications	C10E300
Type I diabetes mellitus with neurological complications	C108211
Type 1 diabetes mellitus with diabetic cataract	C10EF00
Unstable type I diabetes mellitus	C10E411
Insulin dependent diabetes mellitus	C10E.12
Type I diabetes mellitus with ulcer	C108511
Insulin dependent diabetes mellitus with multiple complicatn	C108300
Type 1 diabetes mellitus with neuropathic arthropathy	C10EJ00
Unstable insulin dependent diabetes mellitus	C10E412
Type 1 diabetes mellitus with gastroparesis	C10EQ00
Insulin dependent diabetes mellitus with nephropathy	C108D00
Unstable type I diabetes mellitus	C108411
Type I diabetes mellitus with neuropathic arthropathy	C108J11
Insulin dependent diabetes mellitus with gangrene	C108600
Type I diabetes mellitus with renal complications	C108011
Type 1 diabetes mellitus with neurological complications	C108212
Type I diabetes mellitus with ketoacidosis	C10EM11
Type I diabetes mellitus with arthropathy	C108H11
Type I diabetes mellitus without complication	C10EA11
Type I diabetes mellitus maturity onset	C108911
Insulin dependent diab mell with peripheral angiopathy	C108G00
Insulin dependent diabetes mellitus with arthropathy	C108H00
Type I diabetes mellitus with ketoacidotic coma	C10EN11
Type I diabetes mellitus with nephropathy	C108D11
Type 1 diabetes mellitus with mononeuropathy	C10EB00
Type 1 diabetes mellitus with ulcer	C108512
Dietary advice for type I diabetes	ZC2C900
Type 1 diabetes mellitus without	C10EA00

complication	
Type 1 diabetes mellitus with gangrene	C10E600
Type 1 diabetes mellitus with hypoglycaemic coma	C108E12
Insulin dependent diabetes mellitus - poor control	C10E812
Type I diabetes mellitus with multiple complications	C10E311
Type I diabetes mellitus with polyneuropathy	C10EC11
Type 1 diabetes mellitus with peripheral angiopathy	C10EG00
Insulin dependent diabetes mellitus with retinopathy	C10E712
Type I diabetes mellitus with ulcer	C10E511
Type I diabetes mellitus with retinopathy	C10E711
Type I diabetes mellitus without complication	C108A11
Type I diabetes mellitus maturity onset	C10E911
Type 1 diabetes mellitus maturity onset	C108912
Unstable type 1 diabetes mellitus	C108412
Insulin dependent diabetes maturity onset	C10E912
Type I diabetes mellitus with exudative maculopathy	C10EP11
Insulin dependent diabetes mellitus with ulcer	C10E512
Type I diabetes mellitus with mononeuropathy	C108B11
Type I diabetes mellitus with ophthalmic complications	C10E111
Insulin dependent diabetes mellitus with hypoglycaemic coma	C10EE12
Insulin dependent diabetes mellitus with diabetic cataract	C10EF12
Insulin dependent diabetes mellitus with polyneuropathy	C10EC12
Type I diabetes mellitus with gangrene	C10E611
Insulin dependent diabetes mellitus with nephropathy	C10ED12
Type I diabetes mellitus with persistent microalbuminuria	C10EL11
Type 1 diabetes mellitus with ophthalmic complications	C108112
Type 1 diabetic dietary review	66At011
Type I diabetes mellitus - poor control	C10E811
Type I diabetes mellitus with multiple complications	C108311

Type I diabetes mellitus in remission	C10P000
Type I diabetes mellitus with gastroparesis	C10EQ11
Insulin dependent diabetes mellitus with gangrene	C10E612
Type 1 diabetes mellitus in remission	C10P011
Type I diabetes mellitus with renal complications	C10E011
Type 1 diabetes mellitus with diabetic cataract	C108F12
Type 1 diabetes mellitus without complication	C108A12
Type 1 diabetes mellitus with nephropathy	C108D12
Type I diabetes mellitus with nephropathy	C10ED11
Type I diabetes mellitus with polyneuropathy	C108C11
Type 1 diabetes mellitus with gangrene	C108612
DESCRIPTION	READ_CODE
Non-insulin dependent diabetes mellitus	C100112
Type 2 diabetes mellitus	C10F.00
Insulin treated Type 2 diabetes mellitus	C10FJ00
Diabetic on oral treatment	66A4.00
Non-insulin dependent diabetes mellitus	C109.00
NIDDM - Non-insulin dependent diabetes mellitus	C109.11
Diabetic on diet only	66A3.00
Non-insulin dependent diabetes mellitus - poor control	C109700
Type 2 diabetes mellitus with nephropathy	C10FC00
Type 2 diabetes mellitus with gangrene	C10F500
Non-insulin-dependent diabetes mellitus with retinopathy	C109600
Type 2 diabetes mellitus	C109.12
Type II diabetes mellitus with arthropathy	C109G11
Type 2 diabetes mellitus with renal complications	C109012
Type II diabetes mellitus	C109.13
Insulin treated Type II diabetes mellitus	C109J12
Insulin treated Type 2 diabetes mellitus	C109J00
Type 2 diabetes mellitus with persistent microalbuminuria	C10FM00

Type 2 diabetes mellitus with polyneuropathy	C10FB00
Type 2 diabetes mellitus with retinopathy	C10F600
Type 2 diabetes mellitus with renal complications	C10F000
Type II diabetes mellitus	C10F.11
Type II diabetes mellitus - poor control	C109711
Non-insulin dependent diabetes mellitus with arthropathy	C109G00
Type 2 diabetes mellitus with nephropathy	C109C12
Dietary advice for type II diabetes	ZC2CA00
Type 2 diabetes mellitus with exudative maculopathy	C10FQ00
Type 2 diabetes mellitus - poor control	C10F700
Type 2 diabetes mellitus with persistent proteinuria	C10FL00
Non-insulin-dependent diabetes mellitus without complication	C109900
Type 2 diabetes mellitus with ketoacidosis	C10FN00
Type 2 diabetes mellitus with neurological complications	C10F200
Hyperosmolar non-ketotic state in type 2 diabetes mellitus	C10FK00
Non-insulin dependent diabetes mellitus with ulcer	C109400
Type 2 diabetes mellitus with neuropathic arthropathy	C10FH00
Hyperosmolar non-ketotic state in type 2 diabetes mellitus	C109K00
Insulin treated non-insulin dependent diabetes mellitus	C109J11
Type 2 diabetes mellitus with peripheral angiopathy	C10FF00
Non-insulin dependent diabetes mellitus with gangrene	C109500
Non-insulin dependent d m with neuropathic arthropathy	C109H00
Type 2 diabetes mellitus with retinopathy	C109612
Type II diabetes mellitus with multiple complications	C10F311
Non-insulin dependent diabetes mellitus with hypoglyca coma	C109D00
Type 2 diabetes mellitus with diabetic cataract	C109E12
Type 2 diabetes mellitus with diabetic cataract	C10FE00
Non-insulin dependent diabetes	C109B00

mellitus with polyneuropathy	
Type 2 diabetes mellitus - poor control	C109712
Type 2 diabetes mellitus with neurological complications	C109212
Type 2 diabetes mellitus with gangrene	C109512
Type 2 diabetes mellitus with hypoglycaemic coma	C10FD00
Type II diabetes mellitus - poor control	C10F711
Type 2 diabetes mellitus with ophthalmic complications	C10F100
Type II diabetes mellitus with polyneuropathy	C109B11
Type II diabetes mellitus with neuropathic arthropathy	C109H11
Type 2 diabetes mellitus without complication	C10F900
Type II diabetes mellitus with diabetic cataract	C109E11
Type 2 diabetes mellitus with ulcer	C10F400
Type II diabetes mellitus with retinopathy	C10F611
Type 2 diabetes mellitus with arthropathy	C109G12
Type II diabetes mellitus with renal complications	C109011
Non-insulin-dependent diabetes mellitus with ophthalm comps	C109100
Type II diabetes mellitus with polyneuropathy	C10FB11
Pre-existing diabetes mellitus	non-insulin-dependent
Type II diabetes mellitus with mononeuropathy	C109A11
Type 2 diabetes mellitus with ketoacidotic coma	C10FP00
Non-insulin-dependent diabetes mellitus with renal comps	C109000
Type II diabetes mellitus without complication	C10F911
Non-insulin-dependent d m with peripheral angiopath	C109F00
Type II diabetes mellitus with peripheral angiopathy	C109F11
Type II diabetes mellitus with ulcer	C109411
Non-insulin-dependent diabetes mellitus with neuro comps	C109200
Type II diabetes mellitus with hypoglycaemic coma	C109D11
NIDDM with peripheral circulatory disorder	C107400

Type II diabetes mellitus with renal complications	C10F011
Type II diabetes mellitus with retinopathy	C109611
Type 2 diabetes mellitus with arthropathy	C10FG00
Non-insulin dependent diabetes mellitus with nephropathy	C109C00
Type II diabetes mellitus with ophthalmic complications	C109111
Type 2 diabetes mellitus with peripheral angiopathy	C109F12
Type II diabetes mellitus with persistent proteinuria	C10FL11
Type 2 diabetes mellitus with hypoglycaemic coma	C109D12
Type II diabetes mellitus with gangrene	C109511
Non-insulin-dependent diabetes mellitus with multiple comps	C109300
Type 2 diabetes mellitus with mononeuropathy	C10FA00
Type 2 diabetes mellitus with gastroparesis	C10FR00
Type II diabetes mellitus with nephropathy	C109C11
Insulin treated Type II diabetes mellitus	C10FJ11
Type 2 diabetes mellitus with multiple complications	C10F300
Type 2 diabetes mellitus with ulcer	C109412
Type 2 diabetes mellitus with neuropathic arthropathy	C109H12
Type II diabetes mellitus with neurological complications	C109211
Non-insulin depend diabetes mellitus with diabetic cataract	C109E00
Type 2 diabetes mellitus with ophthalmic complications	C109112
Non-insulin dependent diabetes mellitus with mononeuropathy	C109A00
Type II diabetes mellitus with persistent microalbuminuria	C10FM11
Type II diabetes mellitus with ulcer	C10F411
Type II diabetes mellitus with diabetic cataract	C10FE11
Type II diabetes mellitus with mononeuropathy	C10FA11
Type II diabetes mellitus with neurological complications	C10F211
Type II diabetes mellitus with hypoglycaemic coma	C10FD11
Type II diabetes mellitus with	C10F111

ophthalmic complications	
Type II diabetes mellitus with nephropathy	C10FC11
Type II diabetes mellitus with arthropathy	C10FG11
Type II diabetes mellitus with gangrene	C10F511
Type II diabetes mellitus with peripheral angiopathy	C10FF11
Type 2 diabetes mellitus without complication	C109912
Type II diabetes mellitus with ketoacidotic coma	C10FP11
Type II diabetes mellitus with ketoacidosis	C10FN11
Hyperosmolar non-ketotic state in type II diabetes mellitus	C10FK11
Type II diabetes mellitus in remission	C10P100
Type 2 diabetes mellitus with multiple complications	C109312
Type II diabetes mellitus without complication	C109911
Type II diabetes mellitus with neuropathic arthropathy	C10FH11
Conversion to non-insulin injectable medication	66AH300
Type 2 diabetes mellitus with polyneuropathy	C109B12
Diabetic on non-insulin injectable medication	66o2.00
Diabetic on oral treatment and glucagon-like peptide 1	66o5.00
Type 2 diabetes mellitus in remission	C10P111
Type II diabetes mellitus with exudative maculopathy	C10FQ11
Type II diabetes mellitus with multiple complications	C109311
Type II diabetes mellitus with gastroparesis	C10FR11
Focal epilepsy	F255011
Status epilepticus	unspecified
[X]Epileptic psychosis NOS	Eu05y11
Epilepsy monitoring	667..00
Absence seizure	2828
Tonic-clonic epilepsy	F251500
Jacksonian	focal or motor epilepsy
Epilepsy NOS	F25z.00
Petit mal status	F252.00
Locl-rlt(foc)(part)idiop epilep&epilptic syn seiz locl onset	F25y200

Other forms of epilepsy NOS	F25yz00
Generalised nonconvulsive epilepsy	F250.00
Complex partial epileptic seizure	F254500
Epilepsy control poor	667D.00
Juvenile absence epilepsy	F250400
Epileptic seizures - clonic	F251200
Benign Rolandic epilepsy	F25y400
Juvenile myoclonic epilepsy	F25A.00
Epilepsy control good	667C.00
Epilepsy does not limit activities	667L.00
Epilepsy treatment stopped	667A.00
Epilepsy confirmed	1O30.00
Tonic-clonic epilepsy	F251011
Salaam attacks	F256100
Psychomotor epilepsy	F254100
Epileptic seizures - atonic	F250200
Complex partial status epilepticus	F25y300
Partial epilepsy without impairment of consciousness	F255.00
Generalised convulsive epilepsy	F251.00
Epilepsy treatment changed	6678
Epilepsy limits activities	667K.00
Epilepsy restricts employment	667G.00
Partial epilepsy without impairment of consciousness OS	F255y00
Partial epilepsy without impairment of consciousness NOS	F255z00
Alcohol-induced epilepsy	F25B.00
Photosensitive epilepsy	F25F.00
Drug-induced epilepsy	F25C.00
Epileptic seizures - akinetic	F250300
[X]Schizophrenia-like psychosis in epilepsy	Eu05212
Partial epilepsy with impairment of consciousness NOS	F254z00
Partial epilepsy with impairment of consciousness	F254.00
Epileptic automatism	F254400
Epilepsy treatment started	6679
Lennox-Gastaut syndrome	F250500
Psychosensory epilepsy	F254200
Epilepsy monitoring NOS	667Z.00
Somatosensory epilepsy	F255200
Progressive myoclonic epilepsy	F132100
Neonatal myoclonic epilepsy	F251100
Early infant epileptic encephalopathy wth suppression bursts	F259.00
Other forms of epilepsy	F25y.00

Transient epileptic amnesia	1B1W.00
West syndrome	F256.12
Simple partial epileptic seizure	F255600
Generalised convulsive epilepsy NOS	F251z00
Epilepsy impairs education	667J.00
[X]Acquired aphasia with epilepsy [Landau - Kleffner]	Eu80300
Generalised nonconvulsive epilepsy NOS	F250z00
Other specified generalised convulsive epilepsy	F251y00
Emergency epilepsy treatment since last appointment	667W.00
Sensory induced epilepsy	F255100
[X]Limbic epilepsy personality	Eu06013
Infantile spasms NOS	F256z00
Otohara syndrome	F251111
Acquired epileptic aphasia	ZS82.00
Epilepsy associated problems	6674
Epilepsy prevents employment	667H.00
Ohtahara syndrome	F259.11
Gelastic epilepsy	F25y100
Cursive (running) epilepsy	F25y000
Limbic system epilepsy	F254300
Visual reflex epilepsy	F255400
Menstrual epilepsy	F25D.00
[X]Other status epilepticus	Fyu5200
Other specified generalised nonconvulsive epilepsy	F250y00
Stress-induced epilepsy	F25E.00
Motor epilepsy	F255012
Lightning spasms	F256.11
Unilateral epilepsy	F255500
[X]Other epilepsy	Fyu5100
Kojevnikov's epilepsy	F257.00
Visceral reflex epilepsy	F255300
Panayiotopoulos syndrome	F25y500
Partial epilepsy with autonomic symptoms	F255311
Pykno-epilepsy	F250100
[X]Other generalized epilepsy and epileptic syndromes	Fyu5000
Dravet syndrome	F25G.11
Severe myoclonic epilepsy in infancy	F25G.00
At risk of sudden unexpected death in epilepsy	14On.00

Drug codes

DESCRIPTION	BNF1
Sumatriptan 50mg tablets Sumatriptan succinate 50mg Tablet Oral	04070401
Sumatriptan 100mg tablets Sumatriptan succinate 100mg Tablet Oral	04070401
Migraleve tablets (McNeil Products Ltd) Not applicable Route of administration not applicable	04070200
Migraleve Pink tablets (McNeil Products Ltd) Buclizine hydrochloride/Paracetamol/Codeine phosphate 6.25mg + 500mg + 8mg Tablet Oral	04070200
Rizatriptan 10mg oral lyophilisates sugar free Rizatriptan benzoate 10mg Oral lyophilisate Oral	04070401
Imigran 50mg tablets (GlaxoSmithKline UK Ltd) Sumatriptan succinate 50mg Tablet Oral	04070401
Zolmitriptan 5mg/0.1ml nasal spray unit dose Zolmitriptan 50mg/1ml Spray Nasal	04070401
Rizatriptan 10mg orodispersible tablets sugar free Rizatriptan benzoate 10mg Orodispersible tablet Oral	04070401
Migard 2.5mg tablets (A. Menarini Farmaceutica Internazionale SRL) Frovatriptan succinate monohydrate 2.5mg Tablet Oral	04070401
Naramig 2.5mg tablets (DE Pharmaceuticals) Naratriptan hydrochloride 2.5mg Tablet Oral	04070401
Imigran 10mg nasal spray (DE Pharmaceuticals) Sumatriptan 100mg/1ml Spray Nasal	04070401
Sumatriptan 50mg tablets (Mylan) Sumatriptan succinate 50mg Tablet Oral	04070401
Rizatriptan 10mg orodispersible tablets sugar free (Actavis UK Ltd) Rizatriptan benzoate 10mg Orodispersible tablet Oral	04070401
Imigran Radis 50mg tablets (GlaxoSmithKline UK Ltd) Sumatriptan succinate 50mg Tablet Oral	04070401
Sumatriptan 50mg tablets (Alliance Healthcare (Distribution) Ltd) Sumatriptan succinate 50mg Tablet Oral	04070401
Zolmitriptan 5mg orodispersible tablets sugar free Zolmitriptan 5mg Orodispersible tablet Oral	04070401
HealthAid 5-HTP HydroxyTryptoPhan 50mg tablets (HealthAid Ltd) Oxitriptan 50mg Modified-release tablet Oral	09040251
Zomig Rapimelt 2.5mg orodispersible tablets (Grunenthal Ltd) Zolmitriptan 2.5mg Orodispersible tablet Oral	04070401
Sumatriptan 50mg tablets (Accord Healthcare Ltd) Sumatriptan succinate 50mg Tablet Oral	04070401
Zolmitriptan 2.5mg tablets (Actavis UK Ltd) Zolmitriptan 2.5mg Tablet Oral	04070401
Maxalt Melt 10mg oral lyophilisates (Merck Sharp & Dohme Ltd) Rizatriptan benzoate 10mg Oral lyophilisate Oral	04070401
Generic Migraleve Pink tablets Buclizine hydrochloride/Paracetamol/Codeine phosphate 6.25mg + 500mg + 8mg Tablet Oral	04070200
Sumatriptan 50mg tablets (Pfizer Ltd) Sumatriptan succinate 50mg Tablet Oral	04070401
Naratriptan 2.5mg tablets (A A H Pharmaceuticals Ltd) Naratriptan hydrochloride 2.5mg Tablet Oral	04070401
Zolmitriptan 2.5mg tablets Zolmitriptan 2.5mg Tablet Oral	04070401
Imigran 100mg tablets (GlaxoSmithKline UK Ltd) Sumatriptan succinate 100mg Tablet Oral	04070401
Zolmitriptan 2.5mg orodispersible tablets sugar free (Teva UK Ltd) Zolmitriptan 2.5mg Orodispersible tablet Oral	04070401
Sumatriptan 50mg tablets (Teva UK Ltd) Sumatriptan succinate 50mg Tablet Oral	04070401
Almotriptan 12.5mg tablets Almotriptan hydrogen malate 12.5mg Tablet Oral	04070401

Sumatriptan 50mg tablets (Dexcel-Pharma Ltd) Sumatriptan succinate 50mg Tablet Oral	04070401
Sumatriptan 100mg tablets (A A H Pharmaceuticals Ltd) Sumatriptan succinate 100mg Tablet Oral	04070401
Sumatriptan 50mg tablets (Actavis UK Ltd) Sumatriptan succinate 50mg Tablet Oral	04070401
Sumatriptan 50mg tablets (Almus Pharmaceuticals Ltd) Sumatriptan succinate 50mg Tablet Oral	04070401
Sumatriptan 100mg tablets (Almus Pharmaceuticals Ltd) Sumatriptan succinate 100mg Tablet Oral	04070401
Zomig 5mg/0.1ml nasal spray 0.1ml unit dose (Waymade Healthcare Plc) Zolmitriptan 50mg/1ml Spray Nasal	04070401
Naramig 2.5mg tablets (GlaxoSmithKline UK Ltd) Naratriptan hydrochloride 2.5mg Tablet Oral	04070401
Sumatriptan 50mg tablets (Dr Reddy's Laboratories (UK) Ltd) Sumatriptan succinate 50mg Tablet Oral	04070401
Rizatriptan 5mg tablets (A A H Pharmaceuticals Ltd) Rizatriptan benzoate 5mg Tablet Oral	04070401
Sumatriptan 100mg tablets (Arrow Generics Ltd) Sumatriptan succinate 100mg Tablet Oral	04070401
Zolmitriptan 5mg orodispersible tablets sugar free (Zentiva) Zolmitriptan 5mg Orodispersible tablet Oral	04070401
Maxalt Melt 10mg oral lyophilisates (Mawdsley-Brooks & Company Ltd) Rizatriptan benzoate 10mg Oral lyophilisate Oral	04070401
Eletriptan 20mg tablets Eletriptan hydrobromide 20mg Tablet Oral	04070401
Zolmitriptan 2.5mg tablets (Teva UK Ltd) Zolmitriptan 2.5mg Tablet Oral	04070401
Rizatriptan 10mg orodispersible tablets sugar free (Alliance Healthcare (Distribution) Ltd) Rizatriptan benzoate 10mg Orodispersible tablet Oral	04070401
Sumatriptan 50mg tablets (Sandoz Ltd) Sumatriptan succinate 50mg Tablet Oral	04070401
Imigran 10mg nasal spray (Lexon (UK) Ltd) Sumatriptan 100mg/1ml Spray Nasal	04070401
Maxalt 10mg tablets (Merck Sharp & Dohme Ltd) Rizatriptan benzoate 10mg Tablet Oral	04070401
Sumatriptan 50mg tablets (Milpharm Ltd) Sumatriptan succinate 50mg Tablet Oral	04070401
Zolmitriptan 2.5mg orodispersible tablets sugar free (Alliance Healthcare (Distribution) Ltd) Zolmitriptan 2.5mg Orodispersible tablet Oral	04070401
Rizatriptan 10mg tablets (A A H Pharmaceuticals Ltd) Rizatriptan benzoate 10mg Tablet Oral	04070401
Imigran 50mg tablets (Lexon (UK) Ltd) Sumatriptan succinate 50mg Tablet Oral	04070401
Sumatriptan 50mg tablets (Arrow Generics Ltd) Sumatriptan succinate 50mg Tablet Oral	04070401
Frovatriptan 2.5mg tablets Frovatriptan succinate monohydrate 2.5mg Tablet Oral	04070401
Relpax 20mg tablets (Upjohn UK Ltd) Eletriptan hydrobromide 20mg Tablet Oral	04070401
Zolmitriptan 5mg tablets Zolmitriptan 5mg Tablet Oral	04070401
Migraitan 50mg tablets (Bristol Laboratories Ltd) Sumatriptan succinate 50mg Tablet Oral	04070401
Sumatriptan 50mg tablets (Bristol Laboratories Ltd) Sumatriptan succinate 50mg Tablet Oral	04070401
Migraleve Yellow tablets (McNeil Products Ltd) Paracetamol/Codeine phosphate 500mg + 8mg Tablet Oral	04070100
Lamberts 5-HTP 100mg tablets (Lamberts Healthcare Ltd) Oxitriptan 100mg Tablet Oral	09040251
Sumatriptan 100mg tablets (Milpharm Ltd) Sumatriptan succinate 100mg Tablet Oral	04070401

Imigran Radis 50mg tablets (Waymade Healthcare Plc) Sumatriptan succinate 50mg Tablet Oral	04070401
Imigran 10mg nasal spray (GlaxoSmithKline UK Ltd) Sumatriptan 100mg/1ml Spray Nasal	04070401
Sumatriptan 100mg tablets (Dr Reddy's Laboratories (UK) Ltd) Sumatriptan succinate 100mg Tablet Oral	04070401
Zolmitriptan 2.5mg orodispersible tablets sugar free Zolmitriptan 2.5mg Orodispersible tablet Oral	04070401
Zomig 5mg/0.1ml nasal spray 0.1ml unit dose (Grunenthal Ltd) Zolmitriptan 50mg/1ml Spray Nasal	04070401
Maxalt 10mg tablets (Waymade Healthcare Plc) Rizatriptan benzoate 10mg Tablet Oral	04070401
Zomig 2.5mg tablets (Grunenthal Ltd) Zolmitriptan 2.5mg Tablet Oral	04070401
Rizatriptan 5mg tablets Rizatriptan benzoate 5mg Tablet Oral	04070401
Imigran 50mg tablets (DE Pharmaceuticals) Sumatriptan succinate 50mg Tablet Oral	04070401
Naratriptan 2.5mg tablets Naratriptan hydrochloride 2.5mg Tablet Oral	04070401
Generic Migraleve tablets Not applicable Route of administration not applicable	04070200
Rizatriptan 10mg orodispersible tablets sugar free (A A H Pharmaceuticals Ltd) Rizatriptan benzoate 10mg Orodispersible tablet Oral	04070401
Sumatriptan 50mg tablets (A A H Pharmaceuticals Ltd) Sumatriptan succinate 50mg Tablet Oral	04070401
Migraleve - 1 Tablet (Pfizer Consumer Healthcare Ltd) Buclizine Hydrochloride/Codeine Phosphate/Paracetamol Tablet Oral	03040102
Imigran Recovery 50mg tablets (Forest Laboratories UK Ltd) Sumatriptan succinate 50mg Tablet Oral	04070401
Zolmitriptan 5mg orodispersible tablets sugar free (Teva UK Ltd) Zolmitriptan 5mg Orodispersible tablet Oral	04070401
Maxalt 10mg tablets (DE Pharmaceuticals) Rizatriptan benzoate 10mg Tablet Oral	04070401
Migraleve Ultra 50mg tablets (McNeil Products Ltd) Sumatriptan succinate 50mg Tablet Oral	04070401
Imigran Radis 100mg tablets (GlaxoSmithKline UK Ltd) Sumatriptan succinate 100mg Tablet Oral	04070401
Zomig Rapimelt 2.5mg orodispersible tablets (Mawdsley-Brooks & Company Ltd) Zolmitriptan 2.5mg Orodispersible tablet Oral	04070401
Rizatriptan 5mg orodispersible tablets sugar free Rizatriptan benzoate 5mg Orodispersible tablet Oral	04070401
Sumatriptan 100mg tablets (Waymade Healthcare Plc) Sumatriptan succinate 100mg Tablet Oral	04070401
Naratriptan 2.5mg tablets (Teva UK Ltd) Naratriptan hydrochloride 2.5mg Tablet Oral	04070401
Sumatriptan 20mg/0.1ml nasal spray unit dose Sumatriptan 200mg/1ml Spray Nasal	04070401
Zomig Rapimelt 5mg orodispersible tablets (Grunenthal Ltd) Zolmitriptan 5mg Orodispersible tablet Oral	04070401
Zolmitriptan 2.5mg orodispersible tablets sugar free (Actavis UK Ltd) Zolmitriptan 2.5mg Orodispersible tablet Oral	04070401
Eletriptan 40mg tablets Eletriptan hydrobromide 40mg Tablet Oral	04070401
Sumatriptan 10mg/0.1ml nasal spray unit dose Sumatriptan 100mg/1ml Spray Nasal	04070401
Relpax 40mg tablets (Upjohn UK Ltd) Eletriptan hydrobromide 40mg Tablet Oral	04070401
Migraleve - 2 8mg+500mg Tablet (Pfizer Consumer Healthcare Ltd)	04070100

Paracetamol/Codeine phosphate 500mg + 8mg Tablet Oral	
Maxalt 5mg tablets (Merck Sharp & Dohme Ltd) Rizatriptan benzoate 5mg Tablet Oral	04070401
Zolmitriptan 5mg tablets (Waymade Healthcare Plc) Zolmitriptan 5mg Tablet Oral	04070401
Imigran 20mg nasal spray (GlaxoSmithKline UK Ltd) Sumatriptan 200mg/1ml Spray Nasal	04070401
Rizatriptan 10mg orodispersible tablets sugar free (Mylan) Rizatriptan benzoate 10mg Orodispersible tablet Oral	04070401
Maxalt Melt 10mg oral lyophilisates (Lexon (UK) Ltd) Rizatriptan benzoate 10mg Oral lyophilisate Oral	04070401
Rizatriptan 10mg orodispersible tablets sugar free (Aspire Pharma Ltd) Rizatriptan benzoate 10mg Orodispersible tablet Oral	04070401
Maxalt Melt 10mg oral lyophilisates (DE Pharmaceuticals) Rizatriptan benzoate 10mg Oral lyophilisate Oral	04070401
Almogran 12.5mg tablets (Almirall Ltd) Almotriptan hydrogen malate 12.5mg Tablet Oral	04070401
Imigran 20mg nasal spray (Lexon (UK) Ltd) Sumatriptan 200mg/1ml Spray Nasal	04070401
Rizatriptan 10mg tablets Rizatriptan benzoate 10mg Tablet Oral	04070401
Sumatriptan 100mg tablets (Mylan)Sumatriptan succinate100mgTabletOral	04070401
Sumatriptan 100mg tablets (Dexcel-Pharma Ltd)Sumatriptan succinate100mgTabletOral	04070401
Sumatriptan 50mg tablets (DE Pharmaceuticals)Sumatriptan succinate50mgTabletOral	04070401
Zolmitriptan 5mg orodispersible tablets sugar free (A A H Pharmaceuticals Ltd)Zolmitriptan5mgOrodispersible tabletOral	04070401
Maxalt 5mg tablets (Mawdsley-Brooks & Company Ltd)Rizatriptan benzoate5mgTabletOral	04070401
Sumatriptan 100mg tablets (Pfizer Ltd)Sumatriptan succinate100mgTabletOral	04070401
Imigran 20mg nasal spray (Dowelhurst Ltd)Sumatriptan200mg/1mlSprayNasal	04070401

DESCRIPTION	BNF1
Topiramate 25mg capsules Topiramate 25mg Capsule Oral	04070402
Topiramate 50mg capsules Topiramate 50mg Capsule Oral	04070402
Topamax 25mg sprinkle capsules (Mawdsley-Brooks & Company Ltd) Topiramate 25mg Capsule Oral	04070402
Topiramate 25mg capsules (Phoenix Healthcare Distribution Ltd) Topiramate 25mg Capsule Oral	04070402
Topiramate 100mg tablets (Teva UK Ltd) Topiramate 100mg Tablet Oral	04070402
Topiramate 30mg/5ml oral solution Topiramate 6mg/1ml Oral solution Oral	04070402
Topiramate 12.5mg/5ml oral solution Topiramate 2.5mg/1ml Oral solution Oral	04070402
Topiramate 25mg capsules (A A H Pharmaceuticals Ltd) Topiramate 25mg Capsule Oral	04070402
Topiramate 25mg capsules (Alliance Healthcare (Distribution) Ltd) Topiramate 25mg Capsule Oral	04070402
Topiramate 10mg/5ml oral solution Topiramate 2mg/1ml Oral solution Oral	04070402

Topiramate 50mg capsules (A A H Pharmaceuticals Ltd) Topiramate 50mg Capsule Oral	0407040 2
Topiramate 25mg tablets (Sandoz Ltd) Topiramate 25mg Tablet Oral	0407040 2
Topiramate 25mg capsules (Actavis UK Ltd) Topiramate 25mg Capsule Oral	0407040 2
Topiramate 50mg tablets (Accord Healthcare Ltd) Topiramate 50mg Tablet Oral	0407040 2
Topiramate 30mg/5ml oral suspension Topiramate 6mg/1ml Oral suspension Oral	0407040 2
Topamax 200mg tablets (Janssen-Cilag Ltd) Topiramate 200mg Tablet Oral	0407040 2
Topiramate 100mg/5ml oral solution Topiramate 20mg/1ml Oral solution Oral	0407040 2
Topiramate 50mg capsules (Teva UK Ltd) Topiramate 50mg Capsule Oral	0407040 2
Topamax 15mg sprinkle capsules (Waymade Healthcare Plc) Topiramate 15mg Capsule Oral	0407040 2
Topiramate 50mg capsules (Phoenix Healthcare Distribution Ltd) Topiramate 50mg Capsule Oral	0407040 2
Topamax 100mg tablets (Janssen-Cilag Ltd) Topiramate 100mg Tablet Oral	0407040 2
Topiramate 50mg tablets Topiramate 50mg Tablet Oral	0407040 2
Topamax 15mg sprinkle capsules (Janssen-Cilag Ltd) Topiramate 15mg Capsule Oral	0407040 2
Topiramate 50mg tablets (A A H Pharmaceuticals Ltd) Topiramate 50mg Tablet Oral	0407040 2
Topiramate 125mg/5ml oral suspension Topiramate 25mg/1ml Oral suspension Oral	0407040 2
Topiramate 50mg tablets (Teva UK Ltd) Topiramate 50mg Tablet Oral	0407040 2
Topiramate 20mg/5ml oral suspension Topiramate 4mg/1ml Oral suspension Oral	0407040 2
Topamax 50mg sprinkle capsules (Janssen-Cilag Ltd) Topiramate 50mg Capsule Oral	0407040 2
Topiramate 25mg/5ml oral solution Topiramate 5mg/1ml Oral solution Oral	0407040 2
Topiramate 200mg tablets Topiramate 200mg Tablet Oral	0407040 2
Topiramate 12mg/5ml oral suspension Topiramate 2.4mg/1ml Oral suspension Oral	0407040 2
Topiramate 50mg/5ml oral suspension sugar free Topiramate 10mg/1ml Oral suspension Oral	0407040 2
Topiramate 100mg/5ml oral suspension sugar free Topiramate 20mg/1ml Oral suspension Oral	0407040 2
Topiramate 15mg capsules Topiramate 15mg Capsule Oral	0407040 2
Topiramate 15mg/5ml oral solution Topiramate 3mg/1ml Oral solution Oral	0407040 2
Topamax 25mg tablets (Janssen-Cilag Ltd) Topiramate 25mg Tablet Oral	0407040 2
Topiramate 25mg capsules (Sandoz Ltd) Topiramate 25mg Capsule Oral	0407040 2

Topamax 25mg sprinkle capsules (Janssen-Cilag Ltd) Topiramate 25mg Capsule Oral	04070402
Topiramate 50mg capsules (Waymade Healthcare Plc) Topiramate 50mg Capsule Oral	04070402
Topiramate 15mg capsules (A A H Pharmaceuticals Ltd) Topiramate 15mg Capsule Oral	04070402
Topiramate 200mg tablets (Actavis UK Ltd) Topiramate 200mg Tablet Oral	04070402
Topiramate 25mg tablets (Accord Healthcare Ltd) Topiramate 25mg Tablet Oral	04070402
Topiramate 100mg/5ml oral suspension Topiramate 20mg/1ml Oral suspension Oral	04070402
Topiramate 25mg tablets (Actavis UK Ltd) Topiramate 25mg Tablet Oral	04070402
Topiramate 15mg capsules (DE Pharmaceuticals) Topiramate 15mg Capsule Oral	04070402
Topiramate 25mg tablets (Teva UK Ltd) Topiramate 25mg Tablet Oral	04070402
Topamax 50mg tablets (Janssen-Cilag Ltd) Topiramate 50mg Tablet Oral	04070402
Topiramate 25mg capsules (DE Pharmaceuticals) Topiramate 25mg Capsule Oral	04070402
Topiramate 5mg/5ml oral solution Topiramate 1mg/1ml Oral solution Oral	04070402
Topiramate 25mg tablets Topiramate 25mg Tablet Oral	04070402
Topiramate 25mg/5ml oral suspension Topiramate 5mg/1ml Oral suspension Oral	04070402
Topiramate 20mg/5ml oral solution Topiramate 4mg/1ml Oral solution Oral	04070402
Topiramate 50mg/5ml oral suspension Topiramate 10mg/1ml Oral suspension Oral	04070402
Topiramate 12mg/5ml oral solution Topiramate 2.4mg/1ml Oral solution Oral	04070402
Topamax 50mg sprinkle capsules (Lexon (UK) Ltd) Topiramate 50mg Capsule Oral	04070402
Topiramate 25mg capsules (Teva UK Ltd) Topiramate 25mg Capsule Oral	04070402
Topiramate 12.5mg/5ml oral suspension Topiramate 2.5mg/1ml Oral suspension Oral	04070402
Topiramate 50mg capsules (Sandoz Ltd) Topiramate 50mg Capsule Oral	04070402
Topiramate 25mg capsules (Sigma Pharmaceuticals Plc) Topiramate 25mg Capsule Oral	04070402
Topiramate 15mg/5ml oral suspension Topiramate 3mg/1ml Oral suspension Oral	04070402
Topiramate 100mg tablets Topiramate 100mg Tablet Oral	04070402
Topiramate 100mg tablets (Milpharm Ltd)Topiramate100mgTabletOral	04070402
Topiramate 200mg tablets (Milpharm Ltd)Topiramate200mgTabletOral	04070402
Topiramate 50mg capsules (DE Pharmaceuticals)Topiramate50mgCapsuleOral	04070402

Topiramate 15mg capsules (Sandoz Ltd)Topiramate15mgCapsuleOral	04070402

DESCRIPTION	BNF1
Prochlorperazine 5mg tablets Prochlorperazine maleate 5mg Tablet Oral	04020101
Prochlorperazine 5mg/5ml oral solution Prochlorperazine mesilate 1mg/1ml Oral solution Oral	04020101
Proziere 5mg tablets (Ashbourne Pharmaceuticals Ltd) Prochlorperazine maleate 5mg Tablet Oral	04020101
Prochlorperazine 5mg tablets (Teva UK Ltd) Prochlorperazine maleate 5mg Tablet Oral	04020101
Stemetil 5mg tablets (Waymade Healthcare Plc) Prochlorperazine maleate 5mg Tablet Oral	04020101
Stemetil 5mg/5ml syrup (Sanofi) Prochlorperazine mesilate 1mg/1ml Oral solution Oral	04020101
Prochlorperazine 5mg tablets (Bristol Laboratories Ltd) Prochlorperazine maleate 5mg Tablet Oral	04020101
Prochlorperazine 5mg Tablet (Teva UK Ltd) Prochlorperazine maleate 5mg Tablet Oral	04020100
Stemetil 5mg tablets (Sanofi) Prochlorperazine maleate 5mg Tablet Oral	04020101
Stemetil 5mg tablets (Mawdsley-Brooks & Company Ltd) Prochlorperazine maleate 5mg Tablet Oral	04020101
Prochlorperazine 5mg tablets (Mylan) Prochlorperazine maleate 5mg Tablet Oral	04020101
Stemetil 5mg Tablet (Castlemead Healthcare Ltd) Prochlorperazine maleate 5mg Tablet Oral	04020100
Prochlorperazine 5mg tablets (Actavis UK Ltd) Prochlorperazine maleate 5mg Tablet Oral	04020101
Stemetil 5mg tablets (Sigma Pharmaceuticals Plc) Prochlorperazine maleate 5mg Tablet Oral	04020101
Prochlorperazine 5mg tablets (IVAX Pharmaceuticals UK Ltd) Prochlorperazine maleate 5mg Tablet Oral	04020101
Prochlorperazine 5mg tablets (Medreich Plc) Prochlorperazine maleate 5mg Tablet Oral	04020101
Prochlorperazine 5mg tablets (Waymade Healthcare Plc) Prochlorperazine maleate 5mg Tablet Oral	04020101
Stemetil 5mg/5ml Oral solution (Castlemead Healthcare Ltd) Prochlorperazine mesilate 1mg/1ml Oral solution Oral	04020100
Prochlorperazine 5mg tablets (Sigma Pharmaceuticals Plc) Prochlorperazine maleate 5mg Tablet Oral	04020101
Prochlorperazine 5mg tablets (Genesis Pharmaceuticals Ltd) Prochlorperazine maleate 5mg Tablet Oral	04020101
Prochlorperazine 5mg tablets (A A H Pharmaceuticals Ltd) Prochlorperazine maleate 5mg Tablet Oral	04020101
Stemetil 5mg tablets (DE Pharmaceuticals) Prochlorperazine maleate 5mg Tablet Oral	04020101
Prochlorperazine maleate 10mg modified release capsule Prochlorperazine Maleate 10mg Modified Release Capsules Oral	04020100
Prochlorperazine 5mg tablets (DE Pharmaceuticals) Prochlorperazine maleate 5mg Tablet Oral	04020101
Stemetil 5mg tablets (Lexon (UK) Ltd) Prochlorperazine maleate 5mg Tablet Oral	04020101

Prochlorperazine 5mg tablets (Dr Reddy's Laboratories (UK) Ltd) Prochlorperazine maleate 5mg Tablet Oral	04020101
Prochlorperazine 5mg tablets (Almus Pharmaceuticals Ltd) Prochlorperazine maleate 5mg Tablet Oral	04020101
Vertigon spansule 10 10mg Spansule (GlaxoSmithKline Consumer Healthcare) Prochlorperazine Maleate 10mg Spansule Oral	04020100
Prochlorperazine 5mg tablets (Phoenix Healthcare Distribution Ltd)Prochlorperazine maleate5mgTabletOral	04020101

DESCRIPTION	BNF1
Metoclopramide 10mg tablets Metoclopramide hydrochloride 10mg Tablet Oral	04065500
Maxolon 5mg/5ml Oral solution (Shire Pharmaceuticals Ltd) Metoclopramide hydrochloride 1mg/1ml Oral solution Oral	01020300
Metoclopramide 5mg/5ml oral solution sugar free (Sandoz Ltd) Metoclopramide hydrochloride 1mg/1ml Oral solution Oral	04065500
Metoclopramide 10mg tablets (Almus Pharmaceuticals Ltd) Metoclopramide hydrochloride 10mg Tablet Oral	04065500
Metoclopramide 10mg tablets (Alliance Healthcare (Distribution) Ltd) Metoclopramide hydrochloride 10mg Tablet Oral	04065500
Metoclopramide 5mg/5ml oral solution sugar free (Rosemont Pharmaceuticals Ltd) Metoclopramide hydrochloride 1mg/1ml Oral solution Oral	04065500
Metoclopramide 10mg tablets (A A H Pharmaceuticals Ltd) Metoclopramide hydrochloride 10mg Tablet Oral	04065500
Maxolon 5mg/5ml syrup (Amdipharm Plc) Metoclopramide hydrochloride 1mg/1ml Oral solution Oral	04065500
Metoclopramide 10mg Tablet (C P Pharmaceuticals Ltd) Metoclopramide hydrochloride 10mg Tablet Oral	01020300
Metoclopramide 10mg tablets (Actavis UK Ltd) Metoclopramide hydrochloride 10mg Tablet Oral	04065500
Metoclomex 10mg tablets (Actavis UK Ltd) Metoclopramide hydrochloride 10mg Tablet Oral	04065500
Metoclopramide 1mg/ml sugar free Oral solution Metoclopramide Hydrochloride 1mg/ml Oral Solution Oral	01020300
Maxolon 10mg Tablet (Shire Pharmaceuticals Ltd) Metoclopramide hydrochloride 10mg Tablet Oral	01020300
Metoclopramide 5mg/5ml oral solution sugar free (A A H Pharmaceuticals Ltd) Metoclopramide hydrochloride 1mg/1ml Oral solution Oral	04065500
Maxolon Paediatric 5mg/5ml liquid (Amdipharm Plc) Metoclopramide hydrochloride 1mg/1ml Oral solution Oral	04065500
Primperan 10mg Tablet (Berk Pharmaceuticals Ltd) Metoclopramide hydrochloride 10mg Tablet Oral	01020300
Metoclopramide 5mg/5ml oral solution sugar free (Alliance Healthcare (Distribution) Ltd) Metoclopramide hydrochloride 1mg/1ml Oral solution Oral	04065500
Metoclopramide 10mg tablets (Crescent Pharma Ltd) Metoclopramide hydrochloride 10mg Tablet Oral	04065500
Metoclopramide 10mg tablets (Accord Healthcare Ltd) Metoclopramide hydrochloride 10mg Tablet Oral	04065500
Metoclopramide 10mg Tablet (Celltech Pharma Europe Ltd) Metoclopramide hydrochloride 10mg Tablet Oral	01020300
Gastroflux 10mg tablets (Ashbourne Pharmaceuticals Ltd) Metoclopramide hydrochloride 10mg Tablet Oral	04065500
Metoclopramide 10mg tablets (Waymade Healthcare Plc) Metoclopramide hydrochloride 10mg Tablet Oral	04065500

Metoclopramide 10mg tablets (DE Pharmaceuticals) Metoclopramide hydrochloride 10mg Tablet Oral	04065500
Metox 10mg Tablet (M A Steinhard Ltd) Metoclopramide hydrochloride 10mg Tablet Oral	01020300
Maxolon 10mg tablets (Advanz Pharma) Metoclopramide hydrochloride 10mg Tablet Oral	04065500
Metoclopramide 5mg/5ml Oral solution Metoclopramide Hydrochloride 5mg/5ml Oral Solution Oral	01020300
Primperan 5mg/5ml Oral solution sugar free (Berk Pharmaceuticals Ltd) Metoclopramide hydrochloride 1mg/1ml Oral solution Oral	01020300
Metoclopramide 5mg/5ml oral solution sugar free Metoclopramide hydrochloride 1mg/1ml Oral solution Oral	04065500
Metoclopramide 10mg tablets (Kent Pharmaceuticals Ltd) Metoclopramide hydrochloride 10mg Tablet Oral	04065500
Metoclopramide 10mg tablets (Sigma Pharmaceuticals Plc) Metoclopramide hydrochloride 10mg Tablet Oral	04065500
Metoclopramide 10mg tablets (Teva UK Ltd) Metoclopramide hydrochloride 10mg Tablet Oral	04065500

DESCRIPTION	BNF1
Motilium 30mg suppositories (Zentiva) Domperidone 30mg Suppository Rectal	04065500
Domperidone 10mg tablets (Zentiva) Domperidone maleate 10mg Tablet Oral	04065500
Domperidone 1mg/ml oral suspension sugar free Domperidone 1mg/1ml Oral suspension Oral	04065500
Domperidone 1mg/ml oral suspension sugar free (A A H Pharmaceuticals Ltd) Domperidone 1mg/1ml Oral suspension Oral	04065500
Domperidone 1mg/ml oral suspension sugar free (Waymade Healthcare Plc) Domperidone 1mg/1ml Oral suspension Oral	04065500
Motilium Instants 10mg orodispersible tablets (McNeil Products Ltd) Domperidone 10mg Orodispersible tablet Oral	04065500
Motilium 1mg/ml oral suspension (Zentiva) Domperidone 1mg/1ml Oral suspension Oral	04065500
Domperidone 10mg tablets (PLIVA Pharma Ltd) Domperidone maleate 10mg Tablet Oral	04065500
Vivadone 10mg tablets (Lexon (UK) Ltd) Domperidone maleate 10mg Tablet Oral	04065500
Domperidone 10mg tablets (Sandoz Ltd) Domperidone maleate 10mg Tablet Oral	04065500
Domperidone 10mg tablets (A A H Pharmaceuticals Ltd) Domperidone maleate 10mg Tablet Oral	04065500
Domperidone 1mg/ml oral suspension sugar free (DE Pharmaceuticals) Domperidone 1mg/1ml Oral suspension Oral	04065500
Domperidone 800micrograms/5ml oral suspension Domperidone 160microgram/1ml Oral suspension Oral	04065500
Domperidone 10mg tablets (Strides Pharma UK Ltd) Domperidone maleate 10mg Tablet Oral	04065500
Domperidone 10mg tablets (Almus Pharmaceuticals Ltd) Domperidone maleate 10mg Tablet Oral	04065500
Evoxin 10mg Tablet (Sanofi-Synthelabo Ltd) Domperidone maleate 10mg Tablet Oral	01020300
Motilium 10mg tablets (Waymade Healthcare Plc) Domperidone maleate 10mg Tablet Oral	04065500
Domperidone 10mg tablets Domperidone maleate 10mg Tablet Oral	04065500
Domperidone 10mg tablets (Actavis UK Ltd) Domperidone maleate 10mg Tablet	04065500

Oral	
Domperidone 10mg tablets (Mylan) Domperidone maleate 10mg Tablet Oral	04065500
Domperidone 10mg tablets (Bristol Laboratories Ltd) Domperidone maleate 10mg Tablet Oral	04065500
Motilium 10 tablets (McNeil Products Ltd) Domperidone maleate 10mg Tablet Oral	04065500
Domperidone 10mg orodispersible tablets sugar free Domperidone 10mg Orodispersible tablet Oral	04065500
Domperidone 10mg/5ml oral suspension Domperidone 2mg/1ml Oral suspension Oral	04065500
Domperidone 10mg Tablet (Manx Pharma Ltd) Domperidone maleate 10mg Tablet Oral	01020300
Motilium 10mg tablets (Zentiva) Domperidone maleate 10mg Tablet Oral	04065500
Evoxin 30mg Suppository (Sanofi-Synthelabo Ltd) Domperidone 30mg Suppository Rectal	01020300
Domperidone 1.9mg/5ml oral suspension Domperidone 380microgram/1ml Oral suspension Oral	04065500
Domperidone 10mg tablets (Wockhardt UK Ltd) Domperidone maleate 10mg Tablet Oral	04065500
Domperidone 50mg/5ml oral suspension Domperidone 10mg/1ml Oral suspension Oral	04065500
Domperidone 10mg/2ml solution for injection ampoules Domperidone 5mg/1ml Solution for injection Intravenous	04065500
Domperidone 1mg/ml oral suspension sugar free (Alliance Healthcare (Distribution) Ltd) Domperidone 1mg/1ml Oral suspension Oral	04065500
Domperidone 10mg tablets (Sigma Pharmaceuticals Plc) Domperidone maleate 10mg Tablet Oral	04065500
Domperidone 1mg/ml oral suspension sugar free (Zentiva) Domperidone 1mg/1ml Oral suspension Oral	04065500
Domperidone 10mg tablets (Medreich Plc)Domperidone maleate10mgTabletOral	04065500

DESCRIPTION	BNF1
Cyclizine 50mg tablets Cyclizine hydrochloride 50mg Tablet Oral	04060000
Cyclizine 50mg tablets (DE Pharmaceuticals) Cyclizine hydrochloride 50mg Tablet Oral	04060000
Cyclizine 50mg tablets (A A H Pharmaceuticals Ltd) Cyclizine hydrochloride 50mg Tablet Oral	04060000
Cyclizine 50mg tablets (Morningside Healthcare Ltd) Cyclizine hydrochloride 50mg Tablet Oral	04060000
Valoid 50mg tablets (Amdipharm Plc) Cyclizine hydrochloride 50mg Tablet Oral	04060000
Cyclizine 50mg tablets (Actavis UK Ltd) Cyclizine hydrochloride 50mg Tablet Oral	04060000
Cyclizine 5mg/5ml oral suspension Cyclizine hydrochloride 1mg/1ml Oral suspension Oral	04060000
Cyclizine 50mg suppositories Cyclizine hydrochloride 50mg Suppository Rectal	04060000
Cyclizine 12.5mg/5ml oral solution Cyclizine hydrochloride 2.5mg/1ml Oral solution Oral	04060000
Cyclizine 50mg tablets (Alliance Healthcare (Distribution) Ltd) Cyclizine hydrochloride 50mg Tablet Oral	04060000
Cyclizine 50mg/1ml solution for injection ampoules (Hameln Pharmaceuticals	0406000

Ltd) Cyclizine lactate 50mg/1ml Solution for injection Intravenous/Intramuscular	0
Cyclizine 25mg/5ml oral suspension Cyclizine hydrochloride 5mg/1ml Oral suspension Oral	04060000
Cyclizine 50mg/1ml solution for injection ampoules (Martindale Pharmaceuticals Ltd) Cyclizine lactate 50mg/1ml Solution for injection Intravenous/Intramuscular	04060000
Cyclizine 50mg tablets (Phoenix Healthcare Distribution Ltd) Cyclizine hydrochloride 50mg Tablet Oral	04060000
Cyclizine 12.5mg/5ml oral suspension Cyclizine hydrochloride 2.5mg/1ml Oral suspension Oral	04060000
Cyclizine 50mg/1ml solution for injection ampoules (Alliance Healthcare (Distribution) Ltd) Cyclizine lactate 50mg/1ml Solution for injection Intravenous/Intramuscular	04060000
Cyclizine 50mg/1ml solution for injection ampoules Cyclizine lactate 50mg/1ml Solution for injection Intravenous/Intramuscular	04060000
Cyclizine 50mg/1ml solution for injection ampoules (A A H Pharmaceuticals Ltd) Cyclizine lactate 50mg/1ml Solution for injection Intravenous/Intramuscular	04060000
Cyclizine 50mg tablets (Sigma Pharmaceuticals Plc) Cyclizine hydrochloride 50mg Tablet Oral	04060000
Cyclizine 50mg/5ml oral suspension Cyclizine hydrochloride 10mg/1ml Oral suspension Oral	04060000
Cyclizine 50mg/5ml oral solution Cyclizine hydrochloride 10mg/1ml Oral solution Oral	04060000
Cyclizine 5mg/5ml oral solution Cyclizine hydrochloride 1mg/1ml Oral solution Oral	04060000
Valoid 50mg/1ml solution for injection ampoules (Advanz Pharma) Cyclizine lactate 50mg/1ml Solution for injection Intravenous/Intramuscular	04060000
Cyclizine 10mg/5ml oral solution Cyclizine hydrochloride 2mg/1ml Oral solution Oral	04060000
Cyclizine 50mg tablets (Advanz Pharma) Cyclizine hydrochloride 50mg Tablet Oral	04060000
Cyclizine 25mg suppositories Cyclizine hydrochloride 25mg Suppository Rectal	04060000
Cyclizine 50mg/1ml solution for injection ampoules (Advanz Pharma) Cyclizine lactate 50mg/1ml Solution for injection Intravenous/Intramuscular	04060000
Cyclizine 10mg/5ml oral suspension Cyclizine hydrochloride 2mg/1ml Oral suspension Oral	04060000
Cyclizine 50mg tablets (Teva UK Ltd) Cyclizine hydrochloride 50mg Tablet Oral	04060000

DESCRIPTION	BNF1
Tolfenamic acid 200mg Capsule Tolfenamic Acid 200mg Capsule Oral	04070401
Clotam Rapid 200mg tablets (Galen Ltd) Tolfenamic acid 200mg Tablet Oral	04070401
Tolfenamic acid 200mg tablets (A A H Pharmaceuticals Ltd) Tolfenamic acid 200mg Tablet Oral	04070401
Tolfenamic acid 200mg tablets Tolfenamic acid 200mg Tablet Oral	04070401
Clotam 200mg Capsule (Thames Laboratories Ltd) Tolfenamic Acid 200mg Capsule Oral	04070401

DESCRIPTION	BNF1

Epilim Chrono 500 tablets (Sanofi) Sodium valproate 500mg Modified-release tablet Oral	04080100
Sodium valproate 200mg gastro-resistant tablets Sodium valproate 200mg Gastro-resistant tablet Oral	04080100
Epilim Chrono 300 tablets (Sanofi) Sodium valproate 300mg Modified-release tablet Oral	04080100
Epilim ec 200mg Gastro-resistant tablet (Sanofi) Sodium valproate 200mg Gastro-resistant tablet Oral	04080100
Epilim Chrono 200 tablets (Sanofi) Sodium valproate 200mg Modified-release tablet Oral	04080100
Sodium valproate 500mg gastro-resistant tablets Sodium valproate 500mg Gastro-resistant tablet Oral	04080100
Epilim 500 gastro-resistant tablets (Sanofi) Sodium valproate 500mg Gastro-resistant tablet Oral	04080100
Depakote 250mg gastro-resistant tablets (Sanofi) Valproate semisodium 250mg Gastro-resistant tablet Oral	04020300
Valproic acid 500mg gastro-resistant tablets Valproate semisodium 500mg Gastro-resistant tablet Oral	04020300
Valproic acid 250mg gastro-resistant tablets Valproate semisodium 250mg Gastro-resistant tablet Oral	04020300
Sodium valproate 200mg/5ml oral solution Sodium valproate 40mg/1ml Oral solution Oral	04080100
Epilim 200mg/5ml syrup (Sanofi) Sodium valproate 40mg/1ml Oral solution Oral	04080100
Sodium valproate with valproic acid 200mg modified release tablets Sodium Valproate 200mg Modified Release Tablet Oral	04080100
Episenta 300mg modified-release capsules (Desitin Pharma Ltd) Sodium valproate 300mg Modified-release capsule Oral	04080100
Epilim Chronosphere MR 750mg granules sachets (Sanofi) Sodium valproate 750mg Modified-release granules Oral	04080100
Valproate sodium 500mg Gastro-resistant tablet (IVAX Pharmaceuticals UK Ltd) Sodium valproate 500mg Gastro-resistant tablet Oral	04080100
Valproic acid 500mg/5ml oral suspension Valproate semisodium 100mg/1ml Oral suspension Oral	04020300
Epilim Chronosphere MR 500mg granules sachets (Sanofi) Sodium valproate 500mg Modified-release granules Oral	04080100
Sodium valproate 200mg gastro-resistant tablets (Teva UK Ltd) Sodium valproate 200mg Gastro-resistant tablet Oral	04080100
Epilim Chrono 300 tablets (Lexon (UK) Ltd) Sodium valproate 300mg Modified-release tablet Oral	04080100
Sodium valproate 500mg modified-release tablets Sodium valproate 500mg Modified-release tablet Oral	04080100
Sodium valproate 500mg gastro-resistant tablets (Zentiva) Sodium valproate 500mg Gastro-resistant tablet Oral	04080100
Epilim ec 500mg Gastro-resistant tablet (Sanofi) Sodium valproate 500mg Gastro-resistant tablet Oral	04080100
Sodium valproate 200mg/5ml oral solution sugar free (Wockhardt UK Ltd) Sodium valproate 40mg/1ml Oral solution Oral	04080100
Convulex 500mg gastro-resistant capsules (Pfizer Ltd) Valproic acid 500mg Gastro-resistant capsule Oral	04070402
Sodium valproate 500mg modified-release tablets (J M McGill Ltd) Sodium valproate 500mg Modified-release tablet Oral	04080100
Sodium valproate with valproic acid 500mg modified release tablets Sodium Valproate 500mg Modified Release Tablet Oral	04080100

Sodium valproate 200mg gastro-resistant tablets (Alliance Healthcare (Distribution) Ltd) Sodium valproate 200mg Gastro-resistant tablet Oral	04080100
Valproic acid 500mg/5ml oral solution Valproate semisodium 100mg/1ml Oral solution Oral	04020300
Epilim Chrono 300 tablets (DE Pharmaceuticals) Sodium valproate 300mg Modified-release tablet Oral	04080100
Epilim Chrono 300 tablets (Waymade Healthcare Plc) Sodium valproate 300mg Modified-release tablet Oral	04080100
Epilim Chrono 200 tablets (Lexon (UK) Ltd) Sodium valproate 200mg Modified-release tablet Oral	04080100
Epilim 200 gastro-resistant tablets (Sanofi) Sodium valproate 200mg Gastro-resistant tablet Oral	04080100
Convulex 300mg gastro-resistant capsules (Pfizer Ltd) Valproic acid 300mg Gastro-resistant capsule Oral	04070402
Orlept SF 200mg/5ml liquid (Wockhardt UK Ltd) Sodium valproate 40mg/1ml Oral solution Oral	04080100
Sodium valproate CR 300mg Tablet (Hillcross Pharmaceuticals Ltd) Sodium Valproate 300mg Tablet	04080100
Epilim Chrono 500 tablets (Lexon (UK) Ltd) Sodium valproate 500mg Modified-release tablet Oral	04080100
Sodium valproate 750mg modified-release granules sachets sugar free Sodium valproate 750mg Modified-release granules Oral	04080100
Epilim Chronosphere MR 250mg granules sachets (Sanofi) Sodium valproate 250mg Modified-release granules Oral	04080100
Sodium valproate 500mg gastro-resistant tablets (Wockhardt UK Ltd) Sodium valproate 500mg Gastro-resistant tablet Oral	04080100
Sodium valproate 200mg/5ml Oral solution (Sterwin Medicines) Sodium valproate 40mg/1ml Oral solution Oral	04080100
Sodium valproate 300mg suppositories Sodium valproate 300mg Suppository Rectal	04080100
Epilim Chrono 500 tablets (Waymade Healthcare Plc) Sodium valproate 500mg Modified-release tablet Oral	04080100
Belvo 250mg gastro-resistant tablets (Consilient Health Ltd) Valproate semisodium 250mg Gastro-resistant tablet Oral	04020300
Epilim Chrono 200 tablets (Waymade Healthcare Plc) Sodium valproate 200mg Modified-release tablet Oral	04080100
Sodium valproate 500mg modified-release granules sachets sugar free Sodium valproate 500mg Modified-release granules Oral	04080100
Epilim Chrono 500 tablets (DE Pharmaceuticals) Sodium valproate 500mg Modified-release tablet Oral	04080100
Sodium valproate 500mg gastro-resistant tablets (Arrow Generics Ltd) Sodium valproate 500mg Gastro-resistant tablet Oral	04080100
Depakote 500mg gastro-resistant tablets (Sanofi) Valproate semisodium 500mg Gastro-resistant tablet Oral	04020300
Orlept 200mg gastro-resistant tablets (Wockhardt UK Ltd) Sodium valproate 200mg Gastro-resistant tablet Oral	04080100
Syonell 250mg gastro-resistant tablets (Lupin Healthcare (UK) Ltd) Valproate semisodium 250mg Gastro-resistant tablet Oral	04020300
Sodium valproate 200mg/5ml oral solution sugar free (A A H Pharmaceuticals Ltd) Sodium valproate 40mg/1ml Oral solution Oral	04080100
Episenta 1000mg modified-release granules sachets (Desitin Pharma Ltd) Sodium valproate 1gram Modified-release granules Oral	04020300
Sodium valproate oral solution Sodium Valproate Oral Liquid Oral	04080100

Valproate sodium 200mg Gastro-resistant tablet (IVAX Pharmaceuticals UK Ltd) Sodium valproate 200mg Gastro-resistant tablet Oral	04080100
Epilim 200mg/5ml syrup (Lexon (UK) Ltd) Sodium valproate 40mg/1ml Oral solution Oral	04080100
Orlept 500mg gastro-resistant tablets (Wockhardt UK Ltd) Sodium valproate 500mg Gastro-resistant tablet Oral	04080100
Epival CR 500mg tablets (Healthcare Pharma Ltd) Sodium valproate 500mg Modified-release tablet Oral	04080100
Sodium valproate 150mg modified-release capsules Sodium valproate 150mg Modified-release capsule Oral	04080100
Sodium valproate 500mg Tablet (Sterwin Medicines) Sodium valproate 500mg Gastro-resistant tablet Oral	04080100
Sodium valproate with valproic acid 300mg modified release tablets Sodium Valproate 300mg Modified Release Tablet Oral	04080100
Sodium valproate CR 500mg Tablet (Hillcross Pharmaceuticals Ltd) Sodium Valproate 500mg Tablet Oral	04080100
Sodium valproate 200mg gastro-resistant tablets (Zentiva) Sodium valproate 200mg Gastro-resistant tablet Oral	04080100
Epilim Chrono 200 tablets (DE Pharmaceuticals) Sodium valproate 200mg Modified-release tablet Oral	04080100
Sodium valproate with valproic acid 750mg modified release granules Sodium Valproate 750mg Modified Release Granules Oral	04080100
Valproic acid 250mg/5ml oral suspension Valproate semisodium 50mg/1ml Oral suspension Oral	04020300
Sodium valproate 500mg/5ml oral suspension Sodium valproate 100mg/1ml Oral suspension Oral	04080100
Sodium valproate 200mg gastro-resistant tablets (Wockhardt UK Ltd) Sodium valproate 200mg Gastro-resistant tablet Oral	04080100
Sodium valproate 200mg/5ml Oral solution (IVAX Pharmaceuticals UK Ltd) Sodium valproate 40mg/1ml Oral solution Oral	04080100
Sodium valproate 250mg modified-release granules sachets sugar free Sodium valproate 250mg Modified-release granules Oral	04080100
Sodium valproate 1g modified-release granules sachets sugar free Sodium valproate 1gram Modified-release granules Oral	04080100
Sodium valproate with valproic acid 500mg modified release granules Sodium Valproate 500mg Modified Release Granules Oral	04080100
Sodium valproate 500mg gastro-resistant tablets (Teva UK Ltd) Sodium valproate 500mg Gastro-resistant tablet Oral	04080100
Sodium valproate 300mg modified-release capsules Sodium valproate 300mg Modified-release capsule Oral	04080100
Sodium valproate 200mg Tablet (Sterwin Medicines) Sodium valproate 200mg Gastro-resistant tablet Oral	04080100
Sodium valproate 200mg/5ml oral solution sugar free (Zentiva) Sodium valproate 40mg/1ml Oral solution Oral	04080100
Syonell 500mg gastro-resistant tablets (Lupin Healthcare (UK) Ltd) Valproate semisodium 500mg Gastro-resistant tablet Oral	04020300
Sodium valproate 600mg/5ml oral solution Sodium valproate 120mg/1ml Oral solution Oral	04080100
Valproic acid 250mg/5ml oral solution Valproate semisodium 50mg/1ml Oral solution Oral	04020300
Epilim 500 gastro-resistant tablets (Waymade Healthcare Plc) Sodium valproate 500mg Gastro-resistant tablet Oral	04080100
Sodium valproate 200mg gastro-resistant tablets (A A H Pharmaceuticals Ltd) Sodium valproate 200mg Gastro-resistant tablet Oral	04080100

Sodium valproate 500mg modified-release tablets (Alliance Healthcare (Distribution) Ltd) Sodium valproate 500mg Modified-release tablet Oral	04080100
Epilim Chrono 200 tablets (Sigma Pharmaceuticals Plc) Sodium valproate 200mg Modified-release tablet Oral	04080100
Sodium valproate 300mg modified-release tablets (Sigma Pharmaceuticals Plc) Sodium valproate 300mg Modified-release tablet Oral	04080100
Epilim Chronosphere MR 1000mg granules sachets (Sanofi) Sodium valproate 1gram Modified-release granules Oral	04080100
Sodium valproate 200mg modified-release tablets Sodium valproate 200mg Modified-release tablet Oral	04080100
Episenta 500mg modified-release granules sachets (Desitin Pharma Ltd) Sodium valproate 500mg Modified-release granules Oral	04020300
Epilim 200mg/5ml syrup (DE Pharmaceuticals) Sodium valproate 40mg/1ml Oral solution Oral	04080100
Sodium valproate with valproic acid 250mg modified release granules Sodium Valproate 250mg Modified Release Granules Oral	04080100
Sodium valproate 500mg gastro-resistant tablets (A A H Pharmaceuticals Ltd) Sodium valproate 500mg Gastro-resistant tablet Oral	04080100
Sodium valproate 500mg Gastro-resistant tablet (C P Pharmaceuticals Ltd) Sodium valproate 500mg Gastro-resistant tablet Oral	04080100
Sodium valproate 500mg gastro-resistant tablets (Mylan) Sodium valproate 500mg Gastro-resistant tablet Oral	04080100
Sodium valproate 200mg gastro-resistant tablets (Mylan) Sodium valproate 200mg Gastro-resistant tablet Oral	04080100
Sodium valproate 200mg gastro-resistant tablets (Waymade Healthcare Plc) Sodium valproate 200mg Gastro-resistant tablet Oral	04080100
Sodium valproate 500mg modified-release tablets (DE Pharmaceuticals) Sodium valproate 500mg Modified-release tablet Oral	04080100
Epilim 200mg/5ml liquid (Sanofi) Sodium valproate 40mg/1ml Oral solution Oral	04080100
Sodium valproate with valproic acid 1000mg modified release granules Sodium Valproate 1000mg Modified Release Granules Oral	04080100
Sodium valproate 200mg gastro-resistant tablets (IVAX Pharmaceuticals UK Ltd) Sodium valproate 200mg Gastro-resistant tablet Oral	04080100
Epival CR 300mg tablets (Healthcare Pharma Ltd) Sodium valproate 300mg Modified-release tablet Oral	04080100
Sodium valproate 200mg/5ml oral solution sugar free Sodium valproate 40mg/1ml Oral solution Oral	04080100
Sodium valproate 200mg/5ml Oral solution (Hillcross Pharmaceuticals Ltd) Sodium valproate 40mg/1ml Oral solution Oral	04080100
Belvo 500mg gastro-resistant tablets (Consilient Health Ltd) Valproate semisodium 500mg Gastro-resistant tablet Oral	04020300
Sodium valproate 300mg modified-release tablets Sodium valproate 300mg Modified-release tablet Oral	04080100
Sodium valproate 200mg gastro-resistant tablets (Actavis UK Ltd) Sodium valproate 200mg Gastro-resistant tablet Oral	04080100
Sodium valproate 500mg gastro-resistant tablets (Alliance Healthcare (Distribution) Ltd)Sodium valproate500mgGastro-resistant tabletOral	04080100
Sodium valproate 500mg gastro-resistant tablets (IVAX Pharmaceuticals UK Ltd)Sodium valproate500mgGastro-resistant tabletOral	04080100
Sodium valproate 300mg modified-release tablets (Colorama Pharmaceuticals Ltd)Sodium valproate300mgModified-release tabletOral	04080100
Sodium valproate 200mg modified-release tablets (DE Pharmaceuticals)Sodium valproate200mgModified-release tabletOral	04080100

Sodium valproate 300mg modified-release tablets (Ennogen Healthcare Ltd)Sodium valproate300mgModified-release tabletOral	04080100
Sodium valproate 200mg modified-release tablets (Ennogen Healthcare Ltd)Sodium valproate200mgModified-release tabletOral	04080100
Epilim 200mg/5ml liquid (Waymade Healthcare Plc)Sodium valproate40mg/1mlOral solutionOral	04080100

DESCRIPTION	BNF1
Amitriptyline 10mg tablets Amitriptyline hydrochloride 10mg Tablet Oral	04030100
Amitriptyline 25mg tablets Amitriptyline hydrochloride 25mg Tablet Oral	04030100
Amitriptyline 50mg tablets Amitriptyline hydrochloride 50mg Tablet Oral	04030100
Amitriptyline 25mg modified-release capsules Amitriptyline hydrochloride 25mg Modified-release capsule Oral	00000000
Lentizol 50mg modified-release capsules (Pfizer Ltd) Amitriptyline hydrochloride 50mg Modified-release capsule Oral	00000000
Amitriptyline 10mg tablets (Teva UK Ltd) Amitriptyline hydrochloride 10mg Tablet Oral	04030100
Perphenazine 2mg with Amitriptyline 25mg tablet Amitriptyline Hydrochloride/Perphenazine 2mg + 25mg Tablets Oral	04020100
Amitriptyline 25mg / Perphenazine 2mg tablets Amitriptyline hydrochloride/Perphenazine 25mg + 2mg Tablet Oral	04030100
Tryptizol mr 75mg Modified-release capsule (Merck Sharp & Dohme Ltd) Amitriptyline Hydrochloride 75mg Modified-Release Capsule Oral	04030100
Amitriptyline 10mg tablets (Almus Pharmaceuticals Ltd) Amitriptyline hydrochloride 10mg Tablet Oral	04030100
Amitriptyline 10mg/5ml Oral solution (Rosemont Pharmaceuticals Ltd) Amitriptyline Hydrochloride 10mg/5ml Oral Solution Oral	04030100
Amitriptyline 50mg/5ml oral solution sugar free (Rosemont Pharmaceuticals Ltd) Amitriptyline hydrochloride 10mg/1ml Oral solution Oral	04030100
AMITRIPTYLINE	00000000
Amitriptyline 25mg tablets (Alliance Healthcare (Distribution) Ltd) Amitriptyline hydrochloride 25mg Tablet Oral	04030100
Amitriptyline 75mg modified-release capsules Amitriptyline Hydrochloride 75mg Modified Release Capsules Oral	04030100
Amitriptyline 50mg Tablet (Berk Pharmaceuticals Ltd) Amitriptyline hydrochloride 50mg Tablet Oral	04030100
Amitriptyline 10mg/5ml oral solution sugar free (A A H Pharmaceuticals Ltd) Amitriptyline hydrochloride 2mg/1ml Oral solution Oral	04030100
Amitriptyline 10mg tablets (DE Pharmaceuticals) Amitriptyline hydrochloride 10mg Tablet Oral	04030100
Amitriptyline 25mg tablets (Crescent Pharma Ltd) Amitriptyline hydrochloride 25mg Tablet Oral	04030100
Amitriptyline 25mg Tablet (Sussex Pharmaceutical Ltd) Amitriptyline hydrochloride 25mg Tablet Oral	04030100
AMITRIPTYLINE 100 MG TAB	00000000
Amitriptyline 25mg Tablet (Berk Pharmaceuticals Ltd) Amitriptyline hydrochloride	0403010

25mg Tablet Oral	0
Amitriptyline 25mg tablets (Almus Pharmaceuticals Ltd) Amitriptyline hydrochloride 25mg Tablet Oral	0403010 0
Lentizol 25mg modified-release capsules (Pfizer Ltd) Amitriptyline hydrochloride 25mg Modified-release capsule Oral	0000000 0
Amitriptyline 25mg tablets (Phoenix Healthcare Distribution Ltd) Amitriptyline hydrochloride 25mg Tablet Oral	0403010 0
Amitriptyline 10mg tablets (Arrow Generics Ltd) Amitriptyline hydrochloride 10mg Tablet Oral	0403010 0
Amitriptyline 50mg tablets (Teva UK Ltd) Amitriptyline hydrochloride 50mg Tablet Oral	0403010 0
Amitriptyline 50mg tablets (Almus Pharmaceuticals Ltd) Amitriptyline hydrochloride 50mg Tablet Oral	0403010 0
Amitriptyline 5mg/5ml oral suspension Amitriptyline hydrochloride 1mg/1ml Oral suspension Oral	0403010 0
Amitriptyline 10mg Tablet (Sussex Pharmaceutical Ltd) Amitriptyline hydrochloride 10mg Tablet Oral	0403010 0
Amitriptyline 100mg/5ml oral solution Amitriptyline hydrochloride 20mg/1ml Oral solution Oral	0403010 0
Amitriptyline 50mg/5ml oral solution sugar free Amitriptyline hydrochloride 10mg/1ml Oral solution Oral	0403010 0
Amitriptyline 10mg tablets (Genesis Pharmaceuticals Ltd) Amitriptyline hydrochloride 10mg Tablet Oral	0403010 0
Tryptizol 10mg/5ml sugar free Oral solution (Merck Sharp and Dohme Ltd) Amitriptyline Hydrochloride 10mg/5ml Oral Solution Oral	0403010 0
AMITRIPTYLINE S/R	0000000 0
Triptafen m 2mg+10mg Tablet (Goldshield Pharmaceuticals Ltd) Amitriptyline hydrochloride/Perphenazine 10mg + 2mg Tablet Oral	0402010 0
Amitriptyline 10mg tablets (Actavis UK Ltd) Amitriptyline hydrochloride 10mg Tablet Oral	0403010 0
Amitriptyline 25mg tablets (Kent Pharmaceuticals Ltd) Amitriptyline hydrochloride 25mg Tablet Oral	0403010 0
Amitriptyline 25mg tablets (Teva UK Ltd) Amitriptyline hydrochloride 25mg Tablet Oral	0403010 0
Amitriptyline 75mg/5ml oral solution Amitriptyline hydrochloride 15mg/1ml Oral solution Oral	0403010 0
Amitriptyline 10mg/5ml oral solution sugar free Amitriptyline hydrochloride 2mg/1ml Oral solution Oral	0403010 0
Amitriptyline 25mg tablets (IVAX Pharmaceuticals UK Ltd) Amitriptyline hydrochloride 25mg Tablet Oral	0403010 0
Amitriptyline 10mg tablets (Wockhardt UK Ltd) Amitriptyline hydrochloride 10mg Tablet Oral	0403010 0
Amitriptyline 10mg/5ml oral solution sugar free (Rosemont Pharmaceuticals Ltd) Amitriptyline hydrochloride 2mg/1ml Oral solution Oral	0000000 0
Domical 25mg Tablet (Berk Pharmaceuticals Ltd) Amitriptyline hydrochloride 25mg Tablet Oral	0403010 0
Domical 50mg Tablet (Berk Pharmaceuticals Ltd) Amitriptyline hydrochloride 50mg Tablet Oral	0403010 0
Amitriptyline 10mg tablets (Sigma Pharmaceuticals Plc) Amitriptyline hydrochloride 10mg Tablet Oral	0403010 0
AMITRIPTYLINE S/F	0000000 0

Tryptizol 10mg Tablet (Merck Sharp & Dohme Ltd) Amitriptyline hydrochloride 10mg Tablet Oral	04030100
Amitriptyline 25mg tablets (Ranbaxy (UK) Ltd) Amitriptyline hydrochloride 25mg Tablet Oral	04030100
AMITRIPTYLINE S/F 25 MG/5ML SYR	00000000
Amitriptyline oral solution Amitriptyline Hydrochloride Oral Liquid Oral	04030100
Amitriptyline 25mg/5ml oral solution sugar free Amitriptyline hydrochloride 5mg/1ml Oral solution Oral	04030100
Amitriptyline 25mg tablets (Arrow Generics Ltd) Amitriptyline hydrochloride 25mg Tablet Oral	04030100
Amitriptyline 50mg tablets (DE Pharmaceuticals) Amitriptyline hydrochloride 50mg Tablet Oral	04030100
Amitriptyline 10mg tablets (Waymade Healthcare Plc) Amitriptyline hydrochloride 10mg Tablet Oral	04030100
Amitriptyline 12.5mg / Chlordiazepoxide 5mg capsules Amitriptyline Hydrochloride/Chlordiazepoxide 12.5mg + 5mg Capsules Oral	04030100
Domical 10mg Tablet (Berk Pharmaceuticals Ltd) Amitriptyline hydrochloride 10mg Tablet Oral	04030100
Amitriptyline 10mg/5ml oral suspension Amitriptyline hydrochloride 2mg/1ml Oral suspension Oral	04030100
Limbitrol 10 Capsule (Roche Products Ltd) Amitriptyline Hydrochloride/Chlordiazepoxide Capsule Oral	04030100
Amitriptyline 10mg tablets (Alliance Healthcare (Distribution) Ltd) Amitriptyline hydrochloride 10mg Tablet Oral	04030100
Amitriptyline 25mg Tablet (Regent Laboratories Ltd) Amitriptyline hydrochloride 25mg Tablet Oral	04030100
Amitriptyline 25mg tablets (Actavis UK Ltd) Amitriptyline hydrochloride 25mg Tablet Oral	04030100
Amitriptyline 25mg Tablet (Crosspharma Ltd) Amitriptyline hydrochloride 25mg Tablet Oral	04030100
Amitriptyline 25mg/5ml oral solution sugar free (DE Pharmaceuticals) Amitriptyline hydrochloride 5mg/1ml Oral solution Oral	04030100
TRYPTIZOL	00000000
Amitriptyline 10mg tablets (Phoenix Healthcare Distribution Ltd) Amitriptyline hydrochloride 10mg Tablet Oral	04030100
Amitriptyline 10mg tablets (Accord Healthcare Ltd) Amitriptyline hydrochloride 10mg Tablet Oral	04030100
Amitriptyline 50mg/5ml oral solution sugar free (Wockhardt UK Ltd) Amitriptyline hydrochloride 10mg/1ml Oral solution Oral	04030100
Amitriptyline 50mg tablets (Accord Healthcare Ltd) Amitriptyline hydrochloride 50mg Tablet Oral	04030100
Amitriptyline 10mg tablets (NorthStar Healthcare Unlimited Company) Amitriptyline hydrochloride 10mg Tablet Oral	04030100
AMITRIPTYLINE 75 MG TAB	00000000
Limbitrol 5 Capsule (Roche Products Ltd) Amitriptyline Hydrochloride/Chlordiazepoxide Capsule Oral	04030100
Amitriptyline 2.5mg/5ml oral solution Amitriptyline hydrochloride 500microgram/1ml Oral solution Oral	04030100
Amitriptyline 10mg/5ml sugar free oral solution Amitriptyline Hydrochloride 10mg/5ml Oral Solution Sugar-Free Oral	04030100

Amitriptyline 25mg tablets (DE Pharmaceuticals) Amitriptyline hydrochloride 25mg Tablet Oral	04030100
Tryptizol 50mg Tablet (Merck Sharp & Dohme Ltd) Amitriptyline hydrochloride 50mg Tablet Oral	04030100
Amitriptyline 10mg/5ml oral solution Amitriptyline hydrochloride 2mg/1ml Oral solution Oral	04030100
Amitriptyline 50mg tablets (IVAX Pharmaceuticals UK Ltd) Amitriptyline hydrochloride 50mg Tablet Oral	04030100
Elavil 10mg Tablet (DDSA Pharmaceuticals Ltd) Amitriptyline hydrochloride 10mg Tablet Oral	04030100
Amitriptyline 50mg modified-release capsules Amitriptyline hydrochloride 50mg Modified-release capsule Oral	00000000
Amitriptyline 50mg tablets (A A H Pharmaceuticals Ltd) Amitriptyline hydrochloride 50mg Tablet Oral	04030100
Amitriptyline 50mg tablets (Kent Pharmaceuticals Ltd) Amitriptyline hydrochloride 50mg Tablet Oral	04030100
Amitriptyline 25mg tablets (Sandoz Ltd) Amitriptyline hydrochloride 25mg Tablet Oral	04030100
Amitriptyline 10mg tablets (IVAX Pharmaceuticals UK Ltd) Amitriptyline hydrochloride 10mg Tablet Oral	04030100
Amitriptyline 10mg tablets (Kent Pharmaceuticals Ltd) Amitriptyline hydrochloride 10mg Tablet Oral	04030100
Amitriptyline 5mg/5ml oral solution Amitriptyline hydrochloride 1mg/1ml Oral solution Oral	04030100
Amitriptyline 10mg / Perphenazine 2mg tablets Amitriptyline hydrochloride/Perphenazine 10mg + 2mg Tablet Oral	00000000
Amitriptyline 25mg/5ml oral solution sugar free (Rosemont Pharmaceuticals Ltd) Amitriptyline hydrochloride 5mg/1ml Oral solution Oral	04030100
Amitriptyline 25mg tablets (Mawdsley-Brooks & Company Ltd) Amitriptyline hydrochloride 25mg Tablet Oral	04030100
Amitriptyline 25mg/5ml oral solution sugar free (Wockhardt UK Ltd) Amitriptyline hydrochloride 5mg/1ml Oral solution Oral	04030100
Amitriptyline 25mg/5ml oral solution sugar free (Alliance Healthcare (Distribution) Ltd) Amitriptyline hydrochloride 5mg/1ml Oral solution Oral	04030100
Amitriptyline 10mg tablets (A A H Pharmaceuticals Ltd) Amitriptyline hydrochloride 10mg Tablet Oral	04030100
Amitriptyline 25mg tablets (Wockhardt UK Ltd) Amitriptyline hydrochloride 25mg Tablet Oral	04030100
Triptafen-M tablets (Mercury Pharma Group Ltd) Amitriptyline hydrochloride/Perphenazine 10mg + 2mg Tablet Oral	00000000
Amitriptyline 25mg / Chlordiazepoxide 10mg capsules Amitriptyline Hydrochloride/Chlordiazepoxide 25mg + 10mg Capsules Oral	04030100
Amitriptyline 10mg/5ml oral solution sugar free (Alliance Healthcare (Distribution) Ltd) Amitriptyline hydrochloride 2mg/1ml Oral solution Oral	04030100
Amitriptyline 10mg Tablet (Berk Pharmaceuticals Ltd) Amitriptyline hydrochloride 10mg Tablet Oral	04030100
AMITRIPTYLINE 300 MG TAB	00000000
Amitriptyline 25mg Tablet (Celltech Pharma Europe Ltd) Amitriptyline hydrochloride 25mg Tablet Oral	04030100
Amitriptyline 25mg tablets (Accord Healthcare Ltd) Amitriptyline hydrochloride 25mg Tablet Oral	04030100
Amitriptyline 50mg tablets (Sigma Pharmaceuticals Plc) Amitriptyline hydrochloride 50mg Tablet Oral	04030100

Amitriptyline 25mg tablets (Sigma Pharmaceuticals Plc) Amitriptyline hydrochloride 25mg Tablet Oral	04030100
Tryptizol 25mg Tablet (Merck Sharp & Dohme Ltd) Amitriptyline hydrochloride 25mg Tablet Oral	04030100
Amitriptyline 50mg tablets (Wockhardt UK Ltd) Amitriptyline hydrochloride 50mg Tablet Oral	04030100
Amitriptyline 25mg tablets (A A H Pharmaceuticals Ltd) Amitriptyline hydrochloride 25mg Tablet Oral	04030100
Amitriptyline 25mg tablets (Genesis Pharmaceuticals Ltd) Amitriptyline hydrochloride 25mg Tablet Oral	04030100
Perphenazine 2mg with Amitriptyline 10mg tablet Amitriptyline Hydrochloride/Perphenazine 2mg + 10mg Tablets Oral	04020100
Amitriptyline 10mg tablets (Mawdsley-Brooks & Company Ltd) Amitriptyline hydrochloride 10mg Tablet Oral	04030100
Amitriptyline 10mg/5ml oral solution sugar free (Wockhardt UK Ltd) Amitriptyline hydrochloride 2mg/1ml Oral solution Oral	04030100
Triptafen tablets (Advanz Pharma) Amitriptyline hydrochloride/Perphenazine 25mg + 2mg Tablet Oral	04030100
Amitriptyline 50mg tablets (Arrow Generics Ltd)Amitriptyline hydrochloride50mgTabletOral	04030100

DESCRIPTION	BNF1
Diclofenac sodium 50mg gastro-resistant tablets Diclofenac sodium 50mg Gastro-resistant tablet Oral	10010100
Voltarol 50mg Tablet (Novartis Pharmaceuticals UK Ltd) Diclofenac sodium 50mg Gastro-resistant tablet Oral	10010100
Diclofenac sodium 50mg gastro-resistant tablets Diclofenac Sodium 50mg EC Tablets Oral	10010100
Voltarol 50mg gastro-resistant tablets (Novartis Pharmaceuticals UK Ltd) Diclofenac sodium 50mg Gastro-resistant tablet Oral	10010100
Voltarol 25mg Tablet (Novartis Pharmaceuticals UK Ltd) Diclofenac sodium 25mg Gastro-resistant tablet Oral	10010100
Voltarol 25mg gastro-resistant tablets (Novartis Pharmaceuticals UK Ltd) Diclofenac sodium 25mg Gastro-resistant tablet Oral	10010100
Lofensaid 50mg gastro-resistant tablets (Opus Pharmaceuticals Ltd) Diclofenac sodium 50mg Gastro-resistant tablet Oral	10010100
Diclofenac 50mg Gastro-resistant tablet (Genus Pharmaceuticals Ltd) Diclofenac sodium 50mg Gastro-resistant tablet Oral	10010100
Diclofenac potassium 50mg tablets (Alliance Healthcare (Distribution) Ltd) Diclofenac potassium 50mg Tablet Oral	10010100
Diclofenac sodium 25mg gastro-resistant tablets (Phoenix Healthcare Distribution Ltd) Diclofenac sodium 25mg Gastro-resistant tablet Oral	10010100
Diclofenac potassium 50mg tablets (Phoenix Healthcare Distribution Ltd) Diclofenac potassium 50mg Tablet Oral	10010100
Dicloflex 25mg gastro-resistant tablets (Dexcel-Pharma Ltd) Diclofenac sodium 25mg Gastro-resistant tablet Oral	10010100
Diclofenac potassium 50mg tablets (A A H Pharmaceuticals Ltd) Diclofenac potassium 50mg Tablet Oral	10010100
Voltarol 50mg dispersible tablets (DE Pharmaceuticals) Diclofenac sodium 50mg Dispersible tablet Oral	10010100
Diclofenac 50mg dispersible tablets sugar free (DE Pharmaceuticals) Diclofenac sodium 50mg Dispersible tablet Oral	10010100
Voltarol Rapid 50mg tablets (Lexon (UK) Ltd) Diclofenac potassium 50mg Tablet	1001010

Oral	0
Diclofenac sodium 50mg gastro-resistant tablets (Kent Pharmaceuticals Ltd) Diclofenac sodium 50mg Gastro-resistant tablet Oral	10010100
Voltarol Rapid 50mg tablets (Novartis Pharmaceuticals UK Ltd) Diclofenac potassium 50mg Tablet Oral	10010100
Diclofenac sodium 25mg gastro-resistant tablets (Waymade Healthcare Plc) Diclofenac sodium 25mg Gastro-resistant tablet Oral	10010100
Diclofenac 25mg Gastro-resistant tablet (Almus Pharmaceuticals Ltd) Diclofenac sodium 25mg Gastro-resistant tablet Oral	10010100
Diclofenac sodium 50mg capsules Diclofenac Sodium	10010100
Diclofenac sodium 25mg tablets Diclofenac Sodium 25mg Tablets Oral	10010100
Voltarol Pain-eze Extra Strength 25mg tablets (Novartis Consumer Health UK Ltd) Diclofenac potassium 25mg Tablet Oral	10010100
DICLOFENAC SODIUM S/R	00000000
Diclofenac 10mg/5ml oral suspension Diclofenac sodium 2mg/1ml Oral suspension Oral	10010100
Dicloflex 50mg Gastro-resistant tablet (Ratiopharm UK Ltd) Diclofenac sodium 50mg Gastro-resistant tablet Oral	10010100
First Resort Double Action Pain Relief 12.5mg tablets (Actavis UK Ltd) Diclofenac potassium 12.5mg Tablet Oral	10010100
Valenac ec 50mg Gastro-resistant tablet (Shire Pharmaceuticals Ltd) Diclofenac sodium 50mg Gastro-resistant tablet Oral	10010100
Diclofenac sodium 25mg gastro-resistant tablets (Sterwin Medicines) Diclofenac sodium 25mg Gastro-resistant tablet Oral	10010100
Diclofenac potassium 50mg tablets (Focus Pharmaceuticals Ltd) Diclofenac potassium 50mg Tablet Oral	10010100
Diclofenac sodium 25mg gastro-resistant tablets (Mylan) Diclofenac sodium 25mg Gastro-resistant tablet Oral	10010100
Voltarol Rapid 50mg tablets (DE Pharmaceuticals) Diclofenac potassium 50mg Tablet Oral	10010100
Dicloflex 25mg gastro-resistant tablets (Almus Pharmaceuticals Ltd) Diclofenac sodium 25mg Gastro-resistant tablet Oral	10010100
Diclofenac sodium 50mg gastro-resistant tablets (A A H Pharmaceuticals Ltd) Diclofenac sodium 50mg Gastro-resistant tablet Oral	10010100
Diclofenac sodium 50mg gastro-resistant tablets (Waymade Healthcare Plc) Diclofenac sodium 50mg Gastro-resistant tablet Oral	10010100
Dicloflex 50mg gastro-resistant tablets (Almus Pharmaceuticals Ltd) Diclofenac sodium 50mg Gastro-resistant tablet Oral	10010100
Diclofenac 50mg Tablet (C P Pharmaceuticals Ltd) Diclofenac sodium 50mg Gastro-resistant tablet Oral	10010100
Diclofenac 25mg Gastro-resistant tablet (Genus Pharmaceuticals Ltd) Diclofenac sodium 25mg Gastro-resistant tablet Oral	10010100
Diclofenac sodium 50mg gastro-resistant tablets (Sterwin Medicines) Diclofenac sodium 50mg Gastro-resistant tablet Oral	10010100
Diclofenac sodium 25mg gastro-resistant tablets (Medreich Plc) Diclofenac sodium 25mg Gastro-resistant tablet Oral	10010100
Diclofenac potassium 50mg tablets Diclofenac potassium 50mg Tablet Oral	10010100
Voltarol Rapid 25mg tablets (Novartis Pharmaceuticals UK Ltd) Diclofenac potassium 25mg Tablet Oral	10010100

Diclofenac sodium 50mg gastro-resistant tablets (Sandoz Ltd) Diclofenac sodium 50mg Gastro-resistant tablet Oral	10010100
Voltarol 50mg dispersible tablets (Waymade Healthcare Plc) Diclofenac sodium 50mg Dispersible tablet Oral	10010100
Diclofenac sodium 25mg gastro-resistant tablets (IVAX Pharmaceuticals UK Ltd) Diclofenac sodium 25mg Gastro-resistant tablet Oral	10010100
Diclofenac potassium 25mg tablets (Accord Healthcare Ltd) Diclofenac potassium 25mg Tablet Oral	10010100
Diclofenac 50mg/5ml oral solution Diclofenac sodium 10mg/1ml Oral solution Oral	10010100
Diclofenac sodium 50mg gastro-resistant tablets (Crescent Pharma Ltd) Diclofenac sodium 50mg Gastro-resistant tablet Oral	10010100
Diclofenac 50mg Tablet (Regent Laboratories Ltd) Diclofenac sodium 50mg Gastro-resistant tablet Oral	10010100
Diclofenac 50mg/5ml oral suspension Diclofenac sodium 10mg/1ml Oral suspension Oral	10010100
Diclofenac potassium 50mg tablets (DE Pharmaceuticals) Diclofenac potassium 50mg Tablet Oral	10010100
Diclofenac 12.5mg/5ml oral solution Diclofenac sodium 2.5mg/1ml Oral solution Oral	10010100
Diclofenac 50mg Gastro-resistant tablet (Pharmacia Ltd) Diclofenac sodium 50mg Gastro-resistant tablet Oral	10010100
Diclofenac potassium 12.5mg tablets Diclofenac potassium 12.5mg Tablet Oral	10010100
Diclofenac sodium 25mg gastro-resistant tablets (Actavis UK Ltd) Diclofenac sodium 25mg Gastro-resistant tablet Oral	10010100
Diclofenac sodium 50mg gastro-resistant tablets (IVAX Pharmaceuticals UK Ltd) Diclofenac sodium 50mg Gastro-resistant tablet Oral	10010100
Voltarol Rapid 50mg tablets (Waymade Healthcare Plc) Diclofenac potassium 50mg Tablet Oral	10010100
Flamrase 50 EC tablets (Teva UK Ltd) Diclofenac sodium 50mg Gastro-resistant tablet Oral	10010100
Diclofenac sodium 50mg gastro-resistant tablets (Teva UK Ltd) Diclofenac sodium 50mg Gastro-resistant tablet Oral	10010100
Diclofenac sodium 50mg gastro-resistant tablets (Medreich Plc) Diclofenac sodium 50mg Gastro-resistant tablet Oral	10010100
Voltarol Rapid 25mg tablets (DE Pharmaceuticals) Diclofenac potassium 25mg Tablet Oral	10010100
Fenactol 50mg gastro-resistant tablets (Dexcel-Pharma Ltd) Diclofenac sodium 50mg Gastro-resistant tablet Oral	10010100
Diclofenac 10mg dispersible tablets Diclofenac sodium 10mg Dispersible tablet Oral	10010100
Valenac ec 25mg Gastro-resistant tablet (Shire Pharmaceuticals Ltd) Diclofenac sodium 25mg Gastro-resistant tablet Oral	10010100
Diclofenac sodium 25mg gastro-resistant tablets (Teva UK Ltd) Diclofenac sodium 25mg Gastro-resistant tablet Oral	10010100
Voltarol 50mg dispersible tablets (Lexon (UK) Ltd) Diclofenac sodium 50mg Dispersible tablet Oral	10010100
Defanac 25mg gastro-resistant tablets (Ranbaxy (UK) Ltd) Diclofenac sodium 25mg Gastro-resistant tablet Oral	10010100
Diclofenac sodium 25mg gastro-resistant tablets Diclofenac Sodium 25mg EC Tablets Oral	10010100
Diclofenac 50mg Gastro-resistant tablet (Almus Pharmaceuticals Ltd) Diclofenac sodium 50mg Gastro-resistant tablet Oral	10010100

Rhumalgan 25mg Tablet (Lagap) Diclofenac sodium 25mg Gastro-resistant tablet Oral	10010100
Dicloflex 50mg gastro-resistant tablets (Dexcel-Pharma Ltd) Diclofenac sodium 50mg Gastro-resistant tablet Oral	10010100
Voltarol Rapid 50mg tablets (Mawdsley-Brooks & Company Ltd) Diclofenac potassium 50mg Tablet Oral	10010100
Diclofenac sodium 25mg gastro-resistant tablets (A A H Pharmaceuticals Ltd) Diclofenac sodium 25mg Gastro-resistant tablet Oral	10010100
Flamrase 25 EC tablets (Teva UK Ltd) Diclofenac sodium 25mg Gastro-resistant tablet Oral	10010100
Volraman 25mg gastro-resistant tablets (LPC Medical (UK) Ltd) Diclofenac sodium 25mg Gastro-resistant tablet Oral	10010100
Diclofenac sodium 50mg gastro-resistant tablets (Sigma Pharmaceuticals Plc) Diclofenac sodium 50mg Gastro-resistant tablet Oral	10010100
Voltarol Rapid 50mg tablets (Sigma Pharmaceuticals Plc) Diclofenac potassium 50mg Tablet Oral	10010100
Fenactol 25mg gastro-resistant tablets (Discovery Pharmaceuticals) Diclofenac sodium 25mg Gastro-resistant tablet Oral	10010100
Voltarol 50mg dispersible tablets (Mawdsley-Brooks & Company Ltd) Diclofenac sodium 50mg Dispersible tablet Oral	10010100
Defanac 50mg gastro-resistant tablets (Ranbaxy (UK) Ltd) Diclofenac sodium 50mg Gastro-resistant tablet Oral	10010100
Voltarol Pain-eze 12.5mg tablets (Novartis Consumer Health UK Ltd) Diclofenac potassium 12.5mg Tablet Oral	10010100
Diclofenac 50mg Tablet (Approved Prescription Services Ltd) Diclofenac sodium 50mg Gastro-resistant tablet Oral	10010100
Isclofen 50mg Gastro-resistant tablet (Isis Products Ltd) Diclofenac sodium 50mg Gastro-resistant tablet Oral	10010100
Diclofenac sodium 25mg gastro-resistant tablets (DE Pharmaceuticals) Diclofenac sodium 25mg Gastro-resistant tablet Oral	10010100
Diclofenac sodium 50mg gastro-resistant tablets (Actavis UK Ltd) Diclofenac sodium 50mg Gastro-resistant tablet Oral	10010100
Diclovol 50mg gastro-resistant tablets (Arun Pharmaceuticals Ltd) Diclofenac sodium 50mg Gastro-resistant tablet Oral	10010100
Dicloflex 25mg gastro-resistant tablets (Teva UK Ltd) Diclofenac sodium 25mg Gastro-resistant tablet Oral	10010100
Diclofenac sodium 50mg gastro-resistant tablets (Genesis Pharmaceuticals Ltd) Diclofenac sodium 50mg Gastro-resistant tablet Oral	10010100
Dicloflex 25mg Gastro-resistant tablet (Ratiopharm UK Ltd) Diclofenac sodium 25mg Gastro-resistant tablet Oral	10010100
Diclofenac potassium 25mg tablets Diclofenac potassium 25mg Tablet Oral	10010100
Diclofenac sodium 25mg gastro-resistant tablets (Kent Pharmaceuticals Ltd) Diclofenac sodium 25mg Gastro-resistant tablet Oral	10010100
Diclofenac 50mg dispersible tablets sugar free Diclofenac sodium 50mg Dispersible tablet Oral	10010100
Diclofenac 25mg Tablet (Berk Pharmaceuticals Ltd) Diclofenac sodium 25mg Gastro-resistant tablet Oral	10010100
Lofensaid 25mg gastro-resistant tablets (Opus Pharmaceuticals Ltd) Diclofenac sodium 25mg Gastro-resistant tablet Oral	10010100
Diclofenac 25mg Gastro-resistant tablet (Pharmacia Ltd) Diclofenac sodium 25mg Gastro-resistant tablet Oral	10010100
Voltarol 50mg dispersible tablets (Novartis Pharmaceuticals UK Ltd) Diclofenac sodium 50mg Dispersible tablet Oral	10010100

Diclofenac potassium 25mg tablets (A A H Pharmaceuticals Ltd) Diclofenac potassium 25mg Tablet Oral	10010100
Diclofenac 50mg dispersible tablets sugar free (Sigma Pharmaceuticals Plc) Diclofenac sodium 50mg Dispersible tablet Oral	10010100
Diclofenac sodium 50mg gastro-resistant tablets (Mylan) Diclofenac sodium 50mg Gastro-resistant tablet Oral	10010100
Rhumalgan 50mg Tablet (Lagap) Diclofenac sodium 50mg Gastro-resistant tablet Oral	10010100
Diclofenac 10mg/5ml oral solution Diclofenac sodium 2mg/1ml Oral solution Oral	10010100
Diclofenac sodium 50mg tablets Diclofenac Sodium 50mg Tablets Oral	10010100
Diclofenac 50mg Tablet (Berk Pharmaceuticals Ltd) Diclofenac sodium 50mg Gastro-resistant tablet Oral	10010100
Diclofenac 25mg Tablet (C P Pharmaceuticals Ltd) Diclofenac sodium 25mg Gastro-resistant tablet Oral	10010100
Acoflam 50mg gastro-resistant tablets (Mercury Pharma Group Ltd) Diclofenac sodium 50mg Gastro-resistant tablet Oral	10010100
Diclofenac potassium 50mg tablets (Accord Healthcare Ltd) Diclofenac potassium 50mg Tablet Oral	10010100
Diclofenac 12.5mg/5ml oral suspension Diclofenac sodium 2.5mg/1ml Oral suspension Oral	10010100
Diclovol 25mg gastro-resistant tablets (Arun Pharmaceuticals Ltd) Diclofenac sodium 25mg Gastro-resistant tablet Oral	10010100
Diclofenac sodium 50mg gastro-resistant tablets (Phoenix Healthcare Distribution Ltd) Diclofenac sodium 50mg Gastro-resistant tablet Oral	10010100
Diclofenac sodium 50mg gastro-resistant tablets (DE Pharmaceuticals) Diclofenac sodium 50mg Gastro-resistant tablet Oral	10010100
Diclofenac sodium 25mg gastro-resistant tablets (Sandoz Ltd) Diclofenac sodium 25mg Gastro-resistant tablet Oral	10010100
Voltarol Rapid 50mg tablets (Stephar (U.K.) Ltd) Diclofenac potassium 50mg Tablet Oral	10010100
Diclozip 25mg gastro-resistant tablets (Ashbourne Pharmaceuticals Ltd) Diclofenac sodium 25mg Gastro-resistant tablet Oral	10010100
Volraman 50mg gastro-resistant tablets (LPC Medical (UK) Ltd) Diclofenac sodium 50mg Gastro-resistant tablet Oral	10010100
Voltarol 50mg dispersible tablets (Sigma Pharmaceuticals Plc) Diclofenac sodium 50mg Dispersible tablet Oral	10010100
Diclozip 50mg gastro-resistant tablets (Ashbourne Pharmaceuticals Ltd) Diclofenac sodium 50mg Gastro-resistant tablet Oral	10010100
Voltarol 50mg dispersible tablets (Stephar (U.K.) Ltd) Diclofenac sodium 50mg Dispersible tablet Oral	10010100
Diclofenac sodium 25mg gastro-resistant tablets Diclofenac sodium 25mg Gastro-resistant tablet Oral	10010100
Diclofenac 15mg/5ml oral suspensionDiclofenac sodium3mg/1mlOral suspensionOral	10010100
Diclofenac potassium 50mg tablets (Medihealth (Northern) Ltd)Diclofenac potassium50mgTabletOral	10010100
Diclofenac sodium 25mg capsulesDiclofenac Sodium	10010100

DESCRIPTION	BNF1
Nurofen Maximum Strength Migraine Pain 684mg caplets (Reckitt Benckiser	4070100

Healthcare (UK) Ltd) Ibuprofen lysine 400mg Tablet Oral	
Galprofen 200mg tablets (Galpharm International Ltd) Ibuprofen 200mg Tablet Oral	10010100
Nurofen Migraine Pain 342mg tablets (Reckitt Benckiser Healthcare (UK) Ltd) Ibuprofen lysine 200mg Tablet Oral	4070100
Galprofen 100mg/5ml oral suspension (Galpharm International Ltd) Ibuprofen 20mg/1ml Oral suspension Oral	10010100
Galprofen Long Lasting 300mg capsules (Galpharm International Ltd) Ibuprofen 300mg Modified-release capsule Oral	10010100
Galprofen Long Lasting 200mg capsules (Galpharm International Ltd) Ibuprofen 200mg Modified-release capsule Oral	10010100

DESCRIPTION	BNF1
Naproxen 500mg tablets Naproxen 500mg Tablet Oral	04070100
Naproxen 250mg tablets Naproxen 250mg Tablet Oral	04070100
Naproxen 500mg gastro-resistant tablets Naproxen 500mg Gastro-resistant tablet Oral	04070100
Naproxen 500mg Tablet (M & A Pharmachem Ltd) Naproxen 500mg Tablet Oral	10010100
Valrox 250mg Tablet (Shire Pharmaceuticals Ltd) Naproxen 250mg Tablet Oral	10010100
Naproxen 250mg gastro-resistant tablets (IVAX Pharmaceuticals UK Ltd) Naproxen 250mg Gastro-resistant tablet Oral	04070100
Naproxen 500mg Gastro-resistant tablet (Almus Pharmaceuticals Ltd) Naproxen 500mg Gastro-resistant tablet Oral	10010100
Naproxen 250mg gastro-resistant tablets (Alliance Healthcare (Distribution) Ltd) Naproxen 250mg Gastro-resistant tablet Oral	04070100
Naproxen 250mg tablets (IVAX Pharmaceuticals UK Ltd) Naproxen 250mg Tablet Oral	04070100
Naproxen 250mg effervescent tablets sugar free Naproxen 250mg Effervescent tablet Oral	10010100
Timpron 250mg Gastro-resistant tablet (Berk Pharmaceuticals Ltd) Naproxen 250mg Gastro-resistant tablet Oral	10010100
Rheuflex 500mg Tablet (Goldshield Pharmaceuticals Ltd) Naproxen 500mg Tablet Oral	10010100
Naproxen 250mg gastro-resistant tablets (Teva UK Ltd) Naproxen 250mg Gastro-resistant tablet Oral	04070100
Stirlescent 250mg effervescent tablets (Stirling Anglian Pharmaceuticals Ltd) Naproxen 250mg Effervescent tablet Oral	10010100
Naprosyn 500mg tablets (Atnahs Pharma UK Ltd) Naproxen 500mg Tablet Oral	04070100
Nycopren 250mg gastro-resistant tablets (Ardern Healthcare Ltd) Naproxen 250mg Gastro-resistant tablet Oral	04070100
Naproxen 250mg tablets (DE Pharmaceuticals) Naproxen 250mg Tablet Oral	04070100
Timpron 500mg Gastro-resistant tablet (Berk Pharmaceuticals Ltd) Naproxen 500mg Gastro-resistant tablet Oral	10010100
Naprosyn EC 250mg tablets (Atnahs Pharma UK Ltd) Naproxen 250mg Gastro-resistant tablet Oral	04070100
Naproxen 500mg tablets (Noumed Life Sciences Ltd) Naproxen 500mg Tablet Oral	04070100

Feminax Ultra 250mg gastro-resistant tablets (Bayer Plc) Naproxen 250mg Gastro-resistant tablet Oral	04070100
Naproxen 250mg gastro-resistant tablets (Ranbaxy (UK) Ltd) Naproxen 250mg Gastro-resistant tablet Oral	04070100
Naproxen 500mg Gastro-resistant tablet (Sterwin Medicines) Naproxen 500mg Gastro-resistant tablet Oral	10010100
Naproxen 500mg tablets (A A H Pharmaceuticals Ltd) Naproxen 500mg Tablet Oral	04070100
Naproxen 250mg tablets (Waymade Healthcare Plc) Naproxen 250mg Tablet Oral	04070100
Timpron 500mg Tablet (Berk Pharmaceuticals Ltd) Naproxen 500mg Tablet Oral	10010100
Naproxen 500mg gastro-resistant tablets (A A H Pharmaceuticals Ltd) Naproxen 500mg Gastro-resistant tablet Oral	04070100
Naproxen 250mg tablets (Teva UK Ltd) Naproxen 250mg Tablet Oral	04070100
Naproxen 500mg tablets (Teva UK Ltd) Naproxen 500mg Tablet Oral	04070100
Naproxen 250mg tablets (Sigma Pharmaceuticals Plc) Naproxen 250mg Tablet Oral	04070100
Naproxen 500mg gastro-resistant tablets (Phoenix Healthcare Distribution Ltd) Naproxen 500mg Gastro-resistant tablet Oral	04070100
Naproxen 250mg tablets (Mylan) Naproxen 250mg Tablet Oral	04070100
Naproxen 250mg/5ml oral suspension Naproxen 50mg/1ml Oral suspension Oral	04070100
Naproxen 500mg tablets (Mylan) Naproxen 500mg Tablet Oral	04070100
Naproxen 500mg Tablet (Almus Pharmaceuticals Ltd) Naproxen 500mg Tablet Oral	10010100
Naprosyn EC 500mg tablets (Atnahs Pharma UK Ltd) Naproxen 500mg Gastro-resistant tablet Oral	04070100
Naproxen 500mg gastro-resistant tablets (DE Pharmaceuticals) Naproxen 500mg Gastro-resistant tablet Oral	04070100
Timpron 250mg Tablet (Berk Pharmaceuticals Ltd) Naproxen 250mg Tablet Oral	10010100
Naproxen 250mg tablets (Genesis Pharmaceuticals Ltd) Naproxen 250mg Tablet Oral	04070100
Naproxen 250mg gastro-resistant tablets (Kent Pharmaceuticals Ltd) Naproxen 250mg Gastro-resistant tablet Oral	04070100
Arthrosin EC 500 tablets (Ashbourne Pharmaceuticals Ltd) Naproxen 500mg Gastro-resistant tablet Oral	04070100
Naproxen 250mg gastro-resistant tablets (Mylan) Naproxen 250mg Gastro-resistant tablet Oral	04070100
Arthrosin EC 250 tablets (Ashbourne Pharmaceuticals Ltd) Naproxen 250mg Gastro-resistant tablet Oral	04070100
Naprosyn 250mg tablets (Atnahs Pharma UK Ltd) Naproxen 250mg Tablet Oral	04070100
Naproxen 250mg gastro-resistant tablets (Genesis Pharmaceuticals Ltd) Naproxen 250mg Gastro-resistant tablet Oral	04070100
Naproxen 500mg gastro-resistant tablets (Genesis Pharmaceuticals Ltd) Naproxen 500mg Gastro-resistant tablet Oral	04070100
NAPROXEN SODIUM	00000000

NAPROXEN 250 MG CAP	00000000
Naproxen Oral solution Naproxen Oral Solution Oral	10010100
Naproxen 500mg tablets (Kent Pharmaceuticals Ltd) Naproxen 500mg Tablet Oral	04070100
Naproxen 500mg tablets (Sigma Pharmaceuticals Plc) Naproxen 500mg Tablet Oral	04070100
Naproxen 500mg tablets (Alliance Healthcare (Distribution) Ltd) Naproxen 500mg Tablet Oral	04070100
Prosaid 250mg Tablet (BHR Pharmaceuticals Ltd) Naproxen 250mg Tablet Oral	10010100
Naproxen 250mg gastro-resistant tablets (Accord Healthcare Ltd) Naproxen 250mg Gastro-resistant tablet Oral	04070100
Naproxen 500mg gastro-resistant tablets (Kent Pharmaceuticals Ltd) Naproxen 500mg Gastro-resistant tablet Oral	04070100
NAPROXEN	00000000
NAPROXEN	00000000
Naproxen 500mg gastro-resistant tablets (Mylan) Naproxen 500mg Gastro-resistant tablet Oral	04070100
Naproxen 250mg tablets (Almus Pharmaceuticals Ltd) Naproxen 250mg Tablet Oral	04070100
Naprosyn S/R 500mg tablets (Roche Products Ltd) Naproxen sodium 500mg Modified-release tablet Oral	10010100
Arthroxen 250mg Tablet (C P Pharmaceuticals Ltd) Naproxen 250mg Tablet Oral	10010100
Naproxen 250mg tablets (Accord Healthcare Ltd) Naproxen 250mg Tablet Oral	04070100
Naproxen 500mg gastro-resistant tablets (Actavis UK Ltd) Naproxen 500mg Gastro-resistant tablet Oral	04070100
Naproxen 250mg gastro-resistant tablets (Sovereign Medical Ltd) Naproxen 250mg Gastro-resistant tablet Oral	04070100
Rheuflex 250mg Tablet (Goldshield Pharmaceuticals Ltd) Naproxen 250mg Tablet Oral	10010100
Naproxen 500mg gastro-resistant tablets (Alliance Healthcare (Distribution) Ltd) Naproxen 500mg Gastro-resistant tablet Oral	04070100
Naproxen 250mg tablets (Crescent Pharma Ltd) Naproxen 250mg Tablet Oral	04070100
Naproxen 500mg tablets (Wockhardt UK Ltd) Naproxen 500mg Tablet Oral	04070100
Boots Period Pain Relief 250mg gastro-resistant tablets (The Boots Company Plc) Naproxen 250mg Gastro-resistant tablet Oral	04070100
Naproxen 250mg tablets (Milpharm Ltd) Naproxen 250mg Tablet Oral	04070100
Naproxen 250mg tablets (Wockhardt UK Ltd) Naproxen 250mg Tablet Oral	04070100
Prosaid 500mg Tablet (BHR Pharmaceuticals Ltd) Naproxen 500mg Tablet Oral	10010100
Naproxen 500mg tablets (Pfizer Ltd) Naproxen 500mg Tablet Oral	04070100
Pranoxen continus 500mg Tablet (Napp Pharmaceuticals Ltd) Naproxen sodium 500mg Modified-release tablet Oral	10010100

Arthrosin 250 tablets (Ashbourne Pharmaceuticals Ltd) Naproxen 250mg Tablet Oral	04070100
Naproxen 500mg tablets (Accord Healthcare Ltd) Naproxen 500mg Tablet Oral	04070100
Naproxen 500mg gastro-resistant tablets (Teva UK Ltd) Naproxen 500mg Gastro-resistant tablet Oral	04070100
Naproxen 500mg Tablet (Berk Pharmaceuticals Ltd) Naproxen 500mg Tablet Oral	10010100
Naproxen 250mg Gastro-resistant tablet (Almus Pharmaceuticals Ltd) Naproxen 250mg Gastro-resistant tablet Oral	10010100
Naproxen 250mg gastro-resistant tablets Naproxen 250mg Gastro-resistant tablet Oral	04070100
Naproxen 250mg gastro-resistant tablets (A A H Pharmaceuticals Ltd) Naproxen 250mg Gastro-resistant tablet Oral	04070100
Naproxen 500mg Gastro-resistant tablet (Galen Ltd) Naproxen 500mg Gastro-resistant tablet Oral	10010100
Naproxen 250mg tablets (Phoenix Healthcare Distribution Ltd) Naproxen 250mg Tablet Oral	04070100
Naproxen 250mg tablets (A A H Pharmaceuticals Ltd) Naproxen 250mg Tablet Oral	04070100
Naproxen 250mg Gastro-resistant tablet (Galen Ltd) Naproxen 250mg Gastro-resistant tablet Oral	10010100
Naproxen 250mg gastro-resistant tablets (Sigma Pharmaceuticals Plc) Naproxen 250mg Gastro-resistant tablet Oral	04070100
Naproxen 500mg tablets (Milpharm Ltd) Naproxen 500mg Tablet Oral	04070100
Nycopren 500mg gastro-resistant tablets (Ardern Healthcare Ltd) Naproxen 500mg Gastro-resistant tablet Oral	04070100
Naproxen 250mg Tablet (Berk Pharmaceuticals Ltd) Naproxen 250mg Tablet Oral	10010100
Naproxen 500mg gastro-resistant tablets (Ranbaxy (UK) Ltd) Naproxen 500mg Gastro-resistant tablet Oral	04070100
Naproxen 250mg tablets (Kent Pharmaceuticals Ltd) Naproxen 250mg Tablet Oral	04070100
Naproxen 500mg gastro-resistant tablets (Sigma Pharmaceuticals Plc) Naproxen 500mg Gastro-resistant tablet Oral	04070100
Naproxen 500mg gastro-resistant tablets (Waymade Healthcare Plc) Naproxen 500mg Gastro-resistant tablet Oral	04070100
Naproxen 500mg gastro-resistant tablets (Almus Pharmaceuticals Ltd) Naproxen 500mg Gastro-resistant tablet Oral	04070100
Naproxen 500mg gastro-resistant tablets (IVAX Pharmaceuticals UK Ltd) Naproxen 500mg Gastro-resistant tablet Oral	04070100
Valrox 500mg Tablet (Shire Pharmaceuticals Ltd) Naproxen 500mg Tablet Oral	10010100
Naproxen 250mg tablets (Actavis UK Ltd) Naproxen 250mg Tablet Oral	04070100
Naproxen 500mg tablets (DE Pharmaceuticals) Naproxen 500mg Tablet Oral	04070100
Naproxen 250mg gastro-resistant tablets (Phoenix Healthcare Distribution Ltd) Naproxen 250mg Gastro-resistant tablet Oral	04070100
Naproxen 500mg Granules Naproxen 500mg Granules Oral	10010100
Arthroxen 500mg Tablet (C P Pharmaceuticals Ltd) Naproxen 500mg Tablet Oral	10010100

Naproxen 500mg tablets (Actavis UK Ltd) Naproxen 500mg Tablet Oral	04070100
Naprosyn 500mg Granules (Roche Products Ltd) Naproxen 500mg Granules Oral	10010100
Naproxen 250mg tablets (Mawdsley-Brooks & Company Ltd) Naproxen 250mg Tablet Oral	04070100
Naproxen 250mg gastro-resistant tablets (DE Pharmaceuticals) Naproxen 250mg Gastro-resistant tablet Oral	04070100
Arthrosin 500 tablets (Ashbourne Pharmaceuticals Ltd) Naproxen 500mg Tablet Oral	04070100
Naproxen 250mg gastro-resistant tablets (Almus Pharmaceuticals Ltd) Naproxen 250mg Gastro-resistant tablet Oral	04070100
Naproxen 500mg modified-release tablets Naproxen sodium 500mg Modified-release tablet Oral	00000000
Naproxen 250mg tablets (Alliance Healthcare (Distribution) Ltd) Naproxen 250mg Tablet Oral	04070100
Naproxen 500mg gastro-resistant tablets (Medreich Plc)Naproxen500mgGastro-resistant tabletOral	04070100
Naproxen 500mg tablets (Almus Pharmaceuticals Ltd)Naproxen500mgTabletOral	04070100
Naproxen 500mg tablets (Bristol Laboratories Ltd)Naproxen500mgTabletOral	04070100

DESCRIPTION	BNF1
Mefenamic acid 500mg tablets Mefenamic acid 500mg Tablet Oral	10010100
Ponstan Forte 500mg tablets (Chemidex Pharma Ltd) Mefenamic acid 500mg Tablet Oral	10010100
Mefenamic acid 500mg tablets (Alliance Healthcare (Distribution) Ltd) Mefenamic acid 500mg Tablet Oral	10010100
Mefenamic acid 250mg Capsule (Teva UK Ltd) Mefenamic acid 250mg Capsule Oral	10010100
PONSTAN	00000000
Opustan 250mg Capsule (Opus Pharmaceuticals Ltd) Mefenamic acid 250mg Capsule Oral	10010100
Mefenamic acid 250mg Capsule (Sandoz Ltd) Mefenamic acid 250mg Capsule Oral	10010100
Mefenamic acid 500mg tablets (Zentiva) Mefenamic acid 500mg Tablet Oral	10010100
Mefenamic acid 250mg capsules (Essential Generics Ltd) Mefenamic acid 250mg Capsule Oral	10010100
Mefenamic acid 500mg tablets (Actavis UK Ltd) Mefenamic acid 500mg Tablet Oral	10010100
Mefenamic acid 500mg tablets (A A H Pharmaceuticals Ltd) Mefenamic acid 500mg Tablet Oral	10010100
Mefenamic acid 250mg Capsule (Berk Pharmaceuticals Ltd) Mefenamic acid 250mg Capsule Oral	10010100
Dysman 500 tablets (Ashbourne Pharmaceuticals Ltd) Mefenamic acid 500mg Tablet Oral	10010100
Mendys 250mg Capsule (Kent Pharmaceuticals Ltd) Mefenamic acid 250mg Capsule Oral	10010100
MEFENAMIC ACID DISPERSIBLE	0000000

	0
PONSTAN FORTE	0000000 0
Mefenamic acid 250mg capsules Mefenamic acid 250mg Capsule Oral	1001010 0
Mefenamic acid 500mg tablets (Sigma Pharmaceuticals Plc) Mefenamic acid 500mg Tablet Oral	1001010 0
Ponstan 50mg/5ml paediatric Liquid (Chemidex Pharma Ltd) Mefenamic acid 10mg/1ml Oral suspension Oral	1001010 0
Meflam 500mg Tablet (Trinity Pharmaceuticals Ltd) Mefenamic acid 500mg Tablet Oral	1001010 0
Mefenamic acid 500mg Tablet (Berk Pharmaceuticals Ltd) Mefenamic acid 500mg Tablet Oral	1001010 0
Mefenamic acid 250mg Dispersible tablet Mefenamic Acid 250mg Dispersible Tablet Oral	1001010 0
Ponstan 250mg Dispersible tablet (Chemidex Pharma Ltd) Mefenamic Acid 250mg Dispersible Tablet Oral	1001010 0
Mefenamic acid 250mg/5ml oral suspension Mefenamic acid 50mg/1ml Oral suspension Oral	1001010 0
Opustan 500mg Tablet (Opus Pharmaceuticals Ltd) Mefenamic acid 500mg Tablet Oral	1001010 0
Meflam 250mg Capsule (Trinity Pharmaceuticals Ltd) Mefenamic acid 250mg Capsule Oral	1001010 0
Mefenamic acid 250mg capsules (Advanz Pharma) Mefenamic acid 250mg Capsule Oral	1001010 0
Mefenamic acid 250mg Capsule (Actavis UK Ltd) Mefenamic acid 250mg Capsule Oral	1001010 0
Dysman 250 capsules (Ashbourne Pharmaceuticals Ltd) Mefenamic acid 250mg Capsule Oral	1001010 0
Mefenamic acid 250mg capsules (Mylan) Mefenamic acid 250mg Capsule Oral	1001010 0
Mefenamic acid 500mg/5ml oral suspension Mefenamic acid 100mg/1ml Oral suspension Oral	1001010 0
Mefenamic acid 500mg tablets (IVAX Pharmaceuticals UK Ltd) Mefenamic acid 500mg Tablet Oral	1001010 0
Mefenamic acid 50mg/5ml oral suspension Mefenamic acid 10mg/1ml Oral suspension Oral	1001010 0
Mefenamic acid 500mg tablets (Almus Pharmaceuticals Ltd) Mefenamic acid 500mg Tablet Oral	1001010 0
Dysman 250mg Capsule (Ashbourne Pharmaceuticals Ltd) Mefenamic acid 250mg Capsule Oral	1001010 0
Contraflam 250mg Capsule (Berk Pharmaceuticals Ltd) Mefenamic acid 250mg Capsule Oral	1001010 0
Mefenamic acid 250mg capsules (Zentiva) Mefenamic acid 250mg Capsule Oral	1001010 0
Mefenamic acid 500mg tablets (Teva UK Ltd) Mefenamic acid 500mg Tablet Oral	1001010 0
Mefenamic acid 250mg capsules (A A H Pharmaceuticals Ltd) Mefenamic acid 250mg Capsule Oral	1001010 0
Mefenamic acid 500mg tablets (Waymade Healthcare Plc) Mefenamic acid 500mg Tablet Oral	1001010 0
Mefenamic acid 250mg Capsule (IVAX Pharmaceuticals UK Ltd) Mefenamic acid 250mg Capsule Oral	1001010 0

Ponstan 250mg capsules (Chemidex Pharma Ltd) Mefenamic acid 250mg Capsule Oral	1001010 0
Contraflam 500mg Tablet (Berk Pharmaceuticals Ltd) Mefenamic acid 500mg Tablet Oral	1001010 0
Mefenamic acid 250mg capsules (Waymade Healthcare Plc) Mefenamic acid 250mg Capsule Oral	1001010 0
Mefenamic acid 500mg tablets (Essential Generics Ltd) Mefenamic acid 500mg Tablet Oral	1001010 0
Mefenamic acid 250mg capsules (Alliance Healthcare (Distribution) Ltd) Mefenamic acid 250mg Capsule Oral	1001010 0

DESCRIPTION	BNF1
Tolfenamic acid 200mg Capsule Tolfenamic Acid 200mg Capsule Oral	0407040 1
Clotam Rapid 200mg tablets (Galen Ltd) Tolfenamic acid 200mg Tablet Oral	0407040 1
Tolfenamic acid 200mg tablets (A A H Pharmaceuticals Ltd) Tolfenamic acid 200mg Tablet Oral	0407040 1
Tolfenamic acid 200mg tablets Tolfenamic acid 200mg Tablet Oral	0407040 1
Clotam 200mg Capsule (Thames Laboratories Ltd) Tolfenamic Acid 200mg Capsule Oral	0407040 1

DESCRIPTION	BNF1
Atenolol 50mg tablets Atenolol 50mg Tablet Oral	0204000 0
Atenolol 100mg tablets Atenolol 100mg Tablet Oral	0204000 0
Propranolol 80mg modified-release capsules Propranolol hydrochloride 80mg Modified-release capsule Oral	0204000 0
Half Inderal LA 80mg capsules (AstraZeneca UK Ltd) Propranolol hydrochloride 80mg Modified-release capsule Oral	0204000 0
Propranolol 160mg modified-release capsules Propranolol hydrochloride 160mg Modified-release capsule Oral	0204000 0
Tenormin LS 50mg tablets (AstraZeneca UK Ltd) Atenolol 50mg Tablet Oral	0204000 0
Tenormin 100mg tablets (AstraZeneca UK Ltd) Atenolol 100mg Tablet Oral	0204000 0
Propranolol 80mg tablets Propranolol hydrochloride 80mg Tablet Oral	0204000 0
Inderal LA 160mg capsules (AstraZeneca UK Ltd) Propranolol hydrochloride 160mg Modified-release capsule Oral	0204000 0
Metoprolol 100mg tablets Metoprolol tartrate 100mg Tablet Oral	0204000 0
Propranolol 160mg tablets Propranolol hydrochloride 160mg Tablet Oral	0204000 0
Betaloc-SA 200mg tablets (AstraZeneca UK Ltd) Metoprolol tartrate 200mg Modified-release tablet Oral	0204000 0
Inderal 80mg tablets (AstraZeneca UK Ltd) Propranolol hydrochloride 80mg Tablet Oral	0204000 0
Betaloc 100mg tablets (AstraZeneca UK Ltd) Metoprolol tartrate 100mg Tablet Oral	0204000 0

Corgard 40mg tablets (Sanofi-Synthelabo Ltd) Nadolol 40mg Tablet Oral	02040000
Rapranol SR 80mg capsules (Ranbaxy (UK) Ltd) Propranolol hydrochloride 80mg Modified-release capsule Oral	02040000
Inderal LA 160mg capsules (Waymade Healthcare Plc) Propranolol hydrochloride 160mg Modified-release capsule Oral	02040000
Propranolol LA 80mg Modified-release capsule (Approved Prescription Services Ltd) Propranolol hydrochloride 80mg Modified-release capsule Oral	02040000
Atenolol 50mg tablets (Mylan) Atenolol 50mg Tablet Oral	02040000
Bedranol SR 160mg capsules (Sandoz Ltd) Propranolol hydrochloride 160mg Modified-release capsule Oral	02040000
PROPRANOLOL S/R	00000000
Berkolol 160mg Tablet (Berk Pharmaceuticals Ltd) Propranolol hydrochloride 160mg Tablet Oral	02030202
Atenolol 50mg tablets (Crescent Pharma Ltd) Atenolol 50mg Tablet Oral	02040000
Nadolol 40mg/5ml oral solution Nadolol 8mg/1ml Oral solution Oral	02040000
Bedranol 80mg tablets (Ennogen Pharma Ltd) Propranolol hydrochloride 80mg Tablet Oral	02040000
Metoprolol 100mg tablets (IVAX Pharmaceuticals UK Ltd) Metoprolol tartrate 100mg Tablet Oral	02040000
Metoprolol tartrate Oral solution Metoprolol Tartrate Oral Solution Oral	02040000
Corgard 80mg tablets (Sanofi) Nadolol 80mg Tablet Oral	02040000
Beta-Prograne 160mg modified-release capsules (Teva UK Ltd) Propranolol hydrochloride 160mg Modified-release capsule Oral	02040000
Atenolol 50mg tablets (Sandoz Ltd) Atenolol 50mg Tablet Oral	02040000
Betim 10mg Tablet (ICN Pharmaceuticals France S.A.) Timolol maleate 10mg Tablet Oral	02040000
Atenolol 50mg tablets (Alliance Healthcare (Distribution) Ltd) Atenolol 50mg Tablet Oral	02040000
Propranolol 80mg modified-release capsules (Mawdsley-Brooks & Company Ltd) Propranolol hydrochloride 80mg Modified-release capsule Oral	02040000
Atenolol 50mg tablets (Actavis UK Ltd) Atenolol 50mg Tablet Oral	02040000
Propranolol 80mg/5ml oral solution Propranolol Hydrochloride 80mg/5ml Oral Solution Oral	02030202
Atenamin 50mg Tablet (OPD Pharm) Atenolol 50mg Tablet Oral	02040000
Propranolol 160mg modified-release capsules (Teva UK Ltd) Propranolol hydrochloride 160mg Modified-release capsule Oral	02040000
Vasaten 50mg Tablet (Shire Pharmaceuticals Ltd) Atenolol 50mg Tablet Oral	02040000
Atenolol 100mg tablets (Kent Pharmaceuticals Ltd) Atenolol 100mg Tablet Oral	02040000
Atenolol 100mg tablets (A A H Pharmaceuticals Ltd) Atenolol 100mg Tablet Oral	02040000
Atenix 50 tablets (Ashbourne Pharmaceuticals Ltd) Atenolol 50mg Tablet Oral	02040000

Lopresor SR 200mg tablets (Recordati Pharmaceuticals Ltd) Metoprolol tartrate 200mg Modified-release tablet Oral	02040000
Half Inderal LA 80mg capsules (Necessity Supplies Ltd) Propranolol hydrochloride 80mg Modified-release capsule Oral	02040000
Propranolol SR 160mg Modified-release capsule (C P Pharmaceuticals Ltd) Propranolol hydrochloride 160mg Modified-release capsule Oral	02040000
Propanix 160mg Tablet (Ashbourne Pharmaceuticals Ltd) Propranolol hydrochloride 160mg Tablet Oral	02030202
Propranolol SR 160mg Modified-release capsule (Hillcross Pharmaceuticals Ltd) Propranolol hydrochloride 160mg Modified-release capsule Oral	02040000
Propranolol 80mg tablets (Actavis UK Ltd) Propranolol hydrochloride 80mg Tablet Oral	02040000
Atenolol 50mg tablets (Teva UK Ltd) Atenolol 50mg Tablet Oral	02040000
Propranolol 160mg tablets (Actavis UK Ltd) Propranolol hydrochloride 160mg Tablet Oral	02040000
Angilol 80mg Tablet (DDSA Pharmaceuticals Ltd) Propranolol hydrochloride 80mg Tablet Oral	02030202
Slo-Pro 160mg capsules (Mylan) Propranolol hydrochloride 160mg Modified-release capsule Oral	02040000
Atenolol 50mg tablets (DE Pharmaceuticals) Atenolol 50mg Tablet Oral	02040000
Atenolol 50mg tablets (Boston Healthcare Ltd) Atenolol 50mg Tablet Oral	02040000
Propranolol 80mg Capsule (IVAX Pharmaceuticals UK Ltd) Propranolol hydrochloride 80mg Modified-release capsule Oral	02040000
Atenolol 100mg Tablet (Celltech Pharma Europe Ltd) Atenolol 100mg Tablet Oral	02040000
Metoprolol 200mg modified-release tablets Metoprolol tartrate 200mg Modified-release tablet Oral	00000000
Propranolol LA 160mg Capsule (Approved Prescription Services Ltd) Propranolol hydrochloride 160mg Modified-release capsule Oral	02040000
Nadolol 40mg tablets Nadolol 40mg Tablet Oral	02040000
Half Beta-Prograne 80mg modified-release capsules (Actavis UK Ltd) Propranolol hydrochloride 80mg Modified-release capsule Oral	02040000
Propranolol 80mg Modified-release capsule (Actavis UK Ltd) Propranolol hydrochloride 80mg Modified-release capsule Oral	02040000
Probeta LA 160mg Capsule (Trinity Pharmaceuticals Ltd) Propranolol hydrochloride 160mg Modified-release capsule Oral	02040000
Atenix 100 tablets (Ashbourne Pharmaceuticals Ltd) Atenolol 100mg Tablet Oral	02040000
Apsolol 160mg Tablet (Approved Prescription Services Ltd) Propranolol hydrochloride 160mg Tablet Oral	02030202
Bedranol SR 80mg capsules (Sandoz Ltd) Propranolol hydrochloride 80mg Modified-release capsule Oral	02040000
Betadur cr 160mg Modified-release capsule (Monmouth Pharmaceuticals Ltd) Propranolol hydrochloride 160mg Modified-release capsule Oral	02040000
Propranolol 160mg modified-release capsules (A A H Pharmaceuticals Ltd) Propranolol hydrochloride 160mg Modified-release capsule Oral	02040000
BEDRANOL SR 80 MG CAP	00000000
Lopranol la 160mg Capsule (Opus Pharmaceuticals Ltd) Propranolol hydrochloride 160mg Modified-release capsule Oral	02040000

Atenolol 100mg tablets (Almus Pharmaceuticals Ltd) Atenolol 100mg Tablet Oral	02040000
Propranolol 80mg tablets (Ranbaxy (UK) Ltd) Propranolol hydrochloride 80mg Tablet Oral	02040000
Bedranol SR 80mg capsules (Almus Pharmaceuticals Ltd) Propranolol hydrochloride 80mg Modified-release capsule Oral	00000000
Atenolol 50mg tablets (A A H Pharmaceuticals Ltd) Atenolol 50mg Tablet Oral	02040000
Blocadren 10mg Tablet (Merck Sharp & Dohme Ltd) Timolol maleate 10mg Tablet Oral	02040000
Timolol 10mg tablets Timolol maleate 10mg Tablet Oral	02040000
Berkolol 80mg Tablet (Berk Pharmaceuticals Ltd) Propranolol hydrochloride 80mg Tablet Oral	02030202
Propranolol 80mg tablets (Mylan) Propranolol hydrochloride 80mg Tablet Oral	02040000
Propanix 160mg Modified-release capsule (Ashbourne Pharmaceuticals Ltd) Propranolol hydrochloride 160mg Modified-release capsule Oral	02040000
Atenolol 100mg tablets (Wockhardt UK Ltd) Atenolol 100mg Tablet Oral	02040000
Nadolol 40mg/5ml oral suspension Nadolol 8mg/1ml Oral suspension Oral	02040000
Atenolol 50mg tablets (Zentiva) Atenolol 50mg Tablet Oral	02040000
Mepranix 100mg Tablet (Ashbourne Pharmaceuticals Ltd) Metoprolol tartrate 100mg Tablet Oral	02040000
Propranolol 80mg Modified-release capsule (Lagap) Propranolol hydrochloride 80mg Modified-release capsule Oral	02040000
Half Beta-Prograne 80mg modified-release capsules (Actavis UK Ltd) Propranolol hydrochloride 80mg Modified-release capsule Oral	00000000
Propranolol 160mg tablets (Mylan) Propranolol hydrochloride 160mg Tablet Oral	02040000
Atenolol 50mg tablets (Phoenix Healthcare Distribution Ltd) Atenolol 50mg Tablet Oral	02040000
Propranolol 160mg Modified-release capsule (Actavis UK Ltd) Propranolol hydrochloride 160mg Modified-release capsule Oral	02040000
Atenolol 50mg tablets (Kent Pharmaceuticals Ltd) Atenolol 50mg Tablet Oral	02040000
Atenolol 100mg tablets (Sandoz Ltd) Atenolol 100mg Tablet Oral	02040000
Half-betadur cr 80mg Capsule (Monmouth Pharmaceuticals Ltd) Propranolol hydrochloride 80mg Modified-release capsule Oral	02040000
Metoprolol 100mg tablets (Waymade Healthcare Plc) Metoprolol tartrate 100mg Tablet Oral	02040000
Half Beta-Prograne 80mg modified-release capsules (Teva UK Ltd) Propranolol hydrochloride 80mg Modified-release capsule Oral	02040000
Metoprolol 100mg tablets (Alliance Healthcare (Distribution) Ltd) Metoprolol tartrate 100mg Tablet Oral	02040000
Propranolol 80mg tablets (Teva UK Ltd) Propranolol hydrochloride 80mg Tablet Oral	02040000
Antipressan 100mg tablets (Teva UK Ltd) Atenolol 100mg Tablet Oral	02040000
Antipressan 50mg tablets (Teva UK Ltd) Atenolol 50mg Tablet Oral	02040000

ATENOLOL	0000000<br>0
Atenolol 50mg tablets (Tillomed Laboratories Ltd) Atenolol 50mg Tablet Oral	0204000<br>0
Atenolol 50mg Tablet (Celltech Pharma Europe Ltd) Atenolol 50mg Tablet Oral	0204000<br>0
Propranolol 160mg Modified-release capsule (Sandoz Ltd) Propranolol hydrochloride 160mg Modified-release capsule Oral	0204000<br>0
HALF-INDERAL LA	0000000<br>0
Bedranol SR 160mg capsules (Almus Pharmaceuticals Ltd) Propranolol hydrochloride 160mg Modified-release capsule Oral	0204000<br>0
Inderal 160mg Tablet (AstraZeneca UK Ltd) Propranolol hydrochloride 160mg Tablet Oral	0203020<br>2
Half Beta-Prograne 80mg modified-release capsules (Tillomed Laboratories Ltd) Propranolol hydrochloride 80mg Modified-release capsule Oral	0204000<br>0
Rapranol SR 160mg capsules (Ranbaxy (UK) Ltd) Propranolol hydrochloride 160mg Modified-release capsule Oral	0204000<br>0
METOPROLOL FUMARATE 190 MG TAB	0000000<br>0
Atenolol 50mg tablets (Almus Pharmaceuticals Ltd) Atenolol 50mg Tablet Oral	0204000<br>0
Atenolol 50mg tablets (IVAX Pharmaceuticals UK Ltd) Atenolol 50mg Tablet Oral	0204000<br>0
Beta-Prograne 160mg modified-release capsules (Tillomed Laboratories Ltd) Propranolol hydrochloride 160mg Modified-release capsule Oral	0204000<br>0
Nadolol Oral solution Nadolol Oral Solution	0204000<br>0
Atenolol 50mg tablets (Accord Healthcare Ltd) Atenolol 50mg Tablet Oral	0204000<br>0
Propranolol SR 80mg Modified-release capsule (C P Pharmaceuticals Ltd) Propranolol hydrochloride 80mg Modified-release capsule Oral	0204000<br>0
Propanix 80mg Tablet (Ashbourne Pharmaceuticals Ltd) Propranolol hydrochloride 80mg Tablet Oral	0203020<br>2
Beta-Prograne 160mg modified-release capsules (Actavis UK Ltd) Propranolol hydrochloride 160mg Modified-release capsule Oral	0204000<br>0
Atenolol 50mg tablets (Wockhardt UK Ltd) Atenolol 50mg Tablet Oral	0204000<br>0
Propranolol 80mg modified-release capsules (Teva UK Ltd) Propranolol hydrochloride 80mg Modified-release capsule Oral	0204000<br>0
ATENOLOL	0000000<br>0
Propranolol 80mg modified-release capsules (DE Pharmaceuticals) Propranolol hydrochloride 80mg Modified-release capsule Oral	0204000<br>0
Metoprolol 100mg tablets (Actavis UK Ltd) Metoprolol tartrate 100mg Tablet Oral	0204000<br>0
Betim 10mg tablets (Meda Pharmaceuticals Ltd) Timolol maleate 10mg Tablet Oral	0204000<br>0
Sloprolol 160mg Capsule (C P Pharmaceuticals Ltd) Propranolol hydrochloride 160mg Modified-release capsule Oral	0204000<br>0
Propranolol 80mg modified-release capsules (Kent Pharmaceuticals Ltd) Propranolol hydrochloride 80mg Modified-release capsule Oral	0204000<br>0
Atenamin 100mg Tablet (OPD Pharm) Atenolol 100mg Tablet Oral	0204000<br>0

Metoprolol 100mg tablets (Teva UK Ltd) Metoprolol tartrate 100mg Tablet Oral	02040000
Half Inderal LA 80mg capsules (DE Pharmaceuticals) Propranolol hydrochloride 80mg Modified-release capsule Oral	02040000
Atenolol 50mg Tablet (Berk Pharmaceuticals Ltd) Atenolol 50mg Tablet Oral	02040000
Corgard 80mg tablets (Lexon (UK) Ltd) Nadolol 80mg Tablet Oral	02040000
Propranolol oral solution Propranolol Hydrochloride	02030202
Half propanix la 80mg Modified-release capsule (Ashbourne Pharmaceuticals Ltd) Propranolol hydrochloride 80mg Modified-release capsule Oral	02040000
Nadolol 80mg tablets Nadolol 80mg Tablet Oral	02040000
Lopresor 100mg tablets (Recordati Pharmaceuticals Ltd) Metoprolol tartrate 100mg Tablet Oral	02040000
Atenolol 100mg tablets (Teva UK Ltd) Atenolol 100mg Tablet Oral	02040000
Bedranol SR 80mg capsules (Almus Pharmaceuticals Ltd) Propranolol hydrochloride 80mg Modified-release capsule Oral	02040000
Atenolol 50mg tablets (Sigma Pharmaceuticals Plc) Atenolol 50mg Tablet Oral	02040000
Propranolol 80mg modified-release capsules (A A H Pharmaceuticals Ltd) Propranolol hydrochloride 80mg Modified-release capsule Oral	02040000
Atenolol 100mg tablets (Mylan) Atenolol 100mg Tablet Oral	02040000
Metoprolol 100mg tablets (A A H Pharmaceuticals Ltd) Metoprolol tartrate 100mg Tablet Oral	02040000
Propanix LA 160mg Modified-release capsule (Ashbourne Pharmaceuticals Ltd) Propranolol hydrochloride 160mg Modified-release capsule Oral	02040000
Propranolol 160mg Modified-release capsule (Lagap) Propranolol hydrochloride 160mg Modified-release capsule Oral	02040000
Totamol 50mg Tablet (C P Pharmaceuticals Ltd) Atenolol 50mg Tablet Oral	02040000
Bedranol sr 160mg Capsule (Lagap) Propranolol hydrochloride 160mg Modified-release capsule Oral	02040000
Atenolol 100mg tablets (Phoenix Healthcare Distribution Ltd) Atenolol 100mg Tablet Oral	02040000
Propranolol 80mg tablets (A A H Pharmaceuticals Ltd) Propranolol hydrochloride 80mg Tablet Oral	02040000
Inderal LA 160mg capsules (Sigma Pharmaceuticals Plc) Propranolol hydrochloride 160mg Modified-release capsule Oral	02040000
Atenolol 100mg tablets (Crescent Pharma Ltd) Atenolol 100mg Tablet Oral	02040000
Atenolol 100mg tablets (IVAX Pharmaceuticals UK Ltd) Atenolol 100mg Tablet Oral	02040000
Atenolol 50mg tablets (Strides Pharma UK Ltd) Atenolol 50mg Tablet Oral	02040000
Totamol 100mg Tablet (C P Pharmaceuticals Ltd) Atenolol 100mg Tablet Oral	02040000
Propranolol 160mg tablets (DE Pharmaceuticals) Propranolol hydrochloride 160mg Tablet Oral	02040000
Metoprolol 100mg tablets (Mylan) Metoprolol tartrate 100mg Tablet Oral	02040000

Propranolol 80mg modified-release capsules (Waymade Healthcare Plc) Propranolol hydrochloride 80mg Modified-release capsule Oral	02040000
Propranolol 160mg Capsule (IVAX Pharmaceuticals UK Ltd) Propranolol hydrochloride 160mg Modified-release capsule Oral	02040000
Atenolol 100mg tablets (Actavis UK Ltd) Atenolol 100mg Tablet Oral	02040000
Atenolol 50mg tablets (Waymade Healthcare Plc) Atenolol 50mg Tablet Oral	02040000
Nadolol 80mg/5ml oral suspension Nadolol 16mg/1ml Oral suspension Oral	02040000
Half propatard la 80mg Modified-release capsule (Galen Ltd) Propranolol hydrochloride 80mg Modified-release capsule Oral	02040000
Lopresor 100mg Tablet (Novartis Pharmaceuticals UK Ltd) Metoprolol tartrate 100mg Tablet Oral	02040000
Atenolol 50mg tablets (Bristol Laboratories Ltd) Atenolol 50mg Tablet Oral	02040000
Propranolol 80mg tablets (Alliance Healthcare (Distribution) Ltd)Propranolol hydrochloride80mgTabletOral	02040000
Propranolol 80mg Tablet (Celltech Pharma Europe Ltd)Propranolol hydrochloride80mgTabletOral	02030202
Inderal LA 160mg capsules (DE Pharmaceuticals)Propranolol hydrochloride160mgModified-release capsuleOral	02040000
Half Inderal LA 80mg capsules (Waymade Healthcare Plc)Propranolol hydrochloride80mgModified-release capsuleOral	02040000
Propranolol 80mg tablets (Relonchem Ltd)Propranolol hydrochloride80mgTabletOral	02040000
INDERAL	00000000

DESCRIPTION	BNF1
Aimovig 70mg/1ml solution for injection pre-filled pens (Novartis Pharmaceuticals UK Ltd) Erenumab 70mg/1ml Solution for injection Subcutaneous	04070402
Aimovig 140mg/1ml solution for injection pre-filled pens (Novartis Pharmaceuticals UK Ltd) Erenumab 140mg/1ml Solution for injection Subcutaneous	04070402
Erenumab 70mg/1ml solution for injection pre-filled disposable devicesErenumab70mg/1mlSolution for injectionSubcutaneous	04070402
Ajovy 225mg/1.5ml solution for injection pre-filled syringes (Teva UK Ltd)Fremanezumab150mg/1mlSolution for injectionSubcutaneous	04070402

DESCRIPTION	BNF1
Candesartan 16mg tablets (Consilient Health Ltd) Candesartan cilexetil 16mg Tablet Oral	02050502
Candesartan 16mg tablets (Tillomed Laboratories Ltd) Candesartan cilexetil 16mg Tablet Oral	02050502
Candesartan 16mg tablets (Mylan) Candesartan cilexetil 16mg Tablet Oral	02050502
Candesartan 16mg tablets (A A H Pharmaceuticals Ltd) Candesartan cilexetil 16mg Tablet Oral	02050502

Amias 16mg tablets (Takeda UK Ltd) Candesartan cilexetil 16mg Tablet Oral	02050502
Candesartan 16mg tablets (Mawdsley-Brooks & Company Ltd) Candesartan cilexetil 16mg Tablet Oral	02050502
Candesartan 16mg tablets (Teva UK Ltd) Candesartan cilexetil 16mg Tablet Oral	02050502
Candesartan 16mg tablets (Alliance Healthcare (Distribution) Ltd) Candesartan cilexetil 16mg Tablet Oral	02050502
Candesartan 16mg tablets (Actavis UK Ltd) Candesartan cilexetil 16mg Tablet Oral	02050502
Candesartan 16mg tablets (Genesis Pharmaceuticals Ltd) Candesartan cilexetil 16mg Tablet Oral	02050502
Candesartan 16mg tablets Candesartan cilexetil 16mg Tablet Oral	02050502
Candesartan 16mg tablets (Waymade Healthcare Plc) Candesartan cilexetil 16mg Tablet Oral	02050502
Candesartan 16mg tablets (Sandoz Ltd) Candesartan cilexetil 16mg Tablet Oral	02050502
Candesartan 16mg/5ml oral solutionCandesartan cilexetil3.2mg/1mlOral solutionOral	02050502
Candesartan 16mg tablets (Zentiva)Candesartan cilexetil16mgTabletOral	02050502
Amias 16mg tablets (Lexon (UK) Ltd)Candesartan cilexetil16mgTabletOral	02050502

DESCRIPTION	BNF1
Sibelium 5mg tablets (Imported (Ireland)) Flunarizine dihydrochloride 5mg Tablet Oral	04065400
Flunarizine 5mg tablets Flunarizine dihydrochloride 5mg Tablet Oral	04065400
Flunarizine 5mg capsules Flunarizine dihydrochloride 5mg Capsule Oral	04065400
Flunarizine 10mg tablets Flunarizine dihydrochloride 10mg Tablet Oral	04065400

DESCRIPTION	BNF1
Migraleve tablets (McNeil Products Ltd) Not applicable Route of administration not applicable	04070200
Migraleve Pink tablets (McNeil Products Ltd) Buclizine hydrochloride/Paracetamol/Codeine phosphate 6.25mg + 500mg + 8mg Tablet Oral	04070200
Paramax tablets (Sanofi) Metoclopramide hydrochloride/Paracetamol 5mg + 500mg Tablet Oral	04070401
Paramax sachets (Sanofi) Paracetamol/Metoclopramide hydrochloride 500mg + 5mg Effervescent powder Oral	04070401
Paracetamol 500mg / Metoclopramide 5mg effervescent powder sachets sugar free Paracetamol/Metoclopramide hydrochloride 500mg + 5mg Effervescent powder Oral	04070401
Migravess forte 5mg+450mg Effervescent tablet (Bayer Plc) Citric Acid/Metoclopramide Hydrochloride/Aspirin/Sodium Bicarbonate 5mg+450mg Effervescent Tablet Oral	04065500
Paracetamol 500mg with codeine phosphate 8mg & buclizine 6.25mg Buclizine Hydrochloride/Codeine Phosphate/Paracetamol 500mg+8mg+6.25mg Tablets Oral	03040102

Metoclopramide with aspirin 5mg + 450mg Effervescent tablet Citric Acid/Metoclopramide Hydrochloride/Aspirin/Sodium Bicarbonate 5mg + 450mg Effervescent Tablet Oral	04065500
Codeine phosphate 8mg with paracetamol 500mg with buclizine 6.25mg tablets Buclizine Hydrochloride/Codeine Phosphate/Paracetamol 8mg+500mg+6.25mg Tablets Oral	03040102
Midrid (rpr) Capsule (Rhone-Poulenc Rorer Ltd) Paracetamol/Isometheptene mucate 325mg + 65mg Capsule Oral	04070100
Generic Migraleve Pink tablets Buclizine hydrochloride/Paracetamol/Codeine phosphate 6.25mg + 500mg + 8mg Tablet Oral	04070200
MigraMax oral powder sachets (Zentiva) Metoclopramide hydrochloride/Aspirin DL-Lysine 10mg + 900mg Powder Oral	04070100
Metoclopramide with paracetamol 5mg + 500mg Tablet Metoclopramide Hydrochloride/Paracetamol 5mg + 500mg Tablet Oral	04065500
Paracetamol 500mg / Domperidone 10mg tablets Paracetamol/Domperidone maleate 500mg + 10mg Tablet Oral	00000000
Domperamol tablets (Servier Laboratories Ltd) Paracetamol/Domperidone maleate 500mg + 10mg Tablet Oral	00000000
Ergotamine tartrate with cyclizine and caffeine tablets Caffeine/Cyclizine Hydrochloride/Ergotamine Tartrate Tablets Oral	03040103
Metoclopramide with aspirin 5mg + 325mg Effervescent tablet Citric Acid/Metoclopramide Hydrochloride/Aspirin/Sodium Bicarbonate 5mg + 325mg Effervescent Tablet Oral	04065500
Metoclopramide with paracetamol 5mg + 500mg Sachets Metoclopramide Hydrochloride/Paracetamol 5mg + 500mg Sachets Oral	04065500
Paracetamol 325mg / Isometheptene 65mg capsules Paracetamol/Isometheptene mucate 325mg + 65mg Capsule Oral	04070100
Migraleve Yellow tablets (McNeil Products Ltd) Paracetamol/Codeine phosphate 500mg + 8mg Tablet Oral	04070100
Aspirin 900mg / Metoclopramide 10mg oral powder sachets sugar free Metoclopramide hydrochloride/Aspirin DL-Lysine 10mg + 900mg Powder Oral	04070100
Migravess 5mg+325mg Effervescent tablet (Bayer Plc) Citric Acid/Metoclopramide Hydrochloride/Aspirin/Sodium Bicarbonate 5mg+325mg Effervescent Tablet Oral	04065500
Midrid 325mg/65mg capsules (DHP Healthcare Ltd) Paracetamol/Isometheptene mucate 325mg + 65mg Capsule Oral	04070100
Generic Migraleve tablets Not applicable Route of administration not applicable	04070200
Migraleve - 1 Tablet (Pfizer Consumer Healthcare Ltd) Buclizine Hydrochloride/Codeine Phosphate/Paracetamol Tablet Oral	03040102
Isometheptene mucate with paracetamol 65mg+325mg Capsule Paracetamol/Isometheptene Mucate 65mg+325mg Capsule Oral	04070100
Migril tablets (Wockhardt UK Ltd) Caffeine hydrate/Cyclizine hydrochloride/Ergotamine tartrate 100mg + 50mg + 2mg Tablet Oral	04070401
Migraleve Ultra 50mg tablets (McNeil Products Ltd) Sumatriptan succinate 50mg Tablet Oral	04070401
Generic Migril tablets Caffeine hydrate/Cyclizine hydrochloride/Ergotamine tartrate 100mg + 50mg + 2mg Tablet Oral	04070401
Paracetamol 500mg / Metoclopramide 5mg tablets Metoclopramide hydrochloride/Paracetamol 5mg + 500mg Tablet Oral	04070401
Migraleve - 2 8mg+500mg Tablet (Pfizer Consumer Healthcare Ltd) Paracetamol/Codeine phosphate 500mg + 8mg Tablet Oral	04070100
Metoclopramide with lysine acetylsalicylate 10mg + 900mg Oral solution Metoclopramide Hydrochloride/Aspirin Lysine 10mg + 900mg Oral Solution Oral	04065500

Paracetamol with codeine & buclizine tablet Buclizine Hydrochloride/Codeine Phosphate/Paracetamol Tablets Oral	03040102
Solpadeine Migraine Ibuprofen & Codeine tablets (Omega Pharma Ltd) Ibuprofen/Codeine phosphate 200mg + 12.8mg Tablet Oral	04070200
Femigraine Effervescent tablet (Nicholas Laboratories Ltd)Cyclizine Hydrochloride/AspirinEffervescent Tablet	03040103

DESCRIPTION	BNF1
Paracetamol 500mg tablets Paracetamol 500mg Tablet Oral	04070100
Paracetamol 500mg capsules Paracetamol 500mg Capsule Oral	04070100
Paracetamol 500mg soluble tablets Paracetamol 500mg Effervescent tablet Oral	04070100
Paracetamol 120mg/5ml oral solution paediatric sugar free Paracetamol 24mg/1ml Oral solution Oral	04070100
Paracetamol 250mg/5ml oral suspension Paracetamol 50mg/1ml Oral suspension Oral	04070100
Paracetamol 250mg/5ml oral suspension sugar free Paracetamol 250mg/5ml Suspension Sugar-Free Oral	04070100
Paracetamol 120mg/5ml oral suspension Paracetamol 120mg/5ml Oral Suspension Paediatric Oral	04070100
Paracetamol 250mg/5ml oral suspension sugar free Paracetamol 50mg/1ml Oral suspension Oral	04070100
Paracetamol 500mg caplets (A A H Pharmaceuticals Ltd) Paracetamol 500mg Tablet Oral	04070100
Paracetamol 120mg/5ml oral solution paediatric Paracetamol 24mg/1ml Oral solution Oral	04070100
Paracetamol 120mg/5ml oral suspension paediatric sugar free Paracetamol 24mg/1ml Oral suspension Oral	04070100
Paracetamol 120mg/5ml oral suspension paediatric Paracetamol 24mg/1ml Oral suspension Oral	04070100
Disprol 120mg/5ml Oral suspension (Reckitt Benckiser Healthcare (UK) Ltd) Paracetamol 120mg/5ml Oral Suspension Oral	04070100
Paracetamol 500mg/5ml oral suspension sugar free Paracetamol 500mg/5ml Suspension Sugar-Free Oral	04070100
Calpol infant 120mg/5ml Oral suspension (McNeil Products Ltd) Paracetamol 120mg/5ml Oral Suspension Oral	04070100
Paracetamol 500mg Tablet (Teva UK Ltd) Paracetamol 500mg Tablet Oral	04070100
Paracetamol 500mg caplets (Zentiva) Paracetamol 500mg Tablet Oral	04070100
Paracetamol 500mg caplets (Rusco Ltd) Paracetamol 500mg Tablet Oral	04070100
PARACETAMOL 125 MG MIX	00000000
PARACETAMOL 30 MG SUP	00000000
Panasorb 500mg Tablet (Sanofi-Synthelabo Ltd) Paracetamol 500mg Tablet Oral	04070100
Medinol For Children 120mg/5ml oral suspension (SSL International Plc) Paracetamol 24mg/1ml Oral suspension Oral	04070100
PARACETAMOL 1 GM SUP	00000000
Paracetamol 500mg caplets (Phoenix Healthcare Distribution	04070100

Ltd) Paracetamol 500mg Tablet Oral	
Paracetamol 500mg soluble tablets (A A H Pharmaceuticals Ltd) Paracetamol 500mg Effervescent tablet Oral	04070100
Paracetamol 500mg tablets (Zanza Specials International Ltd) Paracetamol 500mg Tablet Oral	04070100
Paracetamol 500mg capsules (Almus Pharmaceuticals Ltd) Paracetamol 500mg Capsule Oral	04070100
Paracetamol 500mg tablets (Actavis UK Ltd) Paracetamol 500mg Tablet Oral	04070100
Panaleve plus 120mg/5ml Oral suspension sugar free (Pinewood Healthcare) Paracetamol 120mg/5ml Oral Suspension Oral	04070100
Paravict 500mg tablets (Ecogen Europe Ltd) Paracetamol 500mg Tablet Oral	04070100
PARACETAMOL	00000000
Calpol Infant 120mg/5ml oral suspension 5ml sachets (McNeil Products Ltd) Paracetamol 24mg/1ml Oral suspension Oral	04070100
PARACETAMOL	00000000
PARACETAMOL 125 MG TAB	00000000
Paracetamol 500mg soluble tablets (Fannin UK Ltd) Paracetamol 500mg Effervescent tablet Oral	04070100
Paracetamol 500mg capsules (DE Pharmaceuticals) Paracetamol 500mg Capsule Oral	04070100
Disprol Paracetamol 120mg/5ml oral suspension (Reckitt Benckiser Healthcare (UK) Ltd) Paracetamol 24mg/1ml Oral suspension Oral	04070100
Junior Parapaed 120mg/5ml oral suspension sugar free colour free (Pinewood Healthcare) Paracetamol 24mg/1ml Oral suspension Oral	04070100
Panadol ActiFast Soluble tablets (GlaxoSmithKline Consumer Healthcare) Paracetamol 500mg Effervescent tablet Oral	04070100
Paracetamol 500mg caplets (Bristol Laboratories Ltd) Paracetamol 500mg Tablet Oral	04070100
Paracetamol 120mg/5ml oral suspension paediatric (Rosemont Pharmaceuticals Ltd) Paracetamol 24mg/1ml Oral suspension Oral	04070100
Paracetamol 120mg/5ml Oral solution (Teva UK Ltd) Paracetamol 24mg/1ml Oral solution Oral	04070100
Calpol Six Plus Fastmelts 250mg tablets (McNeil Products Ltd) Paracetamol 250mg Orodispersible tablet Oral	04070100
Paracetamol 120mg/5ml oral suspension paediatric sugar free (A A H Pharmaceuticals Ltd) Paracetamol 24mg/1ml Oral suspension Oral	04070100
Paracetamol 120mg/5ml oral solution paediatric sugar free (Alliance Healthcare (Distribution) Ltd) Paracetamol 24mg/1ml Oral solution Oral	04070100
Paracetamol 500mg soluble tablets (Kent Pharmaceuticals Ltd) Paracetamol 500mg Effervescent tablet Oral	04070100
PARACETAMOL SOLUBLE TAB	00000000
Calpol infant 120mg/5ml Oral suspension (McNeil Products Ltd) Paracetamol 120mg/5ml Oral Suspension Oral	04070100
Panadol Extra tablets (GlaxoSmithKline Consumer Healthcare) Paracetamol/Caffeine 500mg + 65mg Tablet Oral	04070100

Paracetamol 120 mg capsule Paracetamol 120mg Capsules Oral	04070100
PARACETAMOL 250 MG TAB	00000000
DISPROL PARACETAMOL SF	00000000
Paracetamol 500mg/5ml oral solution Paracetamol 100mg/1ml Oral solution Oral	04070100
Paracetamol 500mg caplets (Icarus Pharmaceuticals Ltd) Paracetamol 500mg Tablet Oral	04070100
Mandanol Infant paracetamol 120mg/5ml oral suspension (M & A Pharmachem Ltd) Paracetamol 24mg/1ml Oral suspension Oral	04070100
Paracetamol 500mg Tablet (Nucare Plc) Paracetamol 500mg Tablet Oral	04070100
Paracetamol 120mg/5ml oral solution paediatric sugar free (Pinewood Healthcare) Paracetamol 24mg/1ml Oral solution Oral	04070100
Mandanol 500mg tablets (M & A Pharmachem Ltd) Paracetamol 500mg Tablet Oral	04070100
PARACETAMOL 500 MG SUP	00000000
Paracetamol 500mg/50ml solution for infusion vials Paracetamol 10mg/1ml Solution for infusion Intravenous	04070100
Paracetamol 250mg/5ml oral suspension sugar free Paracetamol 250mg/5ml Oral Suspension Oral	04070100
Paracetamol 120mg/5ml oral suspension paediatric sugar free (Vantage) Paracetamol 24mg/1ml Oral suspension Oral	04070100
Calpol Six Plus 250mg/5ml oral suspension 5ml sachets sugar free (McNeil Products Ltd) Paracetamol 50mg/1ml Oral suspension Oral	04070100
Paracetamol 500mg caplets (J M McGill Ltd) Paracetamol 500mg Tablet Oral	04070100
Paracetamol 120mg/5ml oral solution paediatric (A A H Pharmaceuticals Ltd) Paracetamol 24mg/1ml Oral solution Oral	04070100
Paracetamol 500mg tablets (A A H Pharmaceuticals Ltd) Paracetamol 500mg Tablet Oral	04070100
Medinol Paediatric paracetamol 120mg/5ml oral suspension (SSL International Plc) Paracetamol 24mg/1ml Oral suspension Oral	04070100
Paracetamol 500mg tablets (Teva UK Ltd) Paracetamol 500mg Tablet Oral	04070100
Paracetamol 500mg capsules (Teva UK Ltd) Paracetamol 500mg Capsule Oral	04070100
Paracetamol 500mg caplets (Kent Pharmaceuticals Ltd) Paracetamol 500mg Tablet Oral	04070100
PARACETAMOL CO TAB	00000000
Paracetamol 240mg oral powder sachets sugar free Paracetamol 240mg Powder Oral	04070100
Paracetamol 1g oral powder sachets Paracetamol 1gram Powder Oral	04070100
Paracetamol 120mg/5ml Oral suspension (Co-Pharma Ltd) Paracetamol 120mg/5ml Oral Suspension Oral	04070100
Mandanol 500mg caplets (M & A Pharmachem Ltd) Paracetamol 500mg Tablet Oral	04070100
Paracetamol 500mg tablets (IVAX Pharmaceuticals UK Ltd)	04070100

Paracetamol 500mg Tablet Oral	
Paracetamol 500mg capsules (Zentiva) Paracetamol 500mg Capsule Oral	04070100
Paracetamol 500mg caplets (Vantage) Paracetamol 500mg Tablet Oral	04070100
PARACETAMOL	00000000
PARACETAMOL 60 MG SUP	00000000
Disprol Paracetamol 120mg soluble tablets (Reckitt Benckiser Healthcare (UK) Ltd) Paracetamol 120mg Soluble tablet Oral	04070100
Paracetamol 500mg soluble tablets (Almus Pharmaceuticals Ltd) Paracetamol 500mg Effervescent tablet Oral	04070100
Paracetamol 500mg caplets (Sigma Pharmaceuticals Plc) Paracetamol 500mg Tablet Oral	04070100
Panadol OA 1000mg tablets (GlaxoSmithKline Consumer Healthcare) Paracetamol 1gram Tablet Oral	04070100
Paracetamol 500mg soluble tablets (Actavis UK Ltd) Paracetamol 500mg Effervescent tablet Oral	04070100
Lloydspharmacy Paracetamol 120mg/5ml oral suspension sugar free (Lloyds Pharmacy Ltd) Paracetamol 24mg/1ml Oral suspension Oral	04070100
PARACETAMOL 100 MG SUP	00000000
PARACETAMOL 240 MG SUS	00000000
Galpamol 120mg/5ml Oral suspension (Galpharm International Ltd) Paracetamol 120mg/5ml Oral Suspension Oral	04070100
Paracetamol 500mg Tablet (Thornton & Ross Ltd) Paracetamol 500mg Tablet Oral	04070100
Paracetamol 500mg Tablet (Family Health) Paracetamol 500mg Tablet Oral	04070100
Paracetamol 500mg caplets (Teva UK Ltd) Paracetamol 500mg Tablet Oral	04070100
Infadrops 100mg/ml liquid (Mercury Pharma Group Ltd) Paracetamol 100mg/1ml Oral solution Oral	00000000
Paracetamol 500mg caplets (IVAX Pharmaceuticals UK Ltd) Paracetamol 500mg Tablet Oral	04070100
Calpol Six Plus 250mg/5ml oral suspension (McNeil Products Ltd) Paracetamol 50mg/1ml Oral suspension Oral	04070100
Panadol Extra Advance 500mg/65mg tablets (GlaxoSmithKline Consumer Healthcare) Paracetamol/Caffeine 500mg + 65mg Tablet Oral	04070100
Panadol 500mg Soluble tablet (GlaxoSmithKline Consumer Healthcare) Paracetamol 500mg Effervescent tablet Oral	04070100
Paracetamol 500mg capsules (Wockhardt UK Ltd) Paracetamol 500mg Capsule Oral	04070100
Perfalgan 500mg/50ml solution for infusion vials (Bristol-Myers Squibb Pharmaceuticals Ltd) Paracetamol 10mg/1ml Solution for infusion Intravenous	04070100
Paracetamol 500mg soluble tablets (Teva UK Ltd) Paracetamol 500mg Effervescent tablet Oral	04070100
Calpol Infant 120mg/5ml oral suspension sugar free (McNeil Products Ltd) Paracetamol 24mg/1ml Oral suspension Oral	04070100
Paracetamol 120mg soluble tablets sugar free (Ennogen Healthcare Ltd) Paracetamol 120mg Soluble tablet Oral	04070100
Paracetamol 120mg/5ml oral suspension 5ml sachets	04070100

Paracetamol 24mg/1ml Oral suspension Oral	
Paracetamol 1g tablets Paracetamol 1gram Tablet Oral	04070100
Paracetamol 500mg caplets (Numark Ltd) Paracetamol 500mg Tablet Oral	04070100
Calpol Infant 120mg/5ml oral suspension 5ml sachets sugar free (McNeil Products Ltd) Paracetamol 24mg/1ml Oral suspension Oral	04070100
Paracetamol 120mg/5ml oral solution paediatric sugar free (Sigma Pharmaceuticals Plc) Paracetamol 24mg/1ml Oral solution Oral	04070100
Paracetamol 120mg/5ml oral suspension sugar free Paracetamol 120mg/5ml Oral Suspension Paediatric Sugar-Free Oral	04070100
Paracetamol 500mg tablets (Aspar Pharmaceuticals Ltd) Paracetamol 500mg Tablet Oral	04070100
Calpol Paediatric 120mg/5ml oral suspension sugar free (McNeil Products Ltd) Paracetamol 24mg/1ml Oral suspension Oral	04070100
Paracetamol 120mg/5ml Oral solution (Celltech Pharma Europe Ltd) Paracetamol 24mg/1ml Oral solution Oral	04070100
Paracetamol 500mg capsules (Kent Pharmaceuticals Ltd) Paracetamol 500mg Capsule Oral	04070100
Panadol baby & infant 120mg/5ml Oral suspension (GlaxoSmithKline Consumer Healthcare) Paracetamol 120mg/5ml Oral Suspension Oral	04070100
Paracetamol 500mg Tablet (M & A Pharmachem Ltd) Paracetamol 500mg Tablet Oral	04070100
Paracetamol 500mg capsules (Sigma Pharmaceuticals Plc) Paracetamol 500mg Capsule Oral	04070100
Paracetamol 500mg capsules (Actavis UK Ltd) Paracetamol 500mg Capsule Oral	04070100
Paracetamol 500mg capsules (Waymade Healthcare Plc) Paracetamol 500mg Capsule Oral	04070100
Paracetamol 1g/100ml solution for infusion vials (A A H Pharmaceuticals Ltd) Paracetamol 10mg/1ml Solution for infusion Intravenous	04070100
Paracetamol 500mg caplets (Mawdsley-Brooks & Company Ltd) Paracetamol 500mg Tablet Oral	04070100
Paracetamol 250mg/5ml oral suspension sugar free (Vantage) Paracetamol 50mg/1ml Oral suspension Oral	04070100
Anadin Paracetamol 500mg tablets (Pfizer Consumer Healthcare Ltd) Paracetamol 500mg Tablet Oral	04070100
Paracetamol Capsule (Co-operative) Paracetamol 500mg Capsule Oral	04070100
Paracetamol 500mg/5ml oral suspension (Royal Preston Hospital mucilage formula) (Special Order) Paracetamol 100mg/1ml Oral suspension Oral	04070100
Paracetamol 500mg tablets (Vantage) Paracetamol 500mg Tablet Oral	04070100
Calpol infant 120mg/5ml Liquid (McNeil Products Ltd) Paracetamol 120mg/5ml Liquid Oral	04070100
Paracetamol 120mg/5ml Oral suspension sugar free (Pinewood Healthcare) Paracetamol 120mg/5ml Oral Suspension Oral	04070100

Paracetamol 500mg tablets (The Boots Company Plc) Paracetamol 500mg Tablet Oral	04070100
Paracetamol 500mg caplets (Almus Pharmaceuticals Ltd) Paracetamol 500mg Tablet Oral	04070100
Paracetamol 250mg/5ml oral suspension sugar free (Pinewood Healthcare) Paracetamol 50mg/1ml Oral suspension Oral	04070100
Calpol 120mg/5ml Liquid (Pfizer Consumer Healthcare Ltd) Paracetamol 120mg/5ml Liquid Oral	04070100
Paracetamol 120mg/5ml Oral solution (Rosemont Pharmaceuticals Ltd) Paracetamol 24mg/1ml Oral solution Oral	04070100
Paracetamol 500mg tablets (Kent Pharmaceuticals Ltd) Paracetamol 500mg Tablet Oral	04070100
Paracetamol 500mg caplets (Waymade Healthcare Plc) Paracetamol 500mg Tablet Oral	04070100
Medised plain 120mg/5ml Oral suspension (SSL International Plc) Paracetamol 120mg/5ml Oral Suspension	04070100
Medinol 120mg/5ml Oral suspension (SSL International Plc) Paracetamol 120mg/5ml Oral Suspension Oral	04070100
Paracetamol 500mg/50ml solution for infusion bottles Paracetamol 10mg/1ml Solution for infusion Intravenous	04070100
Paracetamol 500mg/5ml oral suspension sugar free Paracetamol 100mg/1ml Oral suspension Oral	04070100
Paracetamol 500mg soluble tablets (Waymade Healthcare Plc) Paracetamol 500mg Effervescent tablet Oral	04070100
Perfalgan 1g/100ml solution for infusion vials (Bristol-Myers Squibb Pharmaceuticals Ltd) Paracetamol 10mg/1ml Solution for infusion Intravenous	04070100
Paracetamol 120mg/5ml oral solution paediatric sugar free (Waymade Healthcare Plc) Paracetamol 24mg/1ml Oral solution Oral	04070100
Paracetamol 500mg Tablet (Co-operative) Paracetamol 500mg Tablet Oral	04070100
Tixymol 120mg/5ml Oral suspension (Novartis Consumer Health UK Ltd) Paracetamol 120mg/5ml Oral Suspension Oral	04070100
Paracetamol 500mg soluble tablets (Zentiva) Paracetamol 500mg Effervescent tablet Oral	04070100
Paracetamol 500mg soluble tablets (Alliance Healthcare (Distribution) Ltd) Paracetamol 500mg Effervescent tablet Oral	04070100
Children's Lemsip Cold & Flu Blackcurrant oral powder sachets (Reckitt Benckiser Healthcare (UK) Ltd) Paracetamol 240mg Powder Oral	04070100
Paracetamol 500mg/5ml oral solution sugar free Paracetamol 100mg/1ml Oral solution Oral	04070100
PARACETAMOL SOLUBLE TAB	00000000
Paracetamol 120mg/5ml Oral solution (William Ransom) Paracetamol 24mg/1ml Oral solution Oral	04070100
Paldesic paracetamol 120mg/5ml oral suspension (Rosemont Pharmaceuticals Ltd) Paracetamol 24mg/1ml Oral suspension Oral	04070100
PARACETAMOL 300 MG TAB	00000000
Paracetamol 500mg tablets (DE Pharmaceuticals) Paracetamol 500mg Tablet Oral	04070100
Lloydspharmacy Paracetamol Six Plus 250mg/5ml oral	04070100

suspension sugar free (Lloyds Pharmacy Ltd) Paracetamol 50mg/1ml Oral suspension Oral	
Panadol 500mg tablets (GlaxoSmithKline Consumer Healthcare) Paracetamol 500mg Tablet Oral	04070100
Paracetamol 250mg/5ml oral suspension sugar free Paracetamol 250mg/5ml Suspension Sugar-Free Oral	04070100
Paracetamol 120mg/5ml oral solution paediatric (Alliance Healthcare (Distribution) Ltd) Paracetamol 24mg/1ml Oral solution Oral	04070100
Paracetamol 50mg oral powder sachets sugar free Paracetamol 50mg Powder Oral	04070100
Medinol 120mg/5ml Oral suspension (SSL International Plc) Paracetamol 120mg/5ml Oral Suspension Oral	04070100
Medinol Under 6 paracetamol 120mg/5ml oral suspension (SSL International Plc) Paracetamol 24mg/1ml Oral suspension Oral	04070100
Paracetamol 500mg capsules (A A H Pharmaceuticals Ltd) Paracetamol 500mg Capsule Oral	04070100
Paracetamol 500mg caplets (Ennogen Healthcare Ltd) Paracetamol 500mg Tablet Oral	04070100
Paracetamol 1g effervescent tablets sugar free Paracetamol 1gram Effervescent tablet Oral	04070100
Paradote 100mg/500mg tablets (Sinclair IS Pharma Plc) Paracetamol 500mg Tablet Oral	04070100
Paracetamol 250mg/5ml oral suspension sugar free (A A H Pharmaceuticals Ltd) Paracetamol 50mg/1ml Oral suspension Oral	04070100
PARACETAMOL 240 MG ELI	00000000
Paracetamol 500mg tablets (Wockhardt UK Ltd) Paracetamol 500mg Tablet Oral	04070100
Paracetamol 500mg caplets (Accord Healthcare Ltd) Paracetamol 500mg Tablet Oral	04070100
Paracetamol 500mg capsules (Mawdsley-Brooks & Company Ltd) Paracetamol 500mg Capsule Oral	04070100
Paracetamol 500mg Tablet (M & A Pharmachem Ltd) Paracetamol 500mg Tablet Oral	04070100
Calpol Infant 120mg/5ml oral suspension (McNeil Products Ltd) Paracetamol 24mg/1ml Oral suspension Oral	04070100
Paracetamol 250mg/5ml oral suspension sugar free (Kent Pharmaceuticals Ltd) Paracetamol 50mg/1ml Oral suspension Oral	04070100
Paracetamol 500mg Capsule (A A H Pharmaceuticals Ltd) Paracetamol 500mg Capsule Oral	04070100
Medinol for children 120mg/5ml Oral suspension (SSL International Plc) Paracetamol 120mg/5ml Oral Suspension Oral	04070100
Paracetamol 500mg tablets (Sigma Pharmaceuticals Plc) Paracetamol 500mg Tablet Oral	04070100
Panadol 500mg capsules (GlaxoSmithKline Consumer Healthcare) Paracetamol 500mg Capsule Oral	04070100
Paracetamol 500mg tablets (Alliance Healthcare (Distribution) Ltd) Paracetamol 500mg Tablet Oral	04070100
Panadol Advance 500mg tablets (GlaxoSmithKline Consumer Healthcare) Paracetamol 500mg Tablet Oral	04070100

Paracetamol 500mg caplets (Actavis UK Ltd) Paracetamol 500mg Tablet Oral	04070100
Calpol Infant Sugar Free Colour Free 120mg/5ml oral suspension (McNeil Products Ltd) Paracetamol 24mg/1ml Oral suspension Oral	04070100
Paracetamol 120mg/5ml oral suspension paediatric sugar free (Pinewood Healthcare) Paracetamol 24mg/1ml Oral suspension Oral	04070100
Paracetamol 500mg caplets (Alliance Healthcare (Distribution) Ltd) Paracetamol 500mg Tablet Oral	04070100
Numark Paracetamol 500mg capsules (Numark Ltd) Paracetamol 500mg Capsule Oral	04070100
PARACETAMOL 75 MG SUS	00000000
Paracetamol 500mg caplets (AM Distributions (Yorkshire) Ltd) Paracetamol 500mg Tablet Oral	04070100
Paracetamol 250mg/5ml oral suspension (Rosemont Pharmaceuticals Ltd) Paracetamol 50mg/1ml Oral suspension Oral	04070100
Paracetamol 650mg oral powder sachets Paracetamol 650mg Powder Oral	04070100
Disprol 120mg/5ml Oral suspension (Reckitt Benckiser Healthcare (UK) Ltd) Paracetamol 120mg/5ml Oral Suspension Oral	04070100
Paracetamol 500mg capsules (Bristol Laboratories Ltd) Paracetamol 500mg Capsule Oral	04070100
Paracetamol 500mg Tablet (Aspar Pharmaceuticals Ltd) Paracetamol 500mg Tablet Oral	04070100
Paracetamol 1g/100ml solution for infusion vials Paracetamol 10mg/1ml Solution for infusion Intravenous	04070100
Placidex 120mg/5ml Oral solution (E C De Witt) Paracetamol 24mg/1ml Oral solution Oral	04070100
Galpamol for Children 120mg/5ml oral suspension 5ml sachets sugar free (Galpharm International Ltd) Paracetamol 24mg/1ml Oral suspension Oral	04070100
Paracetamol 1g/100ml solution for infusion bottles (B.Braun Melsungen AG) Paracetamol 10mg/1ml Solution for infusion Intravenous	04070100
Paracetamol 250mg/5ml oral suspension 5ml sachets sugar free Paracetamol 50mg/1ml Oral suspension Oral	04070100
Paracetamol 250mg/5ml oral suspension sugar free (Alliance Healthcare (Distribution) Ltd) Paracetamol 50mg/1ml Oral suspension Oral	04070100
Paracetamol 500mg/5ml Oral suspension sugar free (Rosemont Pharmaceuticals Ltd) Paracetamol 500mg/5ml Oral Suspension Oral	04070100
Paracetamol 120mg/5ml oral suspension paediatric sugar free (Thornton & Ross Ltd) Paracetamol 24mg/1ml Oral suspension Oral	04070100
Calpol Six Plus 250mg/5ml oral suspension sugar free (McNeil Products Ltd) Paracetamol 50mg/1ml Oral suspension Oral	04070100
Paracetamol 500mg capsules (Aspar Pharmaceuticals Ltd) Paracetamol 500mg Capsule Oral	04070100
Paracetamol 125mg/5ml syrup Paracetamol 125mg/5ml Syrup Oral	04070100

Paracetamol 500mg tablets (Galpharm International Ltd) Paracetamol 500mg Tablet Oral	04070100
Panadol ActiFast 500mg tablets (GlaxoSmithKline Consumer Healthcare) Paracetamol 500mg Tablet Oral	04070100
Paracetamol 100mg/ml oral solution sugar free Paracetamol 100mg/1ml Oral solution Oral	00000000
Paracetamol 120mg/5ml Oral suspension (Nucare Plc) Paracetamol 120mg/5ml Oral Suspension Oral	04070100
PARACETAMOL 150 MG SUP	00000000
Paracetamol 250mg/5ml oral solution Paracetamol 50mg/1ml Oral solution Oral	04070100
Femerital Tablet (Boehringer Mannheim UK Ltd) Paracetamol/Ambucetamide Tablet Oral	04070100
Paracetamol 500mg Tablet (Celltech Pharma Europe Ltd) Paracetamol 500mg Tablet Oral	04070100
Paracetamol 500mg capsules (Focus Pharmaceuticals Ltd) Paracetamol 500mg Capsule Oral	04070100
PARACETAMOL 125 MG ELI	00000000
Paracetamol 120mg/5ml oral suspension paediatric (A A H Pharmaceuticals Ltd) Paracetamol 24mg/1ml Oral suspension Oral	04070100
Paracetamol 500mg effervescent tablets Paracetamol 500mg Effervescent tablet Oral	04070100
Paracetamol 100mg/10ml solution for infusion ampoules Paracetamol 10mg/1ml Solution for infusion Intravenous	04070100
Paraclear 500mg Soluble tablet (Roche Consumer Health) Paracetamol 500mg Effervescent tablet Oral	04070100
PARACETAMOL 500 MG ELI	00000000
Paracetamol 120mg/5ml Oral suspension sugar free (IVAX Pharmaceuticals UK Ltd) Paracetamol 120mg/5ml Oral Suspension Oral	04070100
Paracetamol 120mg soluble tablets sugar free Paracetamol 120mg Soluble tablet Oral	00000000
PARACETAMOL 20 MG SUP	00000000
Paracetamol 500mg Tablet (Almus Pharmaceuticals Ltd) Paracetamol 500mg Tablet Oral	04070100
Medinol Over 6 paracetamol 250mg/5ml oral suspension (SSL International Plc) Paracetamol 50mg/1ml Oral suspension Oral	04070100
Boots Paracetamol 500mg caplets (The Boots Company Plc) Paracetamol 500mg Tablet Oral	04070100
Paracetamol 120mg/5ml oral solution paediatric (Thornton & Ross Ltd) Paracetamol 24mg/1ml Oral solution Oral	04070100
Hedex 500mg tablets (Omega Pharma Ltd) Paracetamol 500mg Tablet Oral	04070100
Calpol six plus 250mg/5ml Oral suspension sugar free (McNeil Products Ltd) Paracetamol 250mg/5ml Oral Suspension Oral	04070100
Paracetamol 250mg/5ml oral suspension (A A H Pharmaceuticals Ltd) Paracetamol 50mg/1ml Oral suspension Oral	04070100
Paracetamol 120mg/5ml oral suspension paediatric sugar free (Alliance Healthcare (Distribution) Ltd) Paracetamol 24mg/1ml Oral suspension Oral	04070100
Paracetamol 500mg capsules (Almus Pharmaceuticals Ltd)	00000000

Paracetamol 500mg Capsule Oral	
Paracetamol 500mg/5ml oral suspension Paracetamol 100mg/1ml Oral suspension Oral	04070100
Paracetamol powder Paracetamol Powder	50000000
Paracetamol 500mg caplets (Lloyds Pharmacy Ltd) Paracetamol 500mg Tablet Oral	04070100
Paracetamol 250mg/5ml Liquid (Co-Pharma Ltd) Paracetamol 50mg/1ml Oral suspension Oral	04070100
Paracetamol 500mg tablets (Zentiva) Paracetamol 500mg Tablet Oral	04070100
Paracetamol 500mg tablets (Accord Healthcare Ltd) Paracetamol 500mg Tablet Oral	04070100
Paracets 500mg capsules (Sussex Pharmaceutical Ltd) Paracetamol 500mg Capsule Oral	04070100
Paldesic paracetamol 250mg/5ml oral suspension (Rosemont Pharmaceuticals Ltd) Paracetamol 50mg/1ml Oral suspension Oral	04070100
Paracetamol oral liquid Paracetamol Oral Liquid Oral	04070100
Panadol Ultra 12.8mg/500mg tablets (GlaxoSmithKline Consumer Healthcare) Paracetamol/Codeine phosphate 500mg + 12.8mg Tablet Oral	04070100
Paracetamol 500mg capsules (Galpharm International Ltd) Paracetamol 500mg Capsule Oral	04070100
Paracetamol 500mg/5ml oral suspension sugar free (A A H Pharmaceuticals Ltd) Paracetamol 100mg/1ml Oral suspension Oral	04070100
Paracetamol 120mg/5ml oral suspension 5ml sachets sugar free Paracetamol 24mg/1ml Oral suspension Oral	04070100
Paracetamol 1g/100ml solution for infusion bottles Paracetamol 10mg/1ml Solution for infusion Intravenous	04070100
Panadol Extra soluble tablets (GlaxoSmithKline Consumer Healthcare) Paracetamol/Caffeine 500mg + 65mg Effervescent tablet Oral	04070100
Paracetamol 500mg caplets (Ethigen Ltd) Paracetamol 500mg Tablet Oral	04070100
Flu strength hot lemon 1g Powder (A A H Pharmaceuticals Ltd) Paracetamol 1gram Powder Oral	04070100
Paracetamol 120mg/5ml Oral solution sugar free (A A H Pharmaceuticals Ltd) Paracetamol 24mg/1ml Oral solution Oral	04070100
Disprol paracetamol 120mg/5ml Oral suspension (Reckitt Benckiser Healthcare (UK) Ltd) Paracetamol 120mg/5ml Oral Suspension Oral	04070100
Flu Strength Hot Lemon Powders 1g oral powder sachets (Bell	Sons & Co (Druggists) Ltd) Paracetamol 1gram Powder Oral
Paracetamol 500mg caplets (Wockhardt UK Ltd) Paracetamol 500mg Tablet Oral	04070100
Tramil 500mg Capsule (Wyeth Consumer Healthcare) Paracetamol 500mg Capsule Oral	04070100
Paracetamol 500mg Tablet (OBG Pharmaceuticals Ltd) Paracetamol 500mg Tablet Oral	04070100
Paracetamol 500mg caplets (Galpharm International Ltd) Paracetamol 500mg Tablet Oral	04070100

Boots Paracetamol 500mg capsules (The Boots Company Plc) Paracetamol 500mg Capsule Oral	04070100
Paracets 500mg Tablet (Sussex Pharmaceutical Ltd) Paracetamol 500mg Tablet Oral	04070100
Medinol 250mg/5ml Oral suspension (SSL International Plc) Paracetamol 250mg/5ml Oral Suspension Oral	04070100
Paracetamol 250mg orodispersible tablets sugar free Paracetamol 250mg Orodispersible tablet Oral	04070100
PARACETAMOL 1 GM TAB	00000000
Obimol 500mg Tablet (Ayrton Saunders Ltd) Paracetamol 500mg Tablet Oral	04070100
Paracetamol 250mg/5ml oral suspension sugar free (DE Pharmaceuticals) Paracetamol 50mg/1ml Oral suspension Oral	04070100
Mandanol 6+ paracetamol 250mg/5ml oral suspension (M & A Pharmachem Ltd) Paracetamol 50mg/1ml Oral suspension Oral	04070100
Paracetamol 500mg caplets (Crescent Pharma Ltd) Paracetamol 500mg Tablet Oral	04070100
Paracetamol 120mg/5ml oral suspension paediatric sugar free (Kent Pharmaceuticals Ltd) Paracetamol 24mg/1ml Oral suspension Oral	04070100
Paracetamol 120mg/5ml oral solution paediatric sugar free (A A H Pharmaceuticals Ltd) Paracetamol 24mg/1ml Oral solution Oral	04070100
Paracetamol 120mg/5ml oral suspension sugar free Paracetamol 120mg/5ml Oral Suspension Oral	04070100
Paracetamol 500mg capsules (Lloyds Pharmacy Ltd) Paracetamol 500mg Capsule Oral	04070100
Paracetamol 1g/5ml oral suspension Paracetamol 200mg/1ml Oral suspension Oral	04070100
Panadol Period Pain 500mg/65mg tablets (GlaxoSmithKline Consumer Healthcare)Paracetamol/Caffeine500mg + 65mgTabletOral	04070100
Paracetamol 500mg tablets (Genesis Pharmaceuticals Ltd)Paracetamol500mgTabletOral	04070100
Paracetamol 500mg/5ml oral solution sugar free (Advanz Pharma)Paracetamol100mg/1mlOral solutionOral	04070100
Paracetamol 1g/5ml oral solutionParacetamol200mg/1mlOral solutionOral	04070100
Paracetamol 500mg/5ml oral suspension sugar free (DE Pharmaceuticals)Paracetamol100mg/1mlOral suspensionOral	04070100
Altridexamol 1000mg effervescent tablets (TriOn Pharma Ltd)Paracetamol1gramEffervescent tabletOral	04070100
Paracetamol 250mg/5ml oral suspension sugar free (Dowelhurst Ltd)Paracetamol50mg/1mlOral suspensionOral	04070100

DESCRIPTION	BNF1
Pizotifen 1.5mg tablets Pizotifen hydrogen malate 1.5mg Tablet Oral	04070402
Pizotifen 500microgram tablets Pizotifen hydrogen malate 500microgram Tablet Oral	04070402
Pizotifen 250micrograms/5ml oral solution sugar free Pizotifen hydrogen malate 50microgram/1ml Oral solution Oral	04070402

Pizotifen 1.5mg tablets (Teva UK Ltd) Pizotifen hydrogen malate 1.5mg Tablet Oral	04070402
SANOMIGRAN	00000000
Sanomigran 500microgram tablets (Novartis Pharmaceuticals UK Ltd) Pizotifen hydrogen malate 500microgram Tablet Oral	04070402
Pizotifen 1.5mg tablets (Almus Pharmaceuticals Ltd) Pizotifen hydrogen malate 1.5mg Tablet Oral	04070402
Sanomigran 1.5mg tablets (Novartis Pharmaceuticals UK Ltd) Pizotifen hydrogen malate 1.5mg Tablet Oral	04070402
Pizotifen 500micrograms/5ml oral suspension Pizotifen hydrogen malate 100microgram/1ml Oral suspension Oral	04070402
Pizotifen 1.5mg Tablet (Neo Laboratories Ltd) Pizotifen hydrogen malate 1.5mg Tablet Oral	04070402
Sanomigran 0.25mg/5ml elixir (Novartis Pharmaceuticals UK Ltd) Pizotifen hydrogen malate 50microgram/1ml Oral solution Oral	04070402
Pizotifen 250micrograms/5ml oral suspension Pizotifen hydrogen malate 50microgram/1ml Oral suspension Oral	04070402
PIZOTIFEN	00000000
Pizotifen 500microgram tablets (Teva UK Ltd) Pizotifen hydrogen malate 500microgram Tablet Oral	04070402
Pizotifen 1.5mg tablets (Actavis UK Ltd) Pizotifen hydrogen malate 1.5mg Tablet Oral	04070402
Pizotifen 1mg/5ml oral suspension Pizotifen hydrogen malate 200microgram/1ml Oral suspension Oral	04070402
Pizotifen 500microgram tablets (Almus Pharmaceuticals Ltd) Pizotifen hydrogen malate 500microgram Tablet Oral	04070402
Pizotifen 0.5mg Tablet (Neo Laboratories Ltd) Pizotifen hydrogen malate 500microgram Tablet Oral	04070402
Pizotifen 250micrograms/5ml oral solution Pizotifen hydrogen malate 50microgram/1ml Oral solution Oral	04070402
Pizotifen 500microgram tablets (A A H Pharmaceuticals Ltd) Pizotifen hydrogen malate 500microgram Tablet Oral	04070402
SANOMIGRAN	00000000
Pizotifen 1.5mg tablets (A A H Pharmaceuticals Ltd) Pizotifen hydrogen malate 1.5mg Tablet Oral	04070402
Sanomigran 1.5mg tablets (Mawdsley-Brooks & Company Ltd) Pizotifen hydrogen malate 1.5mg Tablet Oral	04070402
Pizotifen 500microgram tablets (Actavis UK Ltd) Pizotifen hydrogen malate 500microgram Tablet Oral	04070402
Pizotifen 1.5mg tablets (Phoenix Healthcare Distribution Ltd)Pizotifen hydrogen malate1.5mgTabletOral	04070402
