# Supplementary material for: Trends in the prevalence and pharmacological management of migraine during pregnancy in the UK, 2000–2018
Source: J Neurol Neurosurg Psychiatry. 2024 Apr 3;95(10):938–46. doi: 10.1136/jnnp-2024-333530 (PMC11420713; doi:10.1136/jnnp-2024-333530)
Supplement: Supplementary data [file jnnp-2024-333530supp001.pdf]

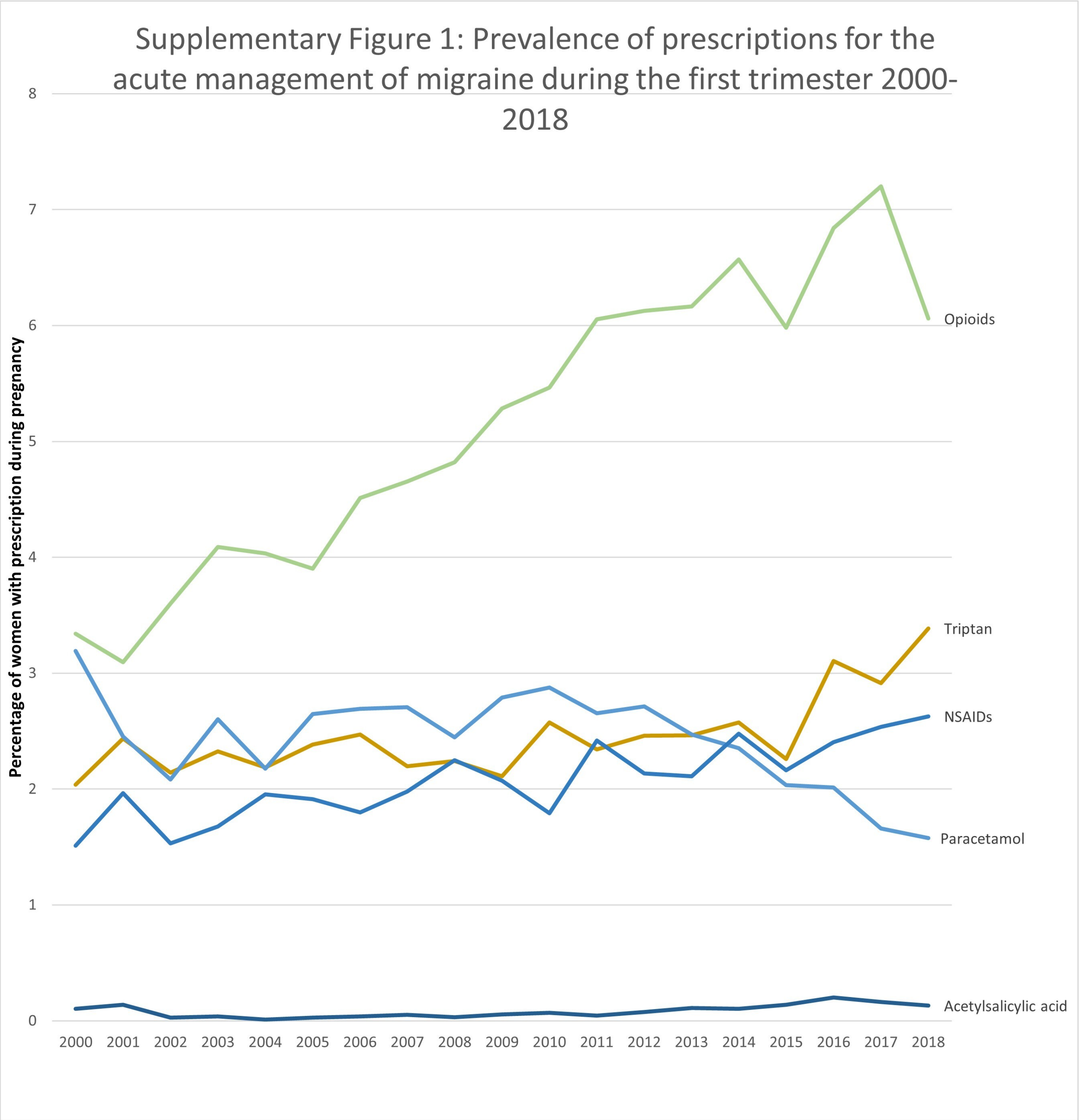
Supplementary Figure 1: Prevalence of prescriptions for the acute management of migraine during the first trimester 2000-2018
Percentage of women with prescription during pregnancy
0
1
2
3
4
5
6
7
8
2000 2001 2002 2003 2004 2005 2006 2007 2008 2009 2010 2011 2012 2013 2014 2015 2016 2017 2018
Opioids
Triptan
NSAIDs
Paracetamol
Acetylsalicylic acid
