# Supplementary figures and images for: Trends in the prevalence and pharmacological management of migraine during pregnancy in the UK, 2000–2018
Source: J Neurol Neurosurg Psychiatry. 2024 Apr 3;95(10):938–46. doi: 10.1136/jnnp-2024-333530 (PMC11420713; doi:10.1136/jnnp-2024-333530)

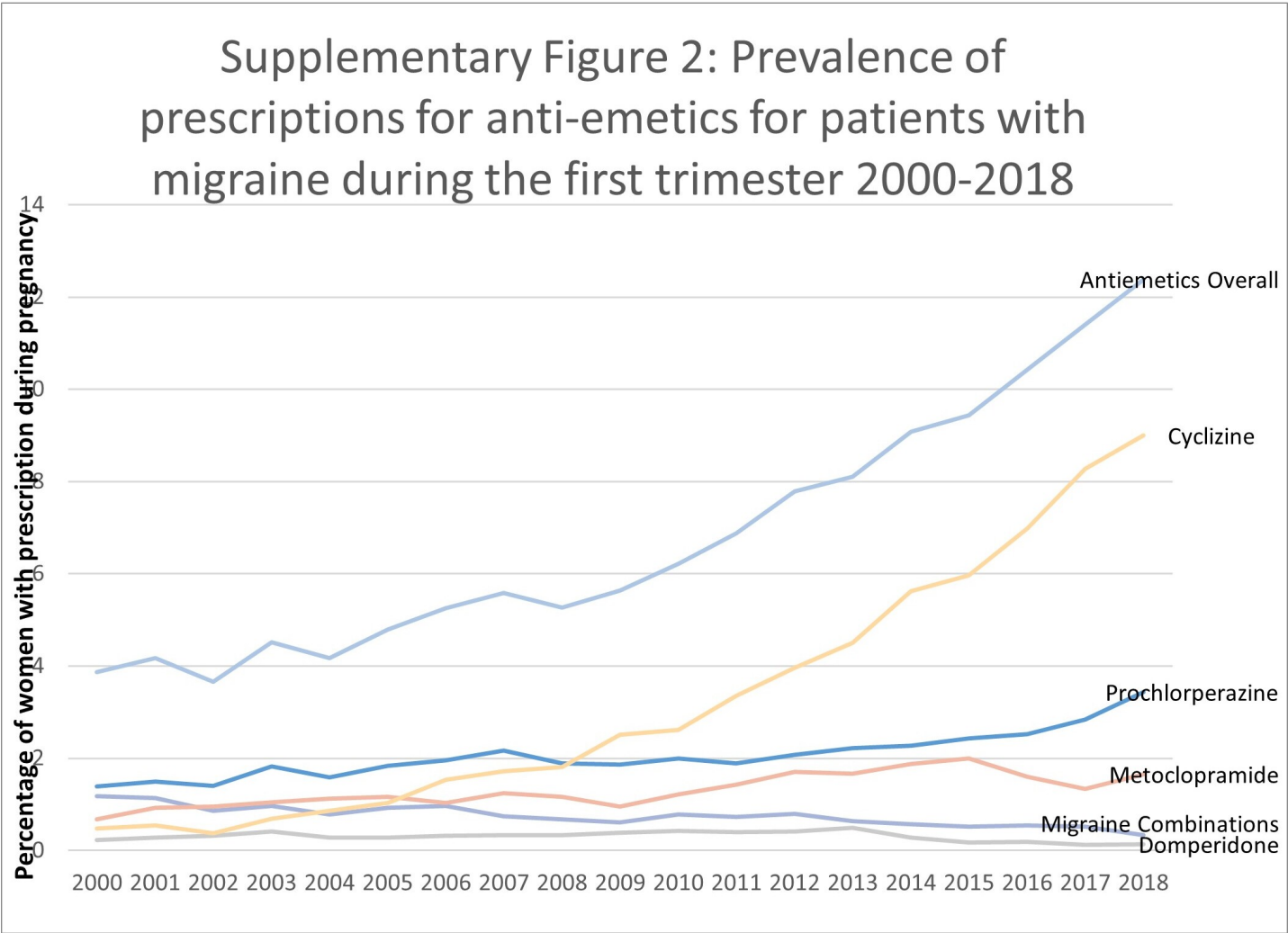

Supplement: Supplementary data [file jnnp-2024-333530supp002.pdf]

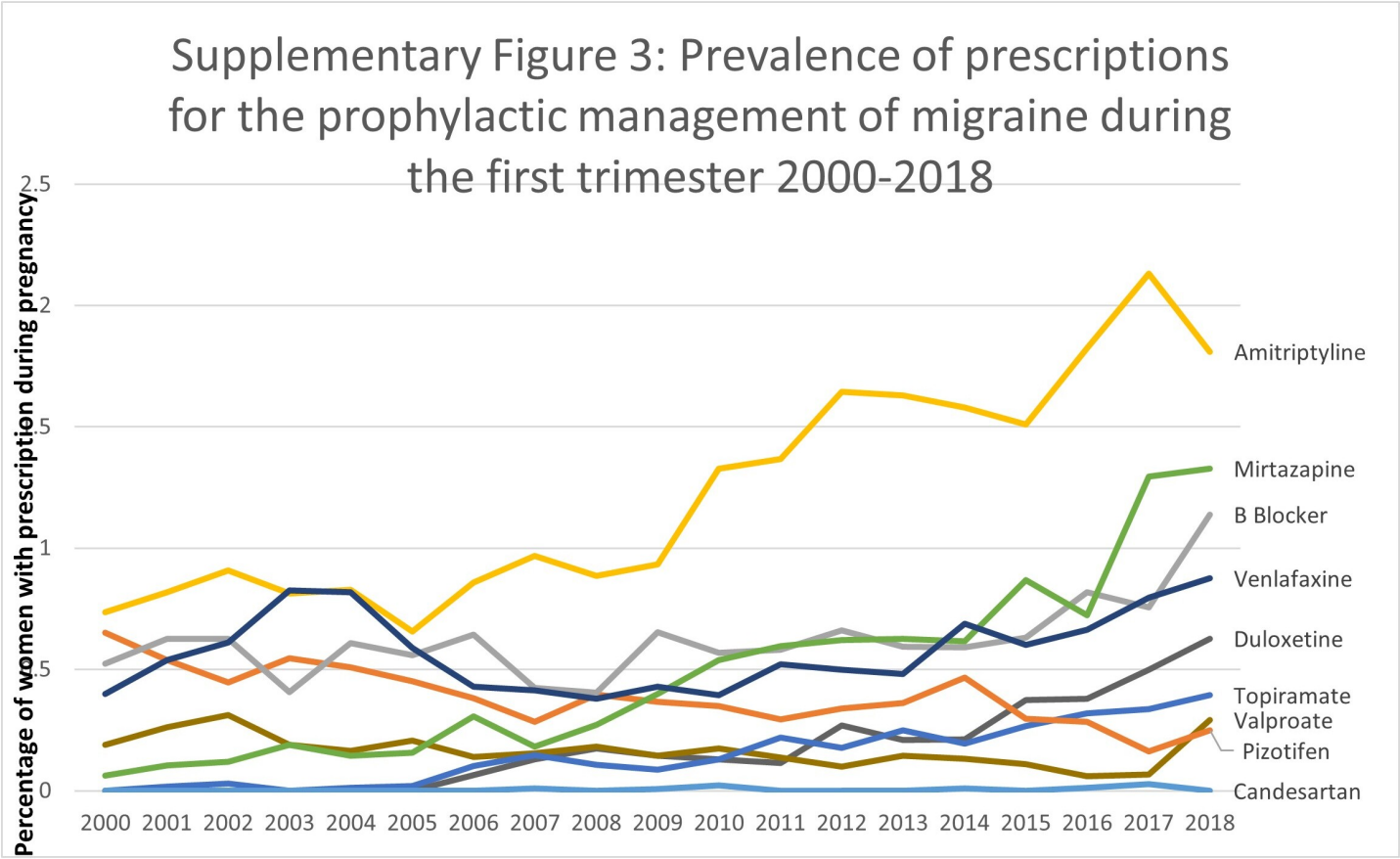

Supplement: Supplementary data [file jnnp-2024-333530supp003.pdf]
